# Targeting RBM41-mediated CKMT1B mRNA stability triggers disulfidptosis and suppresses tongue squamous cell carcinoma

**DOI:** 10.1038/s41419-026-09166-2

**Published:** 2026-07-31

**Authors:** Boqiang Tao, Ming Hao, Yang Hao, Shuhan Zhang, Zhaorong Xiao, He Shi, Qirong Li, Yiwen Qin, Dongxu Wang, Weiwei Liu

**Affiliations:** 1 Department of Oral and Maxillofacial Surgery, Hospital of Stomatology, Jilin University, Changchun, China; 2 Jilin Provincial Key Laboratory of Oral Biomedical Engineering, Hospital of Stomatology, Jilin University, Changchun, China; 3 Laboratory Animal Center, College of Animal Science, Jilin University, Changchun, China

**Keywords:** Drug screening, Oral cancer

## Abstract

Clarifying the mechanisms by which metabolic stress induces cell death in tumor cells is essential for the development of effective therapies targeting tongue squamous cell carcinoma (TSCC). In this study, we demonstrate that the RNA-binding motif protein 41 (RBM41) stabilizes CKMT1B mRNA through its RRM1 domain, thereby conferring resistance to disulfidptosis under conditions of glucose deprivation in TSCC. We explored the expression and functional role of RBM41 using both in vitro and in vivo models, which included RBM41-knockout, RRM1-domain-knockout, and RBM41-overexpressing TSCC cell lines. Our findings reveal that RBM41-mediated stabilization of CKMT1B mRNA is crucial for protecting TSCC cells from disulfidptosis during metabolic stress. Based on this mechanism, we designed a structure-specific inhibitory small molecule, L759-0762, which disrupts the interaction between RBM41 and CKMT1B mRNA. L759-0762 selectively sensitizes TSCC cells to disulfidptosis under glucose deprivation by abolishing the protective function of RBM41. Collectively, our findings identify RBM41 as a viable therapeutic target and demonstrate that pharmacological disruption of its interaction with CKMT1B mRNA represents a promising strategy for inducing disulfidptosis in TSCC.

**Figure:**
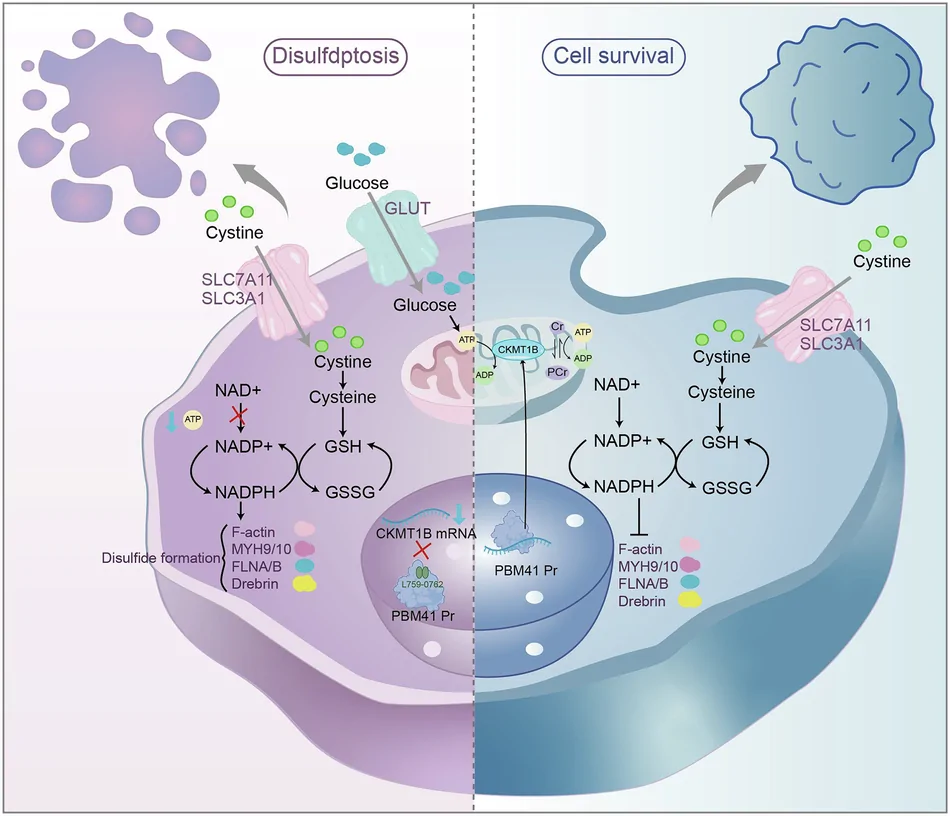

## Introduction

Metabolic reprogramming is a key feature of cancer progression that facilitates the rapid proliferation and survival of tumor cells in nutrient-poor microenvironments [1]. The classic Warburg effect, characterized by elevated glycolysis even in the presence of oxygen, is a well-established hallmark of many cancers, including tongue squamous cell carcinoma (TSCC) [2, 3]. Cancer cells can consume both glucose and glutamine to generate NADPH, which is the primary cellular reductant for biosynthesis and antioxidant pathways. It plays a crucial role in scavenging the reactive oxygen species (ROS) generated during rapid proliferation by restoring the reducing power of glutathione and thioredoxin [4]. Disulfidptosis, a newly discovered form of regulated cell death, is triggered by the critical depletion of NADPH due to glucose deprivation or impaired glucose uptake [5]. The ensuing redox imbalance leads to oxidative stress and aberrant disulfide bond formation within actin cytoskeleton proteins such as β-actin and FLNB [6]. These crosslinked proteins eventually dissociate from the cell membrane, resulting in rapid cell death. Therefore, disulfidptosis offers a viable new approach for tumor therapy. Nevertheless, the molecular mechanisms underlying disulfidptosis sensitivity, NADPH homeostasis, and the adaptive provisions against disulfide stress in cancer cells remain unexplored.

RNA-binding proteins (RBPs) mediate post-transcriptional regulatory processes through specific and non-specific binding to RNA molecules [7]. The critical functions of RBPs in tumorigenesis and metabolic adaptation are increasingly being acknowledged [8]. While several RBPs have been identified that influence cell fate regulation and apoptosis, their role in disulfidptosis is unclear [9]. Identifying disulfidptosis-related RBPs could potentially facilitate the development of targeted therapies for inducing disulfidptotic death in TSCC cells. RNA-binding motif protein 41 (RBM41) has a presumed yet poorly defined role in RNA metabolism, and its expression and functional significance in cancer, particularly in TSCC, remain completely unknown [10].

This study confirmed that RBM41 is genomically amplified and significantly overexpressed in TSCC, which correlates with aggressive tumor progression. RBM41 acts as a master regulator of cellular metabolism; the loss of RBM41 or specific ablation of its RNA-binding RRM1 domain sensitized TSCC cells to glucose deprivation by triggering disulfidptosis. CKMT1B, which encodes a mitochondrial creatine kinase critical for energy transduction, was identified as a primary direct target of RBM41. The latter binds to and stabilizes CKMT1B mRNA, which maintains ATP and NADPH homeostasis to protect cells from disulfide stress. L759-0762 selectively induced disulfidptosis in TSCC cells under metabolic stress by disrupting the interaction between RBM41 and CKMT1B, while sparing normal cells. Taken together, RBM41-CKMT1B-disulfidptosis axis presents a promising therapeutic target in TSCC, and selective inhibition of RBM41 can sensitize TSCC cells to disulfidptosis.

## Results

### RBM41 is significantly upregulated in TSCC

**Fig. 1:**
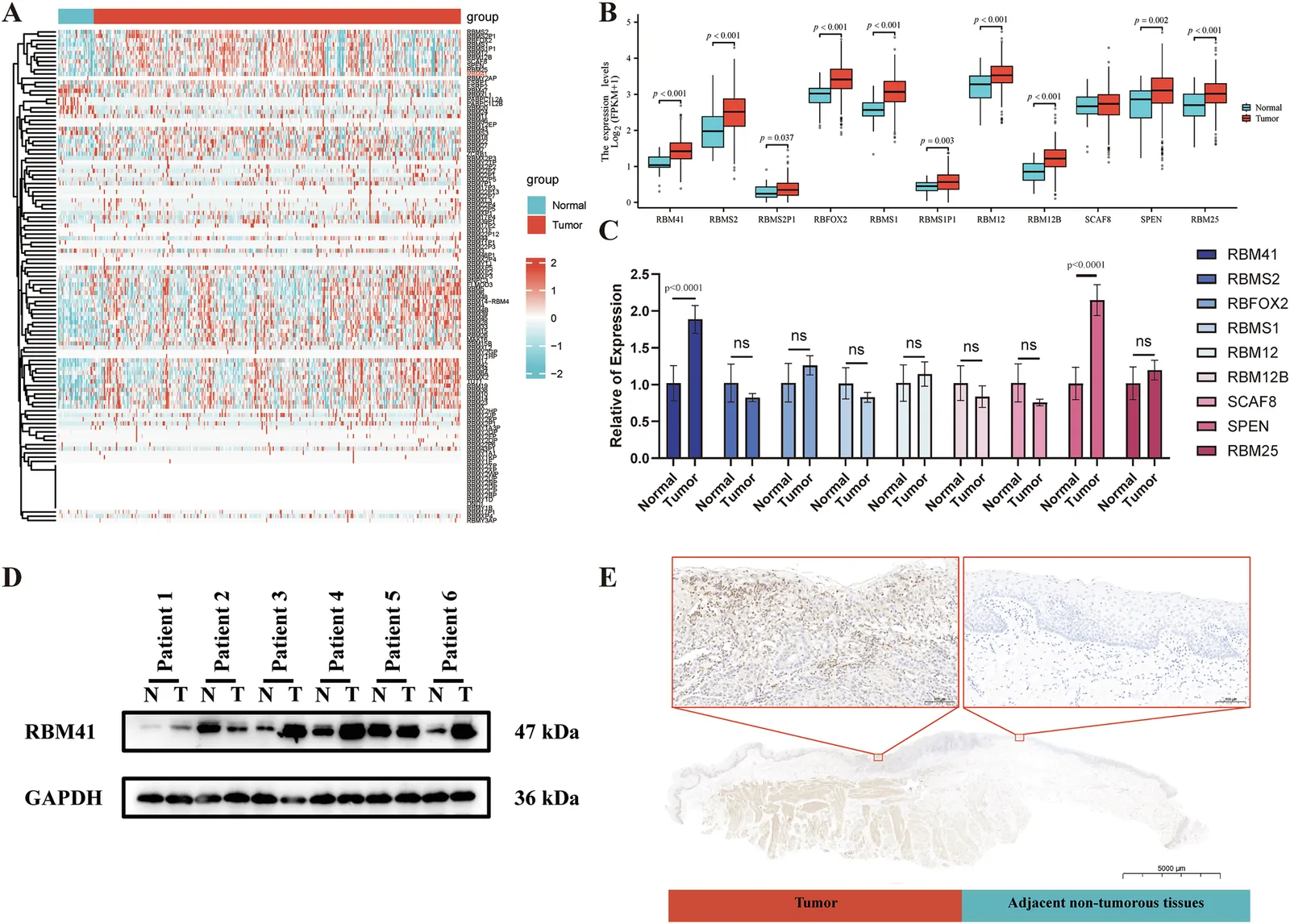
RBM41 is overexpressed in tongue squamous cell carcinoma. **A** Heatmap showing the expression profiles of RBM genes in OSCC tumor tissues compared to normal adjacent tissues from TCGA dataset. *n* = 362 tumor, *n* = 32 normal. **B** Non-paired sample (grouped) analysis. The expression profiles of 11 significantly dysregulated RBM genes in OSCC tumor tissues compared to normal adjacent tissues from the TCGA dataset. *n* = 330 tumor samples, *n* = 32 normal samples. Error bars represent mean ± SEM. Significance was determined using the Wilcoxon rank sum test. *P* values are as indicated. **C** qRT-PCR validation of 11 significantly dysregulated RBM genes mRNA levels in clinical TSCC tumor tissues compared to matched adjacent normal tissues. *n* = 3 biological replicates. Error bars represent mean ± SD. Significance was determined using a Two-way ANOVA test. *P* values are as indicated. **D** Western blot analysis of RBM41 protein expression in six paired TSCC tumors (T) and adjacent normal tissues (N). **E** IHC images showing RBM41 protein staining in TSCC tissue and adjacent normal epithelium. Scale bar = 5000 μm. The red framed squares refer to the area where the enlarged image is located; scale bar = 100 μm. Error bars represent mean ± SD. Significance was determined using a one-way ANOVA with Tukey’s multiple comparisons test (**C**). *P* values are as indicated.

To investigate the potential roles of RBM proteins in TSCC pathogenesis, the expression levels of 124 RBM genes in the OSCC dataset from TCGA were analyzed (Fig. 1A). This initial screening identified 11 RBM genes, including RBM41, RBMS1, RBMS2, RBM12, and SPEN, that were significantly dysregulated in OSCC tissues compared to normal controls (Fig. 1B). Only the RBM41 and SPEN transcripts were consistently and significantly upregulated in the TSCC tissues (Fig. 1C). To confirm this finding at the protein level, RBM41 protein expression in six paired TSCC and adjacent non-tumorous tissues were analyzed, and observed a marked upregulation in the tumor tissues (Fig. 1D). Furthermore, immunohistochemistry (IHC) images of a larger set of TSCC specimens showed intense nuclear and cytoplasmic staining of RBM41 in cancer cells, compared to weak or negative staining in normal epithelial cells (Fig. 1E). Survival analysis results based on the TCGA indicate that patients in the high expression group of RBM41 have a mortality risk 1.26 times that of the low expression group, with a 95% confidence interval of 0.89–1.79 and a *P*-value of 0.187 (Supplementary Fig. 1A). Overall, these findings suggest that RBM41 is significantly overexpressed in TSCC cells at both the transcriptomic and protein levels, and may contribute to malignant progression.

### RBM41 knockdown suppressed proliferation and invasion of TSCC cells

**Fig. 2:**
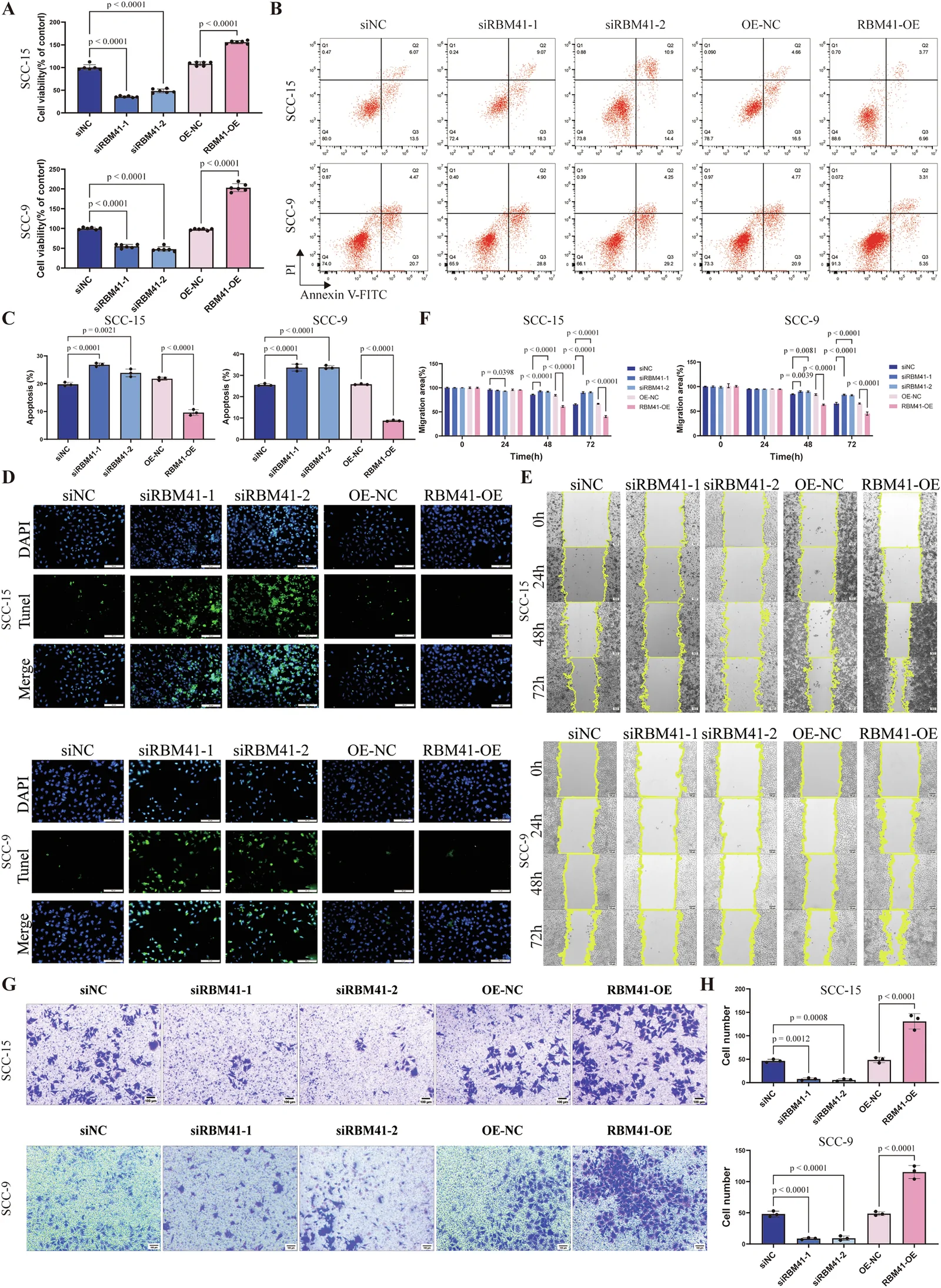
RBM41 drives TSCC cell proliferation, migration, and invasion in vitro. **A** CCK-8 assay assessing cell proliferation in SCC-15 and SCC-9 cells with RBM41 knockdown or overexpression. *n* = 6 biological replicates. Flow cytometry analysis of apoptosis using Annexin V/PI staining (**B**) and quantification (**C**), *n* = 3 biological replicates. **D** TUNEL assay showing apoptosis in SCC-15 and SCC-9 cells. Scale bar = 50 μm. Wound healing migration assay (**E**) and quantification of the relative wound closure area (**F**) in SCC-15 and SCC-9 cells. Transwell invasion assay (**G**) and quantification of invaded cells (**H**) in SCC-15 and SCC-9 cells. *n* = 3 biological replicates. Error bars represent mean ± SD. Significance was determined using a one-way ANOVA with Tukey’s multiple comparisons test (**A**, **C**, **H**) or two-way ANOVA with Tukey’s multiple comparisons test (**F**). *P* values are as indicated.

The biological functions of RBM41 in TSCC cells were further explored through in vitro experiments. First, RBM41 protein expression in three normal epithelial cell lines and three tongue cancer cell lines was analyzed. The SCC-15 and SCC-9 cell lines exhibited high expression levels of RBM41 and were selected for the subsequent experiments (Supplementary Fig. 2A, B). The changes in mRNA and protein levels were confirmed 48 h after transfection (Supplementary Fig. 2C–E), and the proliferation, apoptosis, migration, and invasion of TSCC cells after transient transfection were analyzed by standard functional assays (Fig. 2). RBM41 knockdown inhibited the proliferation, migration, and invasion of SCC-15 and SCC-9 cells, while its overexpression led to enhanced proliferation rates. Knockdown of RBM41 significantly increased the percentage of apoptotic SCC-15 and SCC-9 cells, while its overexpression markedly inhibited apoptosis.

### RBM41 knockout may alter the metabolism of TSCC cells via degradation of key metabolic mRNAs

**Fig. 3:**
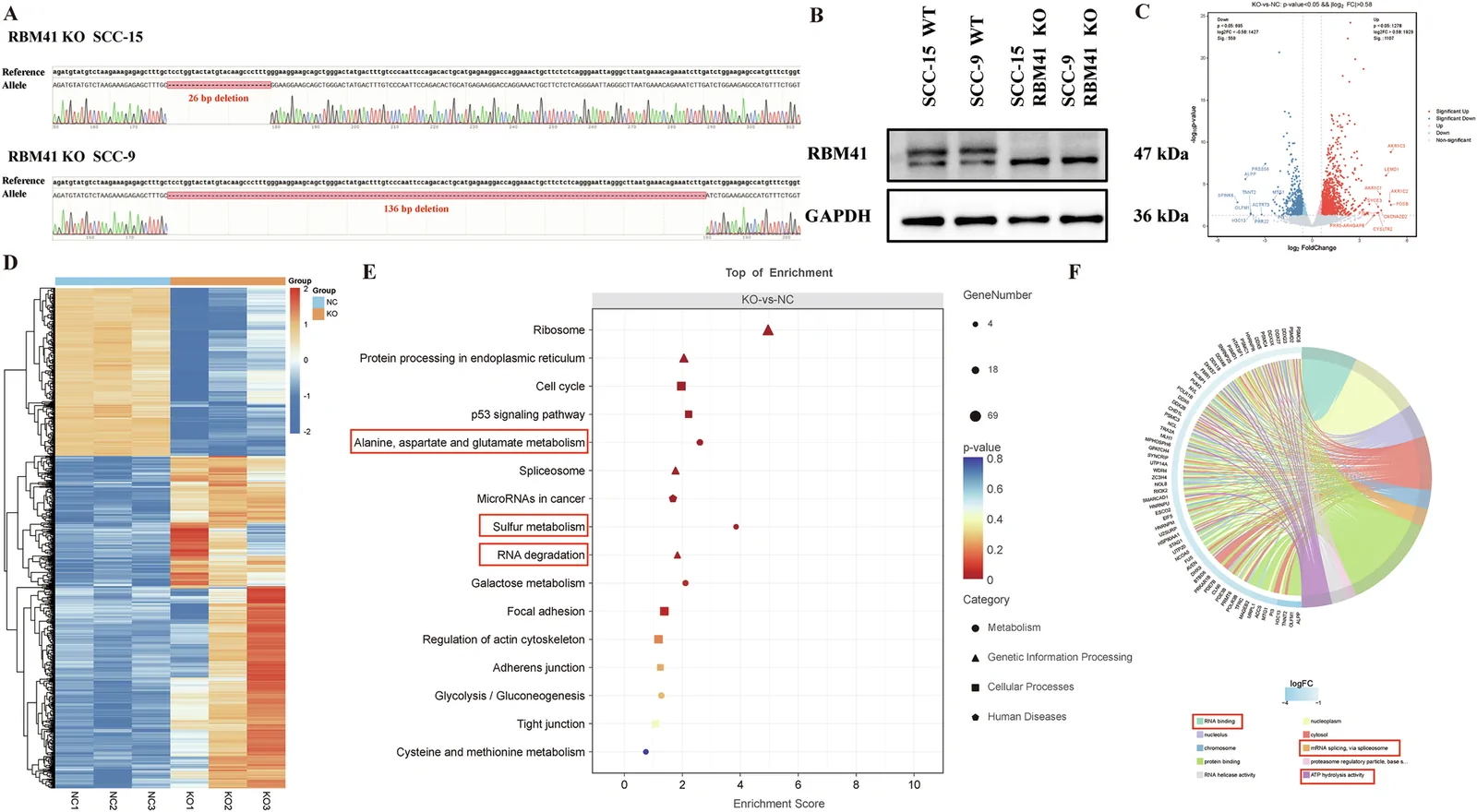
RBM41 knockout perturbs cellular metabolism and RNA degradation pathways. **A** Schematic diagram of the sgRNA targeting exon 3 of RBM41 and the sequencing chromatogram confirming frameshift mutations in the RBM41 KO clones. **B** Western blot confirming the loss of RBM41 protein expression in the KO cells. **C** Volcano plot of differentially expressed genes (DEGs) between RBM41 KO and wild-type (WT) cells. **D** Heatmap of the DEGs showing clear separation between RBM41 KO and WT samples. Kyoto Encyclopedia of Genes and Genomes (KEGG) pathway (**E**) and Gene Ontology (GO) biological process (**F**) enrichment analysis of the DEGs.

To further elucidate the biological function of RBM41 in TSCC, homozygous RBM41-knockout (RBM41-KO) cells were generated using the CRISPR-Cas9 system (Fig. 3A, B). A total of 1666 significantly dysregulated genes were identified, of which 1107 genes were upregulated and 559 genes were downregulated in the RBM41-KO cells compared to the WT cells (Fig. 3C). As shown in the heatmap in Fig. 3D, the RBM41 KO and WT samples were distinct, indicating that the loss of RBM41 induced significant changes in the transcriptional landscape. Furthermore, the Kyoto Encyclopedia of Genes and Genomes (KEGG) and gene ontology (GO) analyses of the DEGs showed significant enrichment of terms related to metabolic processes. In the KEGG enrichment analysis, the most significant differences were observed in the “Ribosome” category under Genetic Information Processing, followed by “sulfur metabolism” and “Alanine, aspartate and glutamate metabolism” classified under Metabolism (Fig. 3E). In addition, the significantly enriched GO terms were primarily related to processes such as “RNA binding”, “RNA degradation”, and “ATP hydrolysis activity” (Fig. 3F). These findings suggest that RBM41 is a key regulatory factor in the metabolic homeostasis of TSCC cells. Deletion of the RBM41 gene disrupted the metabolism of TSCC cells, which may be related to the direct binding of RBM41 to key metabolic mRNAs and their possible stabilization.

### RBM41 protects TSCC cells from disulfidptosis under glucose deprivation

**Fig. 4:**
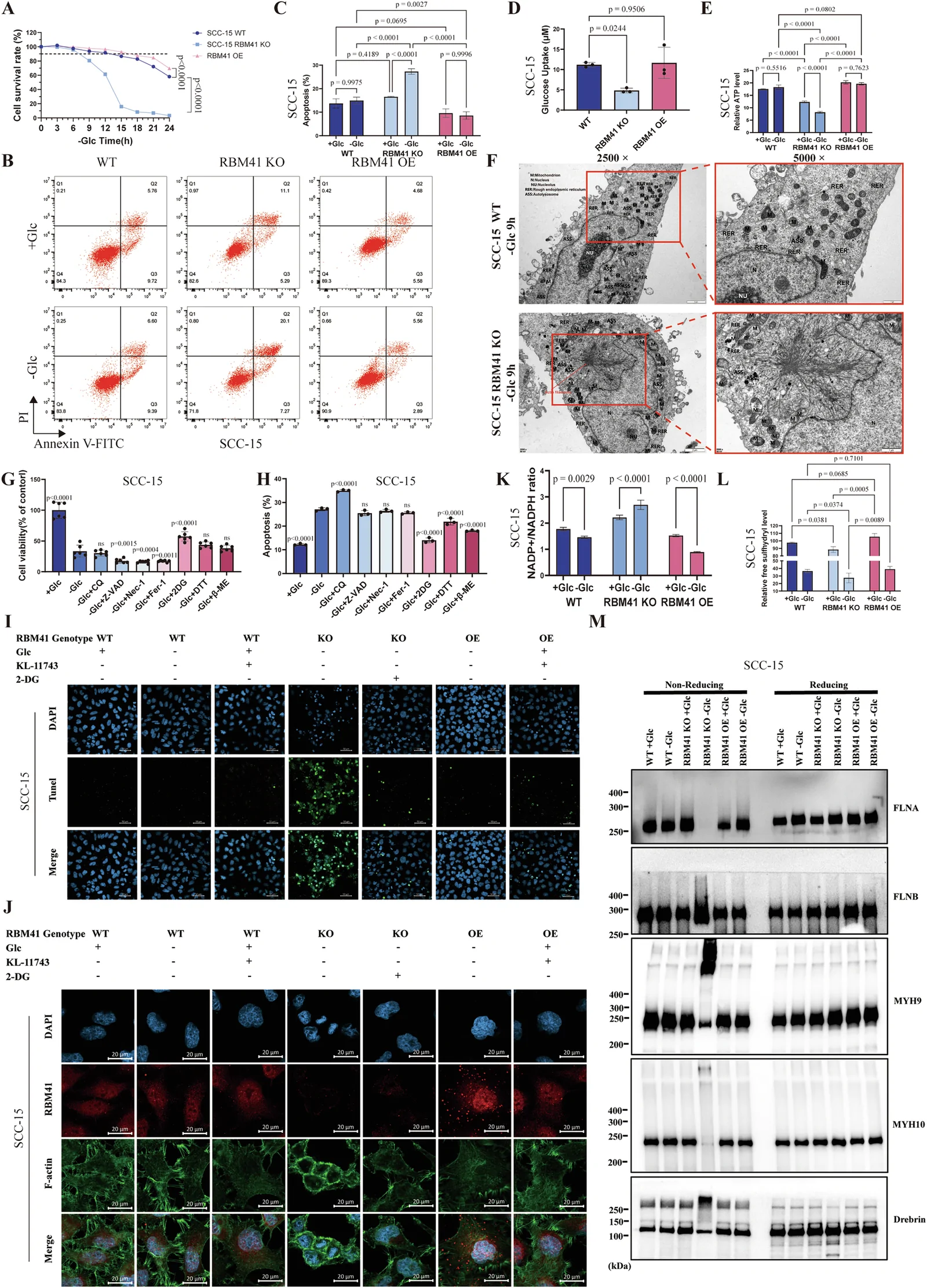
RBM41 deficiency sensitizes TSCC cells to disulfidptosis under glucose deprivation. **A** CCK-8 assay measuring cell viability of SCC-15 cells under glucose deprivation (-Glc) over time, *n* = 6 biological replicates. Flow cytometry analysis (**B**) and quantification (**C**) of cell death after 9 h of -Glc treatment. **D** Glucose uptake assay in SCC-15 cells. **E** Intracellular ATP levels in SCC-15 cells under normal and -Glc conditions. **F** TEM images of SCC-15 cells under -Glc 9 h, showing plasma membrane rupture and actin filament contraction. Scale bar = 2 μm. Cell viability and mortality of RBM41 KO cells treated with -Glc and various cell death inhibitors for 9 h were measured using the CCK-8 assay (**G**) and flow cytometry analysis (**H**). CCK-8 assay, *n* = 6 biological replicates; flow cytometry analysis, *n* = 3 biological replicates. **I** TUNEL staining (green) of RBM41 KO cells under -Glc with or without 2-DG pre-treatment. Nuclei are counterstained with DAPI (blue). Scale bar = 50 μm. **J** Immunofluorescence staining showing RBM41 (red), F-actin (green), and nuclei (blue) in WT and RBM41-KO cells under -Glc 9 h. Scale bar = 20 μm. **K** NADP+/NADPH ratio in SCC-15 cells under -Glc 4 h. **L** Intracellular free sulfhydryl levels measured by Ellman’s reagent in SCC-15 cells under -Glc 9 h. **M** Non-reducing Western blot analysis (left) and reducing Western blot analysis (right) of key cytoskeletal proteins in SCC-15 cells under -Glc 9 h. Error bars represent mean ± SD, *n* = 3 biological replicates. Significance was determined using a one-way ANOVA with Dunnett’s multiple comparisons test (**D**, **E**, **G**, **H**), two-way ANOVA with Šídák’s multiple comparisons test (**K**), or two-way ANOVA with Tukey’s multiple comparisons test (**A**, **C**, **L**). *P* values are as indicated.

To determine whether RBM41 protects cancer cells during metabolic stress, the WT, RBM41-KO, and RBM41-overexpressing (RBM41-OE, transfected and overexpressed in wild-type SCC-9 or SCC-15 cells) TSCC cells were subjected to glucose deprivation (-Glc), glutamine deprivation (-Gln), and serum deprivation (-FBS) treatments. The lack of glucose led to a time-dependent decrease in SCC-15 cell viability, and the death rate of RBM41-KO cells was significantly accelerated compared to that of WT and RBM41-OE cells (Fig. 4A). The same result was also observed in SCC-9 cells (Supplementary Fig. 3A). Consistent with this, a significantly higher percentage of dead cells was observed in the RBM41-KO group after 9 h of exposure to -Glc conditions (Fig. 4B, C, Supplementary Fig. 3B, C). However, RBM41 KO did not exhibit a stronger cell death effect under -FBS and -Gln conditions (Supplementary Fig. 4). Therefore, we speculate that the loss of RBM41 specifically enhances the sensitivity of TSCC cells to cell death induced by glucose deprivation.

Furthermore, the RBM41-KO cells exhibited a reduced capacity for glucose uptake (Fig. 4D and Supplementary Fig. 5A), along with a significant reduction in intracellular ATP levels under both normal and -Glc conditions (Fig. 4E and Supplementary Fig. 5B). Interestingly, cystine uptake remained unchanged across all groups, regardless of glucose availability (Supplementary Fig. 6). Transmission electron microscopy (TEM) analysis of RBM41-KO cells cultured under -Glc conditions revealed significant plasma membrane rupture and contraction of actin filaments (Fig. 4F), which coincided with the ultrastructural changes associated with disulfidptosis. To confirm the nature of cell death, the RBM41-KO cells were treated with pharmacological inhibitors targeting the major death pathways. The elevated mortality rate of the RBM41-KO cells could not be rescued by inhibiting the apoptosis (Z-VAD), necroptosis (Nec-1), ferroptosis (Fer-1), or autophagy (chloroquine, CQ) pathways. In contrast, the glycolysis inhibitor 2-deoxy-D-glucose (2-DG) and the reducing agents dithiothreitol (DTT) and beta-mercaptoethanol (β-ME) significantly alleviated cell death (Fig. 4G, H and Supplementary Fig. 7). Given that KL-11743, a competitive inhibitor of class I GLUTs, is capable of inducing disulfidptosis, the above finding was strongly suggestive of cell death induced by disulfide bond stress. Consistent with this, the intense green fluorescence observed in the stressed RBM41-KO cells in the TUNEL assay was effectively suppressed by pre-treatment with 2-DG (Fig. 4I). In addition, the RBM41-KO cells exhibited a severely disrupted actin network—a characteristic manifestation of disulfidptosis—under glucose deprivation, which was not observed in WT cells (Fig. 4J). Furthermore, the NADP+/NADPH ratio was also elevated in the RBM41-KO cells (Fig. 4K and Supplementary Fig. 8A), indicating redox imbalance and a reduced capacity to alleviate oxidative stress. The imbalance in redox capacity resulted in corresponding alterations in cellular ROS levels (Supplementary Fig. 8B, C) and the dysregulation of the glutathione (GSH) to oxidized glutathione (GSSG) ratio (Supplementary Fig. 9). Disulfidptosis is triggered by the formation of aberrant disulfide bonds within actin proteins, leading to their aggregation and subsequent cytoskeletal collapse. To directly evaluate intracellular disulfide bond formation, the levels of free sulfhydryl groups were measured using Ellman’s reagent. The RBM41-KO cells showed a significant reduction in free sulfhydryl groups compared to the WT cells under -Glc conditions (Fig. 4L), indicating widespread abnormal oxidation and disulfide bond formation. The protein extracts from RBM41-KO cells cultured under -Glc conditions displayed significant high molecular weight tails and aggregation, whereas no such phenomena were observed for the WT or RBM41-OE cells. Furthermore, the aggregates completely disappeared upon addition of the reducing agent DTT to the sample buffer (Fig. 4M). This finding provided direct biochemical evidence for the excessive disulfide bond-mediated crosslinking of cytoskeletal proteins in RBM41-deficient cells under metabolic stress.

High levels of SLC7A11 expression and glucose deprivation are indeed two indispensable prerequisites for disulfidptosis. We confirmed through Western blot analysis that TSCC exhibited high levels of SLC7A11 protein expression compared to 293T, HOK, and HOEC cells with lower SLC7A11 levels, providing a prerequisite for the occurrence of disulfidptosis in TSCC (Supplementary Fig. 10A, B). To further investigate the potential regulatory relationship between RBM41 and SLC7A11, SLC7A11 knockout cells (SLC7A11 KO) and RBM41 KO cells with simultaneous SLC7A11 KO (RBM41 and SLC7A11 double knockout, R&S DKO) cell lines were constructed (Supplementary Fig. 10C, D). We found that RBM41 knockout did not alter the mRNA or protein levels of SLC7A11 (Supplementary Fig. 10E–G). As shown in Supplementary Fig. 10H–J, glucose deprivation induced significant cell death in RBM41 KO cells, which could be temporarily alleviated by the knockout of SLC7A11. This indicates that RBM41 regulates disulfidptosis through a unique pathway rather than by directly modulating the expression of SLC7A11.

### The RRM 1 domain of RBM41 is essential for its RNA-binding and anti-disulfidptosis function

**Fig. 5:**
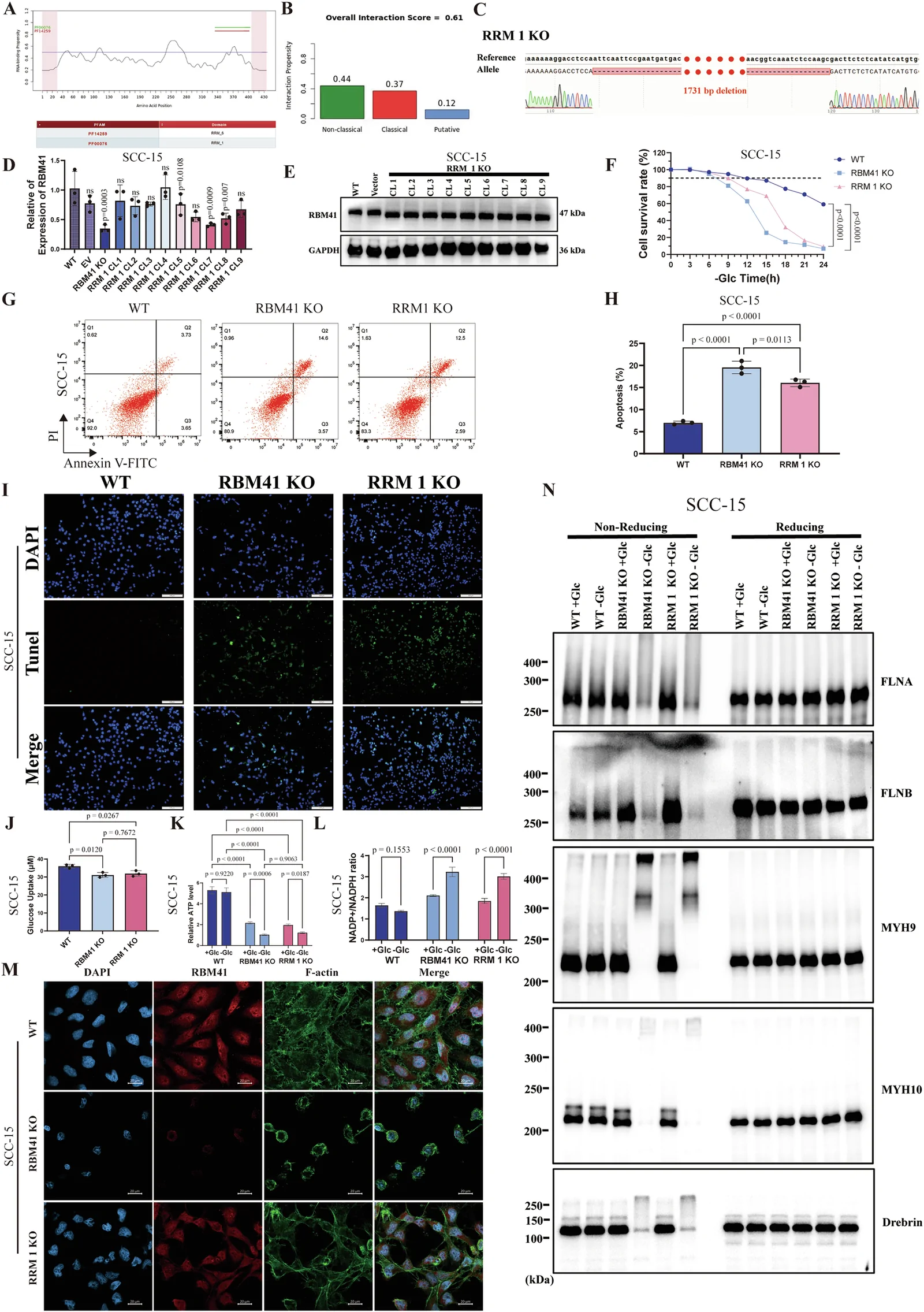
The RRM 1 domain is essential for RBM41’s function in protecting against disulfidptosis. **A** The domain architecture of the RBM41 protein is illustrated, with the RRM 1 domain represented by green lines and the RRM 6 domains represented by red lines. **B** catRAPID signature prediction of RNA-binding propensity for RBM41 domains. **C** Schematic of the CRISPR/Cas9 strategy for targeted deletion of the RRM 1 domain genomic region and the associated Sanger sequencing chromatograms. qRT-PCR (**D**) and Western blot **E** analysis of truncated RBM41 transcript and protein expression in selected RRM 1-KO clones, *n* = 3 biological replicates. **F** CCK-8 assay measuring cell viability of SCC-15 cells under -Glc over time, *n* = 6 biological replicates. Flow cytometry analysis (**G**) and quantification (**H**) cell death in SCC-15 cells after 9 h of -Glc, *n* = 3 biological replicates. **I** TUNEL staining (green) of SCC-15 cells after 9 h of -Glc. Nuclei are counterstained with DAPI (blue). Scale bar = 100 μm. **J** Glucose uptake assay in SCC-15 cells, *n* = 3 biological replicates. **K** Intracellular ATP levels in SCC-15 cells, *n* = 3 biological replicates. **L** NADP + /NADPH ratio in SCC-15 cells, *n* = 3 biological replicates. **M** Immunofluorescence staining showing RBM41 (red), F-actin (green) and nuclei (blue) in SCC-15 cells under -Glc. Scale bar = 20 μm. **N** Non-reducing Western blot analysis (left) and reducing Western blot analysis (right) of key cytoskeletal proteins in SCC-15 cells under -Glc 9 h. Error bars represent mean ± SD. Significance was determined using a one-way ANOVA with Dunnett’s multiple comparisons test (**D**), one-way ANOVA with Tukey’s multiple comparisons test (**H**, **J**), two-way ANOVA with Dunnett’s multiple comparisons test (**F**) or two-way ANOVA with Tukey’s multiple comparisons test (**K**, **L**). *P* values are as indicated.

The RNA-binding domains of RBM41 were predicted using the catRAPID signature algorithm, which revealed two potential regions: the RRM 1 domain (PFAM: PF00076) and the RRM 6 domain (PFAM: PF14259) (Fig. 5A). The overall interaction propensity score was calculated to be 0.61, and the RRM 1 domain was predicted to be the primary contributor to RNA binding (Fig. 5B and Supplementary Fig. 11). To experimentally validate the in silico prediction, the genomic region encoding the RRM 1 domain was deleted using the CRISPR-Cas9 system and specific sgRNAs, while preserving the overall structural integrity of the remaining RBM41 protein. Using this approach, a series of RRM1 domain knockout (RRM1-KO) SCC-15 cell clones were generated (Fig. 5C). The obtained surviving clones stably expressed both truncated RBM41 transcripts and protein, indicating that the truncated mRNA was translated into a stable protein lacking the RRM1 domain (Fig. 5D, E). The gene-edited cells demonstrated an early occurrence of stop codons, which suggests the production of a mutant protein characterized by a deletion in the RRM1 domain (Supplementary Figs. 12 and 13).

The RRM 1-KO cells (represented by clone #9) showed increased mortality under -Glc conditions in a time-dependent manner, which was consistent with the behavior of RBM41-KO cells (Fig. 5F and Supplementary Fig. 14A). Likewise, a significant increase in cell death was observed in the RRM 1-KO group compared to the WT control group after 9 h of -Glc stress (Fig. 5G-I and Supplementary Fig. 14B, C). The RRM 1-KO cells also exhibited complete metabolic deficiency characterized by severely impaired glucose uptake (Fig. 5J and Supplementary Fig. 15A) and a significant reduction in intracellular ATP levels (Fig. 5K and Supplementary Fig. 15B). Furthermore, the redox balance was disrupted in these cells, as indicated by an elevated NADP+/NADPH ratio (Fig. 5L and Supplementary Fig. 15C). In addition to a severely disordered actin network (Fig. 5M), the glucose-deprived RRM 1-KO cells also showed significant disulfide bond-mediated aggregation of cytoskeletal proteins (Fig. 5N). Taken together, targeted deletion of the RRM1 domain is sufficient to mimic the metabolic dysfunction and disulfidptosis sensitivity observed in RBM41-KO cells, indicating that the RRM1 domain is indispensable for the function of RBM41.

### RBM41 binds to and stabilizes CKMT1B mRNA to maintain the cellular energy shuttle

**Fig. 6:**
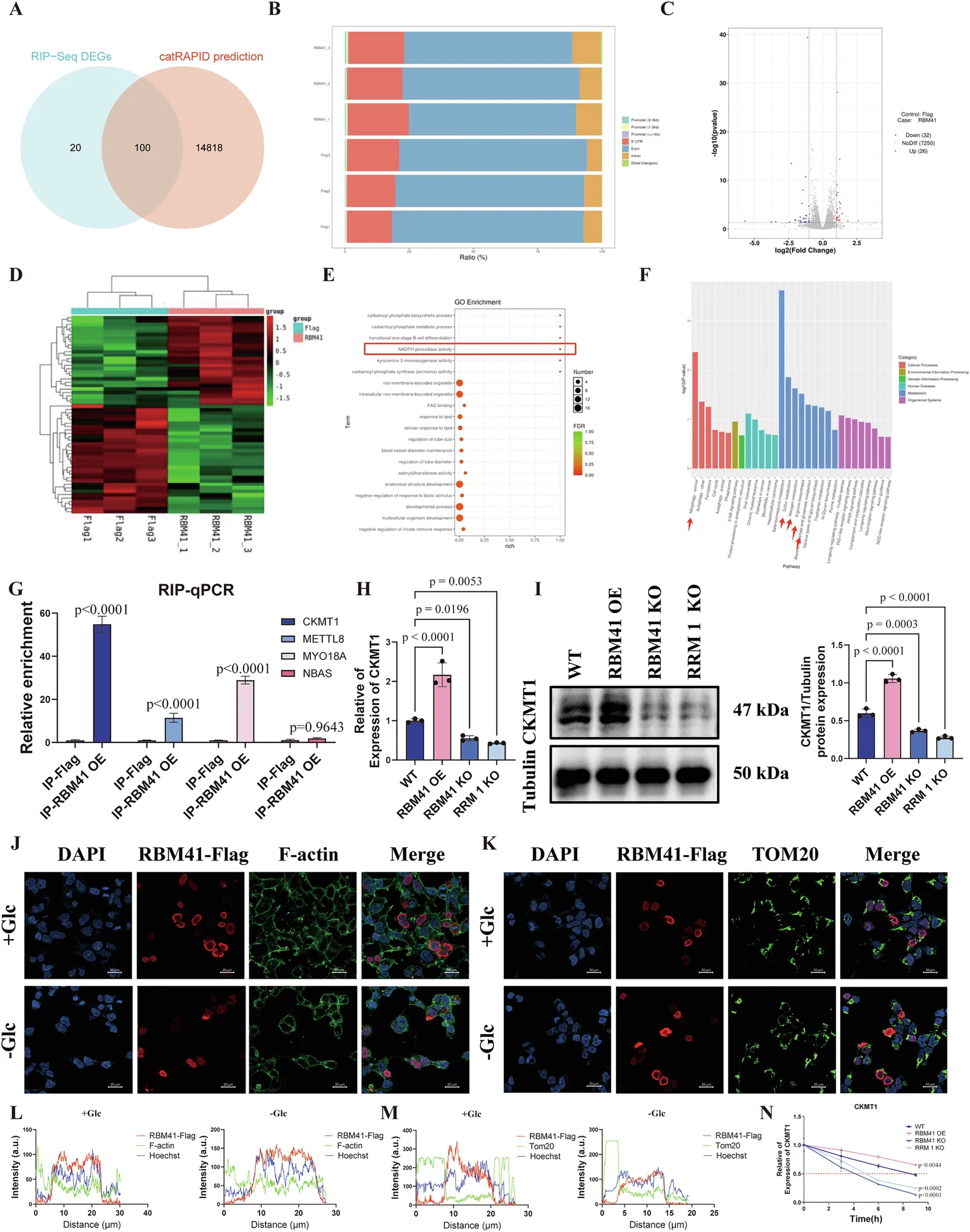
RBM41 directly binds to and stabilizes CKMT1B mRNA. **A** The Venn diagram illustrates the intersection of differentially expressed genes (DEGs) identified by RIP-Seq (blue) and genes predicted by catRAPID (red). **B** The enrichment of RBM41 in different introns, exons, and various functional regions of organisms. **C** Volcano plot of mRNAs significantly enriched in RBM41-Flag RIP-Seq. **D** Heatmap of mRNAs significantly enriched in RBM41-Flag RIP-Seq. GO biological process **E** and KEGG pathway **F** enrichment analysis of RBM41-bound mRNAs. **G** RIP-qPCR validation of the enrichment of candidate target mRNAs (METTL8, CKMT1, NABS, MYO18A) in RBM41-Flag immunoprecipitates, *n* = 3 biological replicates. qRT-PCR **H** and Western blot **I** analysis of CKMT1 expression in WT, RBM41 OE, RBM41 KO, and RRM 1 KO cells, *n* = 3 biological replicates. **J** Immunofluorescence staining showing the subcellular localization of RBM41-Flag (red) relative to the nucleus (DAPI, blue), and cytoplasm (F-actin, green) under normal and -Glc conditions. Scale bar = 20 μm. **K** Immunofluorescence staining showing the subcellular localization of RBM41-Flag (red) relative to the nucleus (DAPI, blue) and mitochondria (Tom20, green) under normal and -Glc conditions. Scale bar = 20 μm. **L, M** The fluorescence intensity distribution curve, with the *X*-axis representing distance (µm) and the *Y*-axis representing normalized fluorescence intensity (A.U.). **N** CKMT1B mRNA stability assay. Cells were treated with Actinomycin D, and the relative remaining CKMT1B mRNA levels were measured by qRT-PCR over time, *n* = 3 biological replicates. Error bars represent mean ± SD. Significance was determined using a one-way ANOVA with Dunnett’s multiple comparisons test **H** two-way ANOVA with Dunnett’s multiple comparisons test **N** or two-way ANOVA with Šídák’s multiple comparisons test **G**. *P* values are as indicated.

The catRAPID omics tool was used to predict the RNA-interacting partners of RBM41 protein based on the RIP-Seq data, and the results indicated that the RBM41 protein is more likely to be enriched in the RNA exon regions (Fig. 6A, B). A total of 26 mRNAs were enriched in the RBM41-Flag group compared to the control (Fig. 6C, D). Furthermore, significant enrichment of bound transcripts involved in critical biological processes like sulfur metabolism, NADPH peroxidase activity, and mitophagy (Fig. 6E, F), which aligns with the metabolic phenotype of the RBM41-KO cells. Furthermore, CKMT1B mRNA showed the highest specificity and enrichment ratio among the candidates tested (Fig. 6G), indicating that it is likely the primary direct binding target of RBM41. Survival analysis results based on the TCGA indicate that patients in the high expression group of CKMT1B have a mortality risk 1.26 times that of the low expression group, with a 95% confidence interval of 0.92–1.75, *P* = 0.154 (Supplementary Fig. 1B). Indeed, CKMT1 transcript and protein levels were both significantly downregulated in the RBM41-KO cells, as well as in the RRM1-KO cells (Fig. 6H, I). Thus, loss of RBM41 or its RNA-binding capacity inhibited CKMT1 expression, demonstrating a genetic link between the RRM1 domain and the regulation of this key metabolic target. The cellular localization of RBM41 was next determined by immunofluorescence staining. RBM41-Flag was mainly localized in the nucleus regardless of glucose deprivation, while no significant localization was observed in the mitochondria. Based on these findings, RBM41 primarily exerts its effects within the nucleus (Fig. 6J–M). The half-life of CKMT1B mRNA was significantly extended in the RBM41-OE and shortened in both RBM41-KO and RRM1-KO compared to WT (Fig. 6N).

The expression level of CKMT1 was found to be elevated in TSCC (Supplementary Fig. 16). To unequivocally establish CKMT1 as the functional executor of the RBM41-mediated effect, we generated CKMT1-knockout (CKMT1-KO) SCC-15 cell lines utilizing the CRISPR-Cas9 system. Sanger sequencing confirmed a homozygous 8-bp deletion in the coding sequence of CKMT1, leading to a frameshift mutation (Supplementary Fig. 17A, B). qRT-PCR and Western blot analyses revealed a significant reduction in CKMT1 mRNA and protein levels in the CKMT1-KO cells compared to WT (Supplementary Fig. 17C–E), thereby confirming the successful knockout of the gene. We next assessed the sensitivity of CKMT1-KO cells to glucose deprivation. Flow cytometry analysis revealed that CKMT1-KO cells exhibited significantly elevated rates of cell death under -Glc compared to WT cells, mirroring the vulnerability observed in RBM41-KO cells. Notably, this increased cell death was effectively rescued by 2-DG, DTT, and β-ME (Supplementary Fig. 17F, G). Consistent with the metabolic defects observed in RBM41-KO cells, the ablation of CKMT1 resulted in a significant depletion of intracellular ATP levels (Supplementary Fig. 17H) and an elevated NADP⁺/NADPH ratio (Supplementary Fig. 17I), indicating a compromised energy state and disrupted redox homeostasis. Furthermore, non-reducing SDS-PAGE analysis revealed the formation of high-molecular-weight aggregates of cytoskeletal proteins, including Drebrin, FLNA, FLNB, MYH9, and MYH10, in CKMT1-KO cells subjected to -Glc (Supplementary Fig. 17J).

In summary, these results demonstrate that RBM41 directly binds to and stabilizes CKMT1B mRNA through its RRM1 domain, and the loss of RBM41 or its RNA-binding function can deplete the CKMT1 protein through destabilization of CKMT1B mRNA. Furthermore, CKMT1 acts as a downstream effector through which RBM41 exerts its protective function against disulfidptosis in TSCC.

### Loss of RBM41 suppressed tumor growth in vivo through disulfidptosis induction

**Fig. 7:**
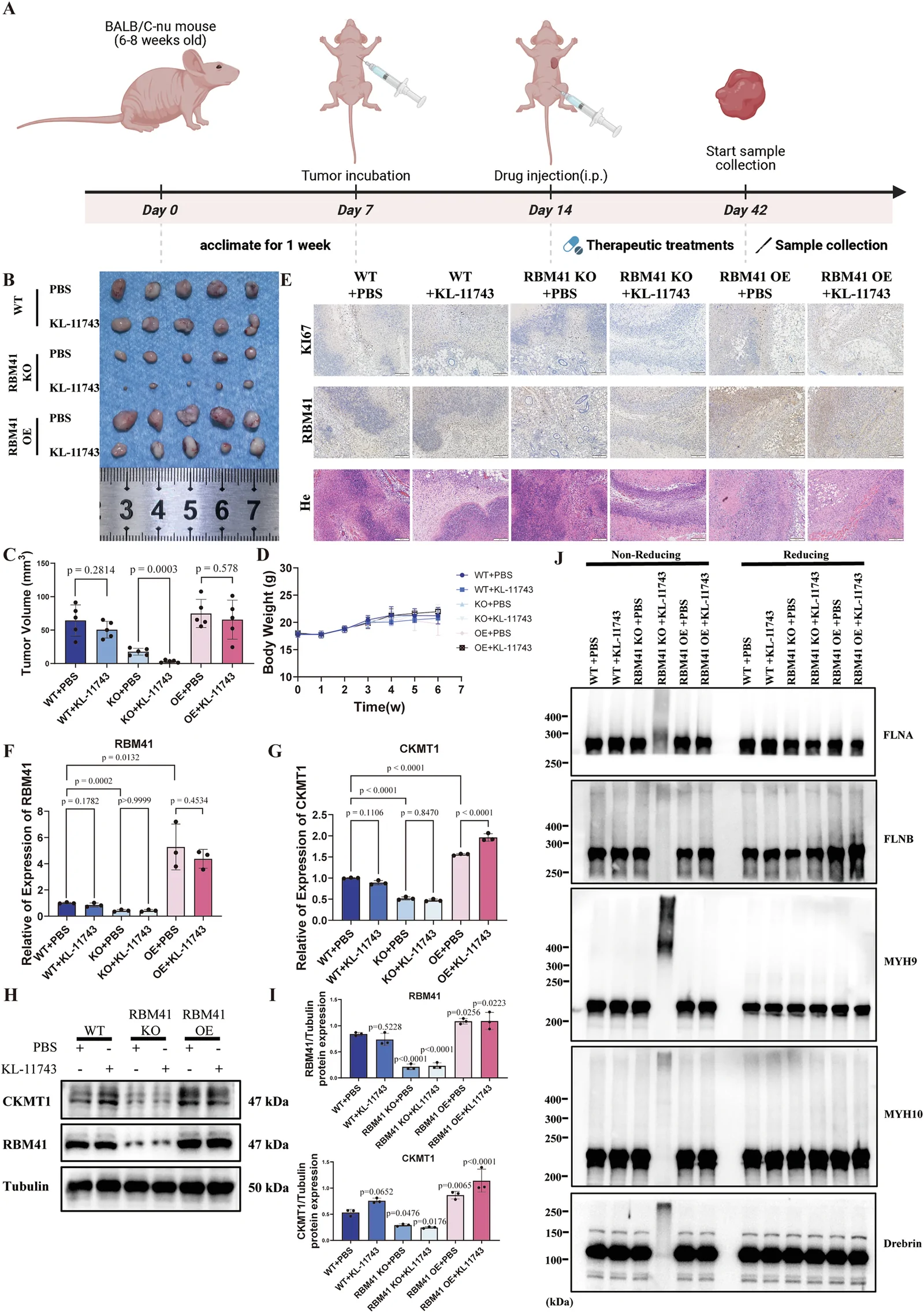
RBM41 suppresses tumor growth by inducing disulfidptosis in vivo. **A** Schematic timeline of the xenograft tumor experiment and drug treatment schedule. Tumor images from xenograft tumors derived from WT, RBM41 KO, and RBM41 OE cells (**B**), final tumor volume measurements **C**, and body weight change curves **D** of these mice treated with PBS or KL-11743, *n* = 5 mice/group. **E** Representative IHC images of RBM41 and Ki-67, and H&E staining of tumor sections from the indicated groups. Scale bar = 100 μm qRT-PCR analysis of RBM41 **F** and CKMT1 **G** expression in the harvested xenograft tumors, *n* = 3 biological replicates. **H, I** Western blot analysis of RBM41 and CKMT1 expression in the harvested xenograft tumors. **J** Non-reducing Western blot analysis (left) and reducing Western blot analysis (right) of key cytoskeletal proteins (Drebrin, FLNA, FLNB, MYH9, MYH10) in xenograft tumor lysates. Error bars represent mean ± SD. Significance was determined using a one-way ANOVA with unpaired *t*-test (**C, F**) or one-way ANOVA with Tukey’s multiple comparisons test **G**. *P* values are as indicated.

To validate the physiological relevance of the findings in an in vivo setting, tumor xenografts were established by subcutaneously inoculating nude mice with WT, RBM41-KO, or RBM41-OE TSCC cells (Fig. 7A). Considering that *RBM41* is located on the X chromosome, the nude mice used in vivo were all female. As expected, tumors derived from RBM41-KO cells exhibited markedly impaired growth compared to the WT tumors in the PBS-treated mice. On the other hand, tumor growth was almost completely suppressed in the RBM41 KO + KL-11743 group (Fig. 7B, C), suggesting a potent synthetic lethal interaction between RBM41 deficiency and KL-11743 treatment. No significant differences in body weight were observed in any of the groups (Fig. 7D), which indicated that the treatment was well tolerated and that the anti-tumor effects were not due to general toxicity. Furthermore, the RBM41-KO tumors showed a significant reduction in the number of Ki-67-positive proliferating cells, which was further exacerbated by KL-11743 treatment. H&E staining of tumor tissues from the RBM41-KO and KL-11743-treated groups revealed areas of cell death with morphological features consistent with in vitro observations (Fig. 7E). Currently, pathological examinations of the submandibular lymph nodes, brain, lungs, and liver from each group of nude mice have not revealed any tumor metastasis (Supplementaryary Fig. 18). The expression levels of RBM41 mRNA and protein in the RBM41-KO and RBM41-OE tumor lysates were also consistent. Furthermore, CKMT1 was significantly downregulated at both mRNA and protein levels in the RBM41-KO xenografts, thereby reinforcing the critical regulatory axis between RBM41 and CKMT1 in vivo (Fig. 7F– I).

Notably, high molecular weight aggregates of core cytoskeletal proteins were detected exclusively in the tumors of RBM41 KO+ PBS group (Fig. 7J). This finding provided definitive biochemical evidence that the loss of RBM41, particularly in conjunction with KL-11743-induced stress, initiates disulfidptosis within the tumor microenvironment, resulting in tumor growth suppression. In conclusion, RBM41 is essential for TSCC growth, and its knockout sensitizes the tumor cells to disulfidptosis. The synthetic lethality achieved by combining RBM41 deficiency with KL-11743 treatment highlights a promising novel therapeutic strategy for targeting TSCC tumors with compromised RBM41 function.

### L759-0762 binds to the RRM1 domain of RBM41 to disrupt its interaction with CKMT1 mRNA and induce disulfidptosis

**Fig. 8:**
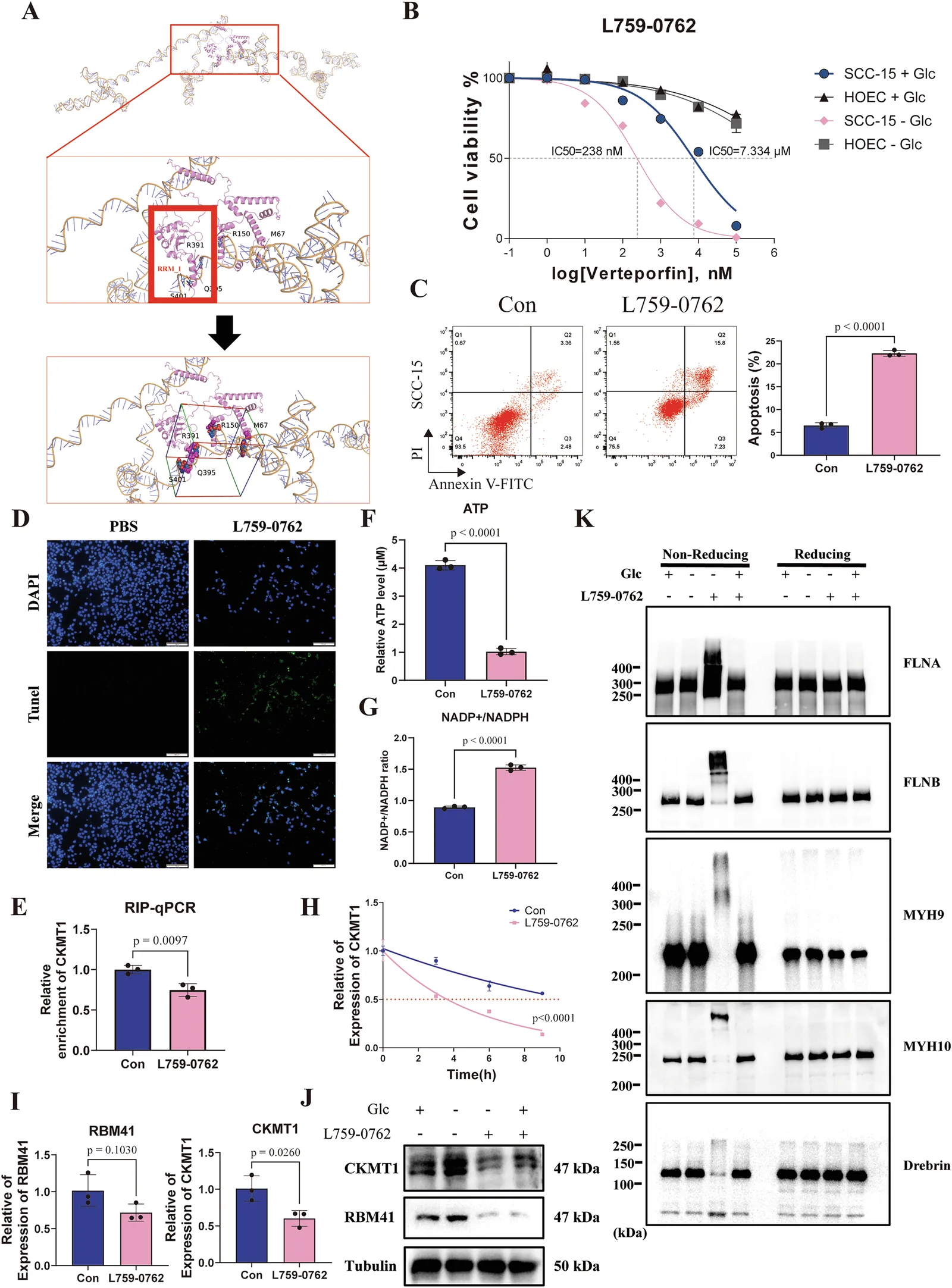
The small molecule L759-0762 targets RBM41 to disrupt the RBM41-CKMT1B axis and induce disulfidptosis. **A** Predicted 3D structure of the RBM41 RRM 1 domain (from AlphaFold) docked with CKMT1B mRNA (from trRosettaRNA), highlighting the predicted drug-binding pocket. **B** L759-0762 and its IC50 values in SCC-15 cells under + Glc and − Glc conditions, and in HOEC cells, *n* = 3 biological replicates. Flow cytometry analysis **C** and TUNEL assay **D** quantifying cell death in SCC-15 cells treated with 5 μM L759-0762 under + Glc for 9 h, *n* = 3 biological replicates. **E** RIP-qPCR analysis showing the enrichment of CKMT1B mRNA in RBM41-Flag immunoprecipitates from cells treated with PBS (Con) or 5 μM L759-0762, *n* = 3 biological replicates. Intracellular ATP levels **F** and NADP+/NADPH ratio **G** in SCC-15 cells treated with 5 μM L759-0762, *n* = 3 biological replicates. **H** CKMT1B mRNA stability assay in SCC-15 cells treated with PBS or 5 μM L759-0762, *n* = 3 biological replicates. qRT-PCR **I** and Western blot **J** analysis of CKMT1 expression in SCC-15 cells treated with PBS or 5 μM L759-0762, *n* = 3 biological replicates. **K** Non-reducing Western blot analysis (left) and reducing Western blot analysis (right) of key cytoskeletal proteins (Drebrin, FLNA, FLNB, MYH9, MYH10) in SCC-15 cells treated with 5 μM L759-0762 under + Glc or − Glc conditions. Error bars represent mean ± SD. Significance was determined using an unpaired *t*-test (**C, E, F, G, I**) or two-way ANOVA with Šídák’s multiple comparisons test **H**. *P* values are as indicated.

The three-dimensional structure of RBM41 and the structure of CKMT1B mRNA (exons 4–10) were modeled (Fig. 8A). Molecular docking of these structures revealed a potential drug-binding pocket in the RRM1 domain (Fig. 8A), which was used for the virtual screening of the ChemDiv compound library through the DrugCLIP platform. Several high-ranking candidate compounds were identified, including L977-1448, L759-0762, and L937-0176 (Table 1), and their efficacy against TSCC cells was evaluated in vitro. L759-0762 exhibited the most potent anti-proliferative activity, and its half-maximal inhibitory concentration (IC50) under − Glc was 238 nM compared to 7.334 μM under + Glc (Fig. 8B), indicating significantly higher cytotoxicity during metabolic stress. In contrast, L977-1448 demonstrated only modest effects, while L937-0176 proved ineffective (Supplementary Fig. 19). In addition, L759-0762 was highly selective against malignant cells, and even a high concentration of 100 μM did not achieve the IC50 dose in the non-malignant HOEC cells (Fig. 8B). The cytotoxic effect of L759-0762 was further validated by the significant increase in apoptosis rates after 9 h of treatment with 5 μM of the drug (Fig. 8C, D). The immunoprecipitates from L759-0762-treated cells showed a significant reduction in the enrichment of CKMT1B mRNA compared to the control cells (Fig. 8E), which provides direct evidence that L759-0762 inhibits the interaction between RBM41 and CKMT1 mRNA. Consistent with these findings, L759-0762 treatment replicated the phenotype associated with RBM41 deficiency (Fig. 8F-K; Supplementary Fig. 20).In conclusion, L759-0762 disrupts the RBM41-CKMT1B mRNA interaction by competitively binding to the RRM 1 domain of RBM41, which results in metabolic catastrophe and selectively induces disulfidptosis in TSCC cells under nutrient stress.

**Table 1:** The top 10 small molecular compounds were selected from the ChemDiv compound library.

Mol ID	Library	DrugCLIP score	Vina score	MW	LogP
L977-1448	ChemDiv	2.08	−8.674	440.48	4.45
L759-0762	ChemDiv	2.05	−8.269	452.49	4.81
L814-0686	ChemDiv	2.08	−8.118	465.92	4.83
L937-0176	ChemDiv	2.12	−8.018	408.85	3.78
L794-0092	ChemDiv	1.98	−7.885	423.54	3.59
L937-0160	ChemDiv	2.03	−7.703	418.46	3.44
L849-0636	ChemDiv	2.00	−7.667	499.00	4.28
J023-0364	ChemDiv	1.98	−7.643	472.93	4.23
L937-0180	ChemDiv	2.02	−7.557	468.90	3.79
L935-0158	ChemDiv	2.06	−7.434	404.45	3.97

The top 10 small molecular compounds.

## Discussion

Despite multimodal therapy, advanced-stage disease commonly exhibits drug resistance and recurrence, leading to poor survival outcomes [11]. The substantial toxicity and multidrug resistance of conventional chemotherapy highlight the critical need for novel targeted therapies with improved efficacy and safety profiles [12–14]. RBPs represent a promising yet largely untapped reservoir of potential therapeutic targets. As master regulators of post-transcriptional modifications, RBPs control multiple oncogenic processes, and targeting a specific RBP can disrupt downstream oncogenic pathways, thereby presenting a robust strategy for cancer treatment [15]. In this study, the RBM41-disulfidptosis axis was identified as a promising new therapeutic target for TSCC.

Several oncogenic RBPs have been identified in recent years, such as HuR/ELAVL1 that upregulates oncogenes like VEGF and cyclins by binding to and stabilizing their mRNAs [16, 17]. RBPs such as SRSF1 and hnRNP A1 are overexpressed in cancer cells and activate key oncogenes through abnormal alternative splicing [18, 19]. Furthermore, NANOS1 interacts with the TGF-β receptor 1 (TGFBR1) mRNA in OSCC cells, leading to accelerated degradation of the transcript while simultaneously downregulating the TGFBR1 protein, which eventually blocks the downstream TGF-B/SMAD signaling pathway [20]. RBPs have also been associated with the malignant progression of TSCC. For instance, IGF2BP2 stabilizes EGFR and PIK3R1 mRNAs through m6A-dependent interactions, and maintains the EGFR/PI3K/AKT oncogenic axis in an activated state [21]. In this study, CKMT1B mRNA as a critical target of RBM41 was identified. The loss of RBM41 destabilized CKMT1B mRNA and triggered a bioenergetic crisis in TSCC cells.

CKMT1, a key enzyme involved in the phosphocreatine energy shuttle, is located in the mitochondrial intermembrane space and facilitates ATP transfer from the mitochondria to cytoplasmic regions with high energy consumption [22, 23]. CKMT1 inhibition altered mitochondrial respiration and ATP production, and this effect was neutralized by phosphocreatine-mediated reactivation of the arginine-creatine pathway [24]. Therefore, it is reasonable to speculate that the destabilization of CKMT1B mRNA due to the absence of RBM41 directly disrupts this core bioenergetic infrastructure, leading to ATP depletion and an energy crisis. The compromised energy state inevitably exerts pressure on the NADPH system, as NADPH regeneration is an ATP-dependent process [25]. Consistent with this, we observed an increase in the NADP+/NADPH ratio in the RBM41-KO cells, which was accompanied by excessive disulfide bond formation due to the loss in reducing capacity. Although the RBM41-CKMT1B axis differs from the established SLC7A11-mediated disulfidptosis pathway, the two are functionally interconnected. In the SLC7A11 pathway, cystine uptake directly leads to the accumulation of disulfide bonds under conditions of glucose deprivation [5]. TSCC cells typically express high levels of SLC7A11, which provides a prerequisite for the induction of disulfidptosis [26]. On the other hand, the loss of RBM41 and the consequent downregulation of CKMT1B lead to energy depletion but do not hinder the ability to take up cystine. This also results in a metabolic imbalance of cystine under conditions of energy deficiency, ultimately leading to abnormal cytoskeletal contraction and membrane detachment [5, 27]. The involvement of the RBM41-CKMT1B axis in inducing disulfidptosis suggests that the expression level of RBM41 may predict the sensitivity of TSCC patients to disulfidptosis-inducing drugs.

Direct gene editing therapies targeting tumor cells are fraught with various challenges. In addition, the current gene editing technologies in cancer treatment are primarily focused on tumor immunotherapy [28]. Small molecule drugs targeting RBM sites have demonstrated significant therapeutic potential. For instance, Risdiplam, a novel drug for treating spinal muscular atrophy, regulates splicing of SMN2 pre-mRNA by binding to the splicing factor and its surrounding protein complexes [29]. Furthermore, inhibition of RBM39 using arylsulfonamide drugs is a promising strategy for targeting cancer cells expressing high levels of the adaptor protein DCAF15 [30]. Structural prediction and drug target design for intrinsically disordered proteins and peptides have become feasible in recent years with the incorporation of artificial intelligence (AI), which may provide support for designing RBPs-targeting drugs as well [31, 32]. An RBP may contain multiple RNA binding domains, such as the RRM, the K-homology (KH) domain, and the DEAD-box domain, which function in a concerted manner [33]. Therefore, inhibition of a single domain may not be sufficient to completely inactivate the protein, as other domains may retain weak binding capabilities [34]. This study identified the compound L759-0762 using the RNA-binding groove predicted in RRM 1 as the target. L759-0762 mimicked the functional effects of RRM1 knockout by effectively disrupting the RBM41-CKMT1B interaction, depleting ATP, and sensitizing the TSCC cells to disulfidptosis, thus confirming that the RRM1 structural domain is a druggable target. The significant synthetic lethality observed between L759-0762 and glucose deprivation, along with the compound’s marked selectivity towards cancer cells, underscores the therapeutic potential of this approach.

## Materials and methods

### Cell culture

HEK293, Cal-27, SCC-9, and SCC-15 cell lines were purchased from American Type Culture Collection (ATCC). The HOK and HOEC cell lines were purchased from the Shanghai Cell Resource Center in China. HEK293, Cal-27, HOK, HOEC and SCC-15 cell lines were maintained in a humidified atmosphere of 37 °C in a 5% CO_2_ and cultured in Dulbecco’s Modified Eagle’s Medium (MeilunBio, cat. # MA0212) Supplemented with 10% fetal bovine serum (FBS, Thermo Fisher Gibco, cat. # A56)9701), penicillin and streptomycin (1% vol, MeilunBio, cat. # MA0110). SCC-9 cell lines were maintained in a humidified atmosphere at 37 °C in 5% CO_2_ and cultured in Dulbecco’s Modified Eagle’s Medium/F12 (MeilunBio, cat. # MA0214) supplemented with 10% FBS, penicillin, and streptomycin (1% vol).

### Bioinformatic analysis

The RNA-seq expression data (HTSeq-FPKM) and corresponding clinical information for Oral squamous cell carcinoma (OSCC) were downloaded from The Cancer Genome Atlas (TCGA) data portal (https://portal.gdc.cancer.gov/). The dataset included 362 tumor tissues and 32 matched normal adjacent tissues. Differential expression analysis of 124 curated RBM genes was performed using the DESeq2 package (version 1.38.3) in R (version 4.2.2). Genes with an adjusted *p*-value (Benjamini-Hochberg procedure) of < 0.05 and an absolute log2 fold change (|log2FC|) > 1 were considered significantly dysregulated.

The three-dimensional (3D) protein structure of human RBM41 was retrieved from the AlphaFold Protein Structure Database (https://alphafold.ebi.ac.uk/; UniProt: Q96IZ5, AF-Q96IZ5-F1-v4). The 3D structure of the CKMT1B mRNA (transcript ID: ENST00000300283), encompassing exons 4 to 10, was predicted de novo using trRosettaRNA (https://yanglab.qd.sdu.edu.cn/trRosettaRNA/). Molecular docking between the RBM41 protein and the CKMT1B mRNA was performed using HDOCK (http://hdock.phys.hust.edu.cn/) to identify potential binding interfaces and pockets. The top-ranked docking pose based on the HDOCK score was selected for subsequent analysis. The database used for predicting the binding capacity of RBM41 protein is catRAPID signature (http://service.tartaglialab.com/page/catrapid_group).

The predicted ligand-binding pocket on the RRM 1 domain of RBM41 was used as the target for structure-based virtual screening. Screening of the ChemDiv compound library (approximately 1.6 million molecules) was performed using the DrugCLIP platform (https://www.drugclip.com/index). The screening workflow integrated molecular docking and pharmacophore modeling to prioritize compounds with high predicted binding affinity and complementarity to the target pocket. The top 10 ranked compounds were selected for further evaluation.

### Plasmid construction, siRNA synthesis, and transfection

The plasmid for human RBM41 overexpression, pRP-mCherry/Neo-CAG>hRBM41(NM_001324243.1), was purchased from VectorBuilder (Guangzhou, China). To generate the RBM41-3×Flag fusion construct, a DNA fragment encoding a 3×Flag tag was inserted in-frame at the C-terminus of the RBM41 coding sequence in the aforementioned plasmid using standard molecular cloning techniques.

For the CRISPR/Cas9-mediated knockout, single-guide RNAs (sgRNAs) targeting the exon encoding the RRM1 domain of RBM41 or the full gene were designed using the CRISPRREGN Tools (http://www.rgenome.net/cas-designer/). The oligonucleotides encoding the sgRNAs were cloned into the BbsI restriction site of the PX459 vector (Addgene, cat. # 171370). The sequences of the sgRNAs used in this study are as follows (Supplementary Table 1).

All small interfering RNAs (siRNAs) used in this study, including the negative control siRNA (siNC) and RBM41-targeting siRNA (siRBM41), were designed and synthesized by GenePharma (Shanghai, China). The siRNA sequences are as follows (Supplementary Table 2).

Plasmid transfections were performed using Lipofectamine 2000 (Thermo Fisher Gibco, cat. # 11668019) according to the manufacturer’s instructions. For siRNA transfections, cells were seeded in appropriate culture dishes and transfected at 50–60% confluence using Lipofectamine 2000 at a working concentration of 1:100 (v:v, reagent:serum-free medium), with a final siRNA concentration of 50 nM. The culture medium was replaced with fresh complete medium 6–8 h post-transfection. Stable cell lines were selected and maintained using appropriate antibiotics, such as Neomycin (G418, MeilunBio, cat. # MA0321) or Puromycin (MeilunBio, cat. # MA0318), at predetermined optimal concentrations.

### RNA extraction and quantitative real-time PCR

Total RNA was isolated from cultured cells or homogenized tissue samples using TRIzol™ Reagent (Thermo Fisher Gibco, cat. # 15596026CN) according to the manufacturer’s instructions. For cDNA synthesis, 1 µg of total RNA was reverse-transcribed using the UnionScript First-strand cDNA Synthesis Mix for qPCR (Genesand, cat. # SR511) with random hexamer primers, following the provided protocol. Quantitative real-time PCR was performed using the GS AntiQ qPCR SYBR Green Fast Mix (Genesand, cat. #SQ410) on a QuantStudio 5 Real-Time PCR System. The relative expression levels of target genes were calculated using the comparative 2^−ΔΔCt^ method and normalized to the endogenous control gene GAPDH or beta-actin. All reactions were performed in technical triplicate. The sequences of the primers used in this study are listed (Supplementary Table 3).

### Protein extraction and western blotting

The protein concentration of the lysates was determined using the BCA Protein Quantification Kit (Abbkine, cat. # KTD3001) according to the manufacturer’s instructions. For protein analysis under reducing conditions, lysates were mixed with 5× Omni-Easy™ Protein Sample Loading Buffer (Epizyme Bio, cat. # LT101S), and then denatured by boiling at 95 °C for 10 min. For the analysis of disulfide bond-mediated protein complexes under non-reducing conditions, lysates were mixed with 5× non-Reducing Protein Sample Loading Buffer (Epizyme Bio, cat. # LT103) without any reducing agents and were not boiled prior to loading to preserve the native protein aggregates.

Equal amounts of protein (typically 10–30 µg) were separated by SDS-PAGE on precast 4-20% PAGE Gel (Epizyme Bio, cat. # PG220) and subsequently transferred onto PVDF membranes (Immobilon, cat. # IPFL00010). The membranes were blocked with 5% (w/v) non-fat dry milk in TBST (Tris-Buffered Saline with 0.1% Tween-20) (Service Bio, cat. # G2150-1L) for 1 h at room temperature and then incubated with primary antibodies diluted in the same blocking solution overnight at 4 °C. The information regarding all primary antibodies involved in the experiment is presented below (Supplementary Table 4). After three washes with TBST, the membranes were incubated with appropriate horseradish peroxidase (HRP)-conjugated secondary antibodies for 1 h at room temperature. The protein bands were imaged using a Western blot imaging system.

### Cell counting kit-8 (CCK-8) assay

Cells were seeded in 96-well plates at a density of 5–7 × 10³ cells per well. After the respective treatments, 10 µL of Cell Counting Kit-8 (DOJINDO, cat. # CK04) was added to each well, followed by incubation at 37 °C for 3 h. The absorbance at 450 nm was measured using a microplate reader. Cell viability was calculated relative to the control group.

### Annexin V/PI apoptosis assay

Cell death was quantified using an Annexin V-FITC/PI Apoptosis Kit (MeilinBio, cat. # MA0220). Briefly, both adherent and floating cells were collected, washed with cold phosphate buffer saline (PBS, MeilinBio, cat. # MA0015), and resuspended in 1× Binding Buffer. The cell suspension was then incubated with 10 µL of Annexin V-FITC and 5 µL of PI for 15 min at room temperature in the dark. The stained cells were analyzed immediately using a BD FACS Celesta flow cytometer, and the data were processed with FlowJo software (v10.8.1).

### TUNEL assay

DNA fragmentation, indicative of cell death, was detected using the TUNEL Assay Kit (Abbkine, cat. # KTA2010). Cells were seeded on glass coverslips placed in 24-well plates at a density of 2 × 10⁴ cells per well. After treatments, cells were fixed with 4% paraformaldehyde fix solution (MeilinBio, cat. # MA0192-1) for 30 min at room temperature and permeabilized with 0.3% Triton X-100 (MeilinBio, cat. # MB2486) for 5 min on ice. The coverslips were then incubated with the TUNEL reaction mixture for 60 min at 37 °C in the dark, according to the manufacturer’s instructions. Cell nuclei were counterstained with DAPI (Thermo Fisher Gibco, cat. # D1306). Images were captured using a fluorescence microscope or laser scanning confocal microscope, and TUNEL-positive (green fluorescence) cells were counted.

### Migration assay

Cells were seeded in 6-well plates and grown to 90–100% confluence. A sterile 200 µL pipette tip was used to create a straight scratch wound across the cell monolayer. The detached cells were gently washed away with PBS, and fresh serum-free medium was added. Images of the same scratched area were captured using an inverted microscope at time points of 0 h, 24 h, 48 h, and 72 h. The migrated area was quantified using ImageJ software (NIH).

### Transwell invasion assay

Cell invasion was assessed using 24-well Transwell chambers with 8.0 µm pore polycarbonate membranes (Corning, cat. # CLS3422). The membranes were pre-coated with 100 µL of Matrigel (1:8 dilution in serum-free medium, Corning, cat. # CLS354234) and allowed to solidify at 37 °C for 4 h. Then, 5 × 10⁴ cells resuspended in 200 µL of serum-free medium were seeded into the upper chamber. The lower chamber was filled with 600 µL of complete medium containing 10% FBS as a chemoattractant. After 48 h of incubation, non-invading cells on the upper surface of the membrane were carefully removed with a cotton swab. The invaded cells on the lower surface were fixed with 4% formaldehyde for 20 min and stained with 0.1% crystal violet for 15 min. The stained cells were imaged and counted in five random fields per well under an inverted microscope.

### mRNA stability assay

The mRNA stability was assessed using the transcriptional inhibitor actinomycin D (MeilinBio, cat. # MB2221). Cells were seeded in 6-well plates at a density of 2 × 10⁵ cells per well and allowed to adhere overnight. The culture medium was then replaced with fresh medium containing 10 μg/mL of actinomycin D to arrest transcription. Total RNA was isolated from the cells at designated time points (0, 3, 6, and 9 h) post-actinomycin D treatment using the TRIzol method as described in “RBM41 knockout may alter the metabolism of TSCC cells via degradation of key metabolic mRNAs”. The isolated RNA was reverse-transcribed into cDNA, and the abundance of the target mRNA (CKMT1B) at each time point was quantified by qRT-PCR, also detailed in “RBM41 knockout may alter the metabolism of TSCC cells via degradation of key metabolic mRNAs”. The CT values at each time point were compared to the mean value at 0 h, and the relative expression levels were calculated using the formula 2^−ΔCT^. The mRNA half-life was calculated from the slope of the best-fit line for the natural logarithm of the remaining mRNA percentage versus time.

### RNA immunoprecipitation (RIP)

RNA immunoprecipitation was performed using the PureBinding RNA Immunoprecipitation Kit (Geneseed, cat. # P0101) according to the manufacturer’s instructions. Briefly, stable TSCC cells expressing RBM41-Flag or empty vector control were lysed in complete RIP lysis buffer supplemented with protease and RNase inhibitors. Cell lysates were incubated with anti-Flag Magnetic Beads pre-washed with RIP wash buffer for 4 h at 4 °C with gentle rotation. The beads were then collected and washed extensively with RIP wash buffer. A small portion (10%) of the total cell lysate was saved as “Input” control for normalization.

RNA bound to the immunopplexes and the Input control were extracted using proteinase K digestion followed by TRIzol reagent. The purified RNA was treated with DNase I to remove genomic DNA contamination.

For RIP-seq, RNA libraries were constructed using the NEBNext® Ultra™ II Directional RNA Library Prep Kit (NEB, cat. # E7765L) following the manufacturer’s recommendations. The final library quality was assessed, and sequencing was performed on an Illumina NovaSeq 6000 platform (PE150) by Personalbio (Shanghai, China).

For RIP-qPCR validation, the purified RNA from RIP experiments was reverse-transcribed into cDNA. The enrichment of specific target mRNAs (including CKMT1B, METTL8, NABS, MYO18A, and RRBP1) in the immunoprecipitates was quantified by qPCR using the methods described in Section 2.3. The relative enrichment was calculated using the comparative ΔΔCt method, normalized to the Input control and expressed as fold-change over the IgG or empty vector control group.

### Glucose uptake assay

Cellular glucose uptake was measured using the Glucose Uptake Assay Kit (WST-8 method) (Beyotime Biotechnology, cat. # S0554S) according to the manufacturer’s instructions. Briefly, cells were seeded in 96-well plates at a density of 1 × 10⁴ cells per well and cultured for 24 h. Prior to the assay, cells were washed twice with PBS and incubated in glucose-free DMEM for 1 h at 37 °C to deplete residual glucose. Add 10 μl of 2-DG (10 mM) and incubate for 20 min. Wash the cells with PBS and add Glucose Uptake Lysis Buffer to fully lyse the cells. Centrifuge and collect the supernatant for subsequent detection. Transfer 50 μl of diluted or undiluted samples into a 96-well plate, adding 50 μl of Glucose Uptake Detection reaction working solution to each well. After incubation at 37 °C for 30 min, the absorbance was measured at 450 nm using a microplate reader. A standard curve was prepared using known concentrations of 2-DG6P (0, 5, 10, 25, 50, 100, 250 and 500 μM) processed in parallel. The glucose uptake was calculated based on the standard curve. All experiments were performed in triplicate.

### ATP level assay

Intracellular ATP levels were quantified using the ATP Assay Kit (Meilunbio, cat. # MA0440) as per the manufacturer’s instructions. Cells were lysed with the provided ATP assay buffer, and the lysates were centrifuged to remove debris. The supernatant was transferred to a 96-well plate and mixed with the ATP reaction mix. The luminescence was measured immediately using a microplate reader. An ATP standard curve was generated for each assay to calculate the absolute ATP concentration in the samples. All experiments were performed in triplicate.

### NADP+/NADPH ratio assay

The NADP+/NADPH ratio was determined using the NADP(H) Assay Kit (Abbkine, cat. # KTB1010). The extracted samples were divided into two aliquots, each containing one million cells: one for measuring NADP+ and the other for measuring NADPH. The cells were collected and extracted using the specified NADP(H) Extraction Buffer. Subsequently, both aliquots were reacted with the working solution and color reagent, and the absorbance at 450 nm was measured at 0 minutes and 30 min. The concentration of NADP+ was calculated by subtracting NADPH from the total NADP(H). The ratio of NADP+/NADPH was directly determined based on these calculated concentrations. The NADP+/NADPH ratio was calculated based on the standard curve. All experiments were performed in triplicate.

### Ellman’s reagent (DTNB) assay

The intracellular level of free sulfhydryl was measured using Ellman’s Reagent (DTNB) according to the manufacturer’s instructions (Beyotime Biotechnology, cat. # S0138S). Briefly, cells from different treatment groups were collected, washed with cold PBS, and lysed in the provided lysis buffer. The lysates were centrifuged at 12,000 × *g* for 10 min at 4 °C to remove insoluble debris. Collect the supernatant and transfer it to a 96-well plate. Mix it with 180 μl of Ellman’s Reagent Solution in the 96-well plate and incubate in the dark at room temperature for 15 min. Measure the absorbance at 412 nm using a microplate reader. Calculate the concentration of free sulfhydryl in the samples using a standard curve generated from known concentrations of GSH Standard. All experiments were performed in triplicate.

### Animal experiments

Six-week-old female BALB/c nude mice were purchased from Liaoning Changsheng Bio-technology Co (Liaoning, China) and housed under specific pathogen-free conditions with a 12-h light/dark cycle, with free access to autoclaved food and water. After one week of acclimatization, the mice were randomly divided into six experimental groups (*n* = 5 per group). Mice were subcutaneously injected in the left flank with 5 × 10⁶ respective TSCC cells suspended in 100 μL of PBS. Tumor growth was monitored every 3 days by measuring the length (*L*) and width (*W*) of the tumors with digital calipers. Tumor volume was calculated using the formula: Volume = (*L* × *W*²) / 2. When the average tumor volume reached approximately 100 mm³, drug treatment was initiated. Mice in the KL-11743 (Selleck, cat. # E0805) treatment groups received intraperitoneal injections of KL-11743 at a dose of 10 mg/kg, prepared in PBS. The control groups received equal volumes of PBS via the same route. Treatments were administered every day for a total of 4 weeks. Mice's body weight was measured concurrently with tumor measurement to monitor treatment-related toxicity. At the experimental endpoint, mice were euthanized by CO₂ asphyxiation. Tumors were carefully excised, weighed, and divided for subsequent analyses: one portion was fixed in 4% paraformaldehyde for 24 h for histopathological examination, and the remaining portions were snap-frozen in liquid nitrogen and stored at −80 °C for molecular biology analyses (qPCR, Western blotting, and non-reducing SDS-PAGE).

### H&E staining and immunohistochemistry

For H&E, paraffin-embedded tissue sections (4–5 μm) were deparaffinized and rehydrated as described above. Sections were stained with hematoxylin for 5 min, differentiated in 1% acid alcohol, and blued in 0.2% ammonia water. Subsequently, sections were counterstained with eosin for 2 min, dehydrated through an ethanol series, cleared in xylene, and mounted with neutral balsam.

For immunohistochemical analysis, tumor tissues were fixed in 4% paraformaldehyde for 24 h, dehydrated through an ethanol series, and embedded in paraffin. Sections (4–5 μm thick) were deparaffinized in xylene and rehydrated through a graded ethanol series. Antigen retrieval was performed by heating the sections in 10 mM sodium citrate buffer (pH 6.0) at 95 °C for 15 min. Endogenous peroxidase activity was quenched by incubation with 3% hydrogen peroxide for 15 min at room temperature. After blocking with 5% normal goat serum for 1 h at room temperature, sections were incubated overnight at 4 °C with primary antibodies against RBM41 (1:200) and Ki-67 (1:500). Following washing with PBS, sections were incubated with HRP-conjugated secondary antibodies for 1 h at room temperature. Signal detection was performed using DAB substrate, and sections were counterstained with hematoxylin.

### Immunofluorescence

Cells were seeded on glass coverslips in 24-well plates. After treatments, cells were fixed with 4% paraformaldehyde for 15 min at room temperature, permeabilized with 0.3% Triton X-100 in PBS for 10 min, and blocked with 5% BSA (Service Bio, cat. # GC305006-100g) for 1 h. Cells were then incubated with primary antibodies against RBM41 (1:200) and Phalloidin (1:200) overnight at 4 °C, followed by incubation with appropriate fluorescently labeled secondary antibodies (Cy3-conjugated anti-rabbit and Alexa Fluor 488-conjugated anti-mouse, 1:1000) for 1 h at room temperature in the dark. Cell nuclei were counterstained with DAPI. Coverslips were mounted onto glass slides, and images were captured using a confocal microscope.

### Transmission electron microscopy

For the ultramicrostructural analysis, TSCC cells were fixed with 2.5% glutaraldehyde at 4 °C for 1 h without washing the culture medium. Subsequently, the cells were quickly scraped off using a cell scraper, allowing them to settle naturally into aggregates, and fixed overnight at 4 °C. After washing with phosphate-buffered saline, the samples were fixed at room temperature with 1% osmium tetroxide for 2 h, followed by dehydration through a series of graded ethanol solutions and embedding in EPON812 resin. Ultrathin sections of 70 nm were prepared using a Leica UC7 ultramicrotome, stained with uranyl acetate and lead citrate, and observed under a Hitachi HT7800 transmission electron microscope operated at 80 kV.

### Cystine uptake assay

Cystine uptake was assessed using the Cystine Uptake Assay Kit (Dojindo, #UP05) following the provided protocol. Cells were seeded in 96-well plates. Prior to the assay, cells were washed and incubated in cysteine-free serum-free medium for 5 min. The assay was initiated by adding the provided CA Uptake Solution to each well. After incubation at 37 °C for 30 min, the CA Uptake Solution was aspirated, and cells were washed thoroughly with ice-cold PBS to terminate uptake. The intracellular cystine content was measured using the Working Solution provided in the kit, and fluorescence was detected with a fluorescence microplate reader at Ex = 490 nm and Em = 535 nm. The fluorescence intensity of the Cystine Analog taken up (CA fluorescence intensity) was calculated by subtracting the Blank measurement from the sample measurement. Subsequently, the samples were stained with the nucleic acid dye Hoechst 33342, and fluorescence was measured using a microplate reader at Ex = 350 nm and Em = 461 nm. The fluorescence intensity corresponding to the number of cells (Hoechst 33342 fluorescence intensity) was calculated by subtracting the blank measurement from the sample measurement values. Cystine uptake = CA fluorescence intensity/Hoechst 33342 fluorescence intensity. All experiments were performed in triplicate.

### Statistical information

All quantitative data are presented as the mean ± standard deviation (SD) from at least three independent biological replicates, unless otherwise specified in the figure legends. Statistical analyses were performed using GraphPad Prism software (version 8.0.0). The specific statistical tests applied to each experiment are detailed in the corresponding figure legends. Briefly, comparisons between two groups were analyzed using the unpaired, two-tailed Student’s *t*-test. For comparisons among multiple groups, one-way analysis of variance (ANOVA) with Tukey’s post-hoc test was employed. Two-way ANOVA with Sidak’s multiple comparisons test was used to analyze data involving two independent variables. *P* values are indicated in the panels.

## Supplementary information

Supplementary information

Supplementary information of WB

## Data Availability

All data generated and analyzed in this study have been included within this article and its additional files. Further details are available from the corresponding author upon request. The RNA sequencing data and RIP sequencing data generated in this study have been deposited in the NCBI Sequence Read Archive (SRA) and are available under the BioProject accession number PRJNA1366110 and PRJNA1366134.
