# Supplementary material for: Targeting RBM41-mediated CKMT1B mRNA stability triggers disulfidptosis and suppresses tongue squamous cell carcinoma
Source: Cell Death Dis. 2026 Jul 31;17(1):914. doi: 10.1038/s41419-026-09166-2 (PMC13720023; doi:10.1038/s41419-026-09166-2)
Supplement: Supplementary file 1 [file 41419_2026_9166_MOESM2_ESM.pdf]

Fig 1D

GAPDH

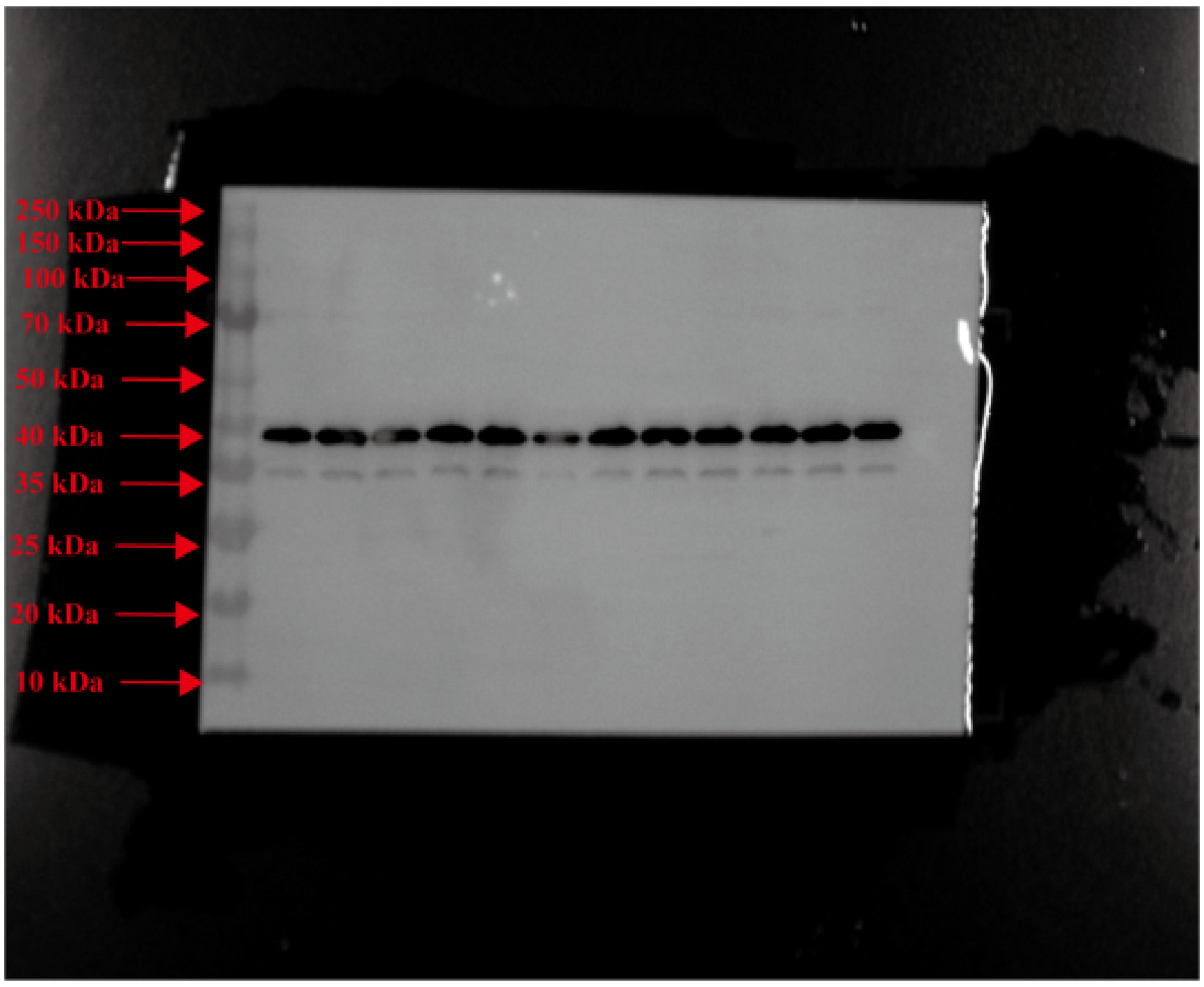

RBM41

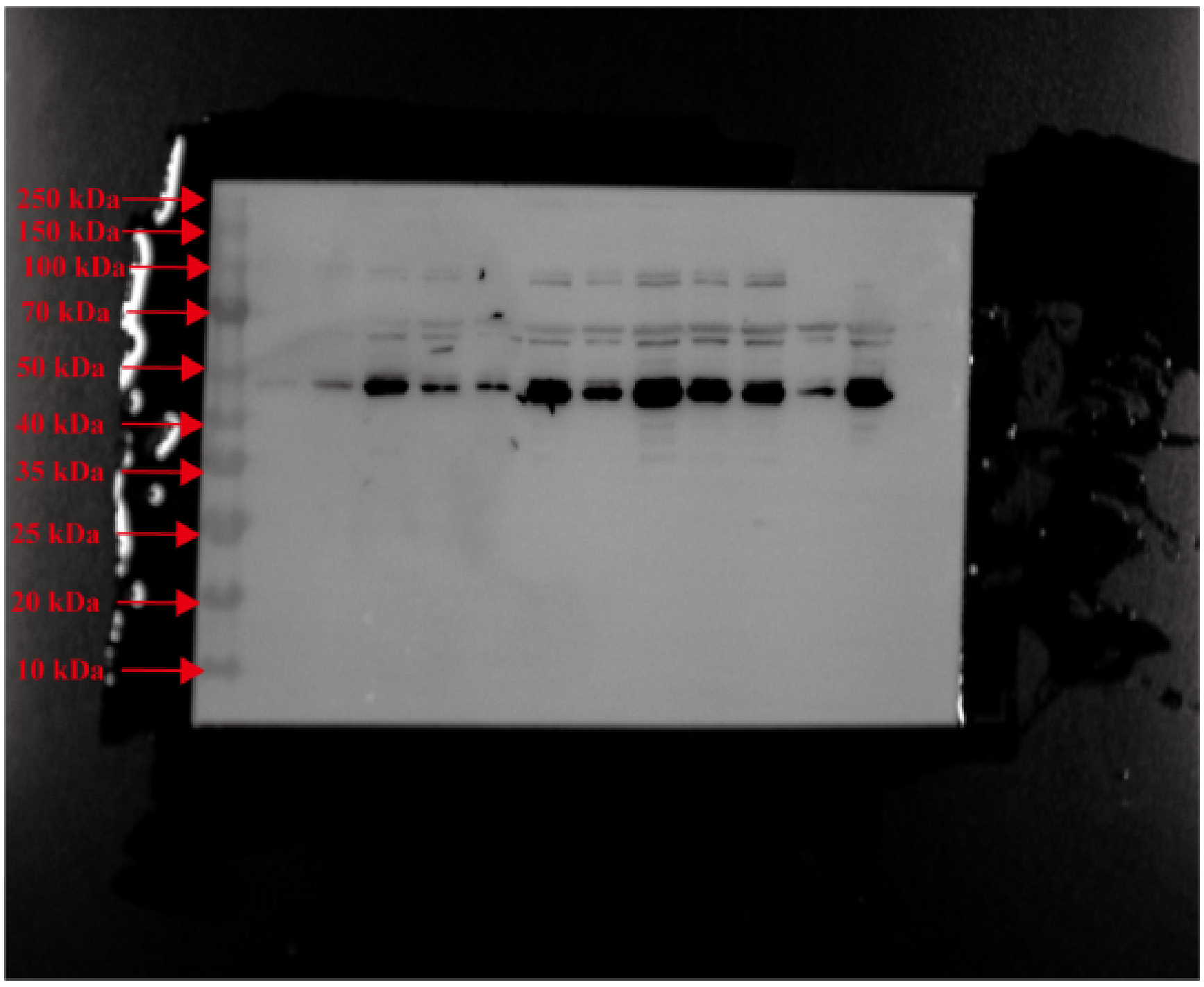

Fig 3B

GAPDH

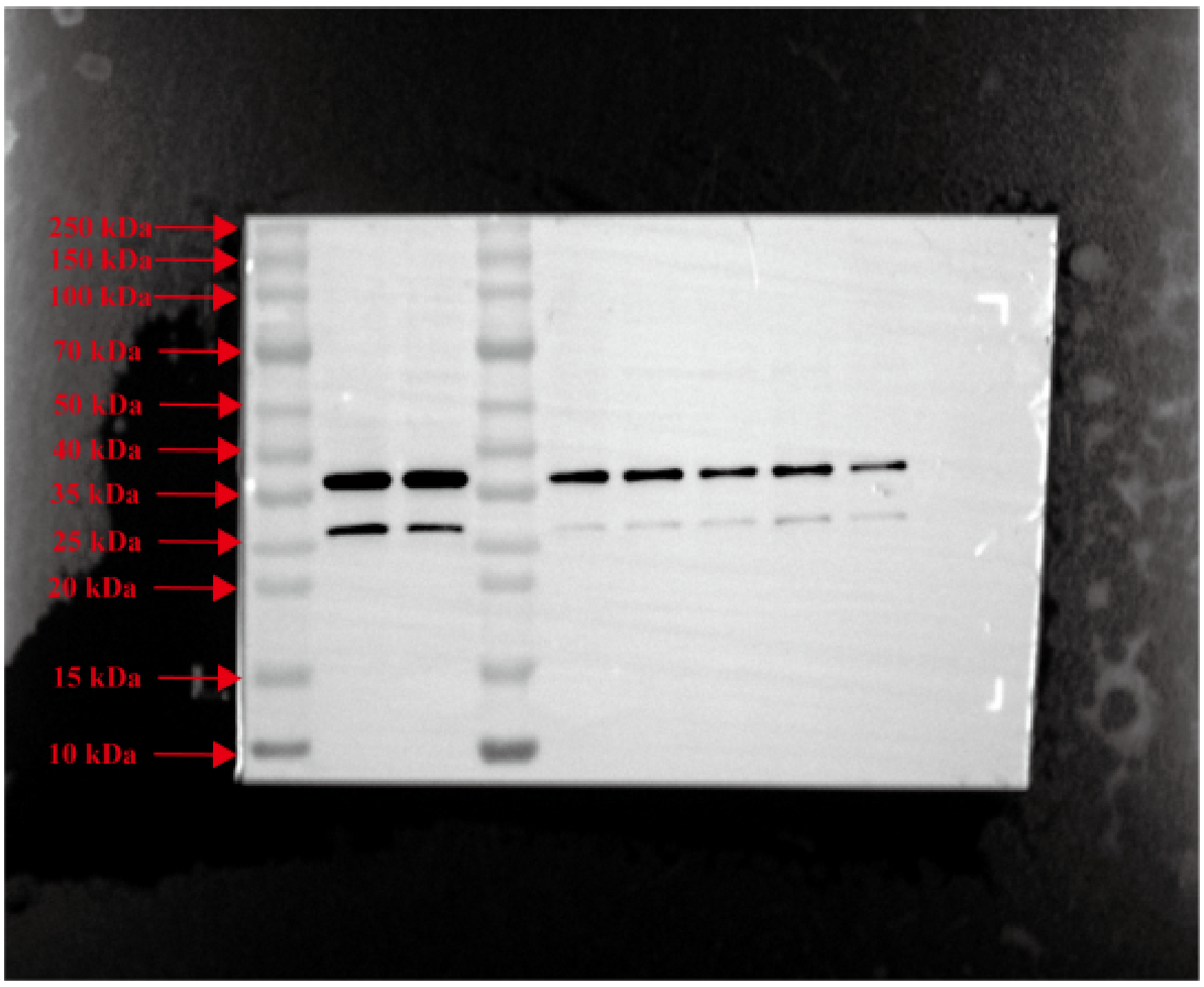

RBM41

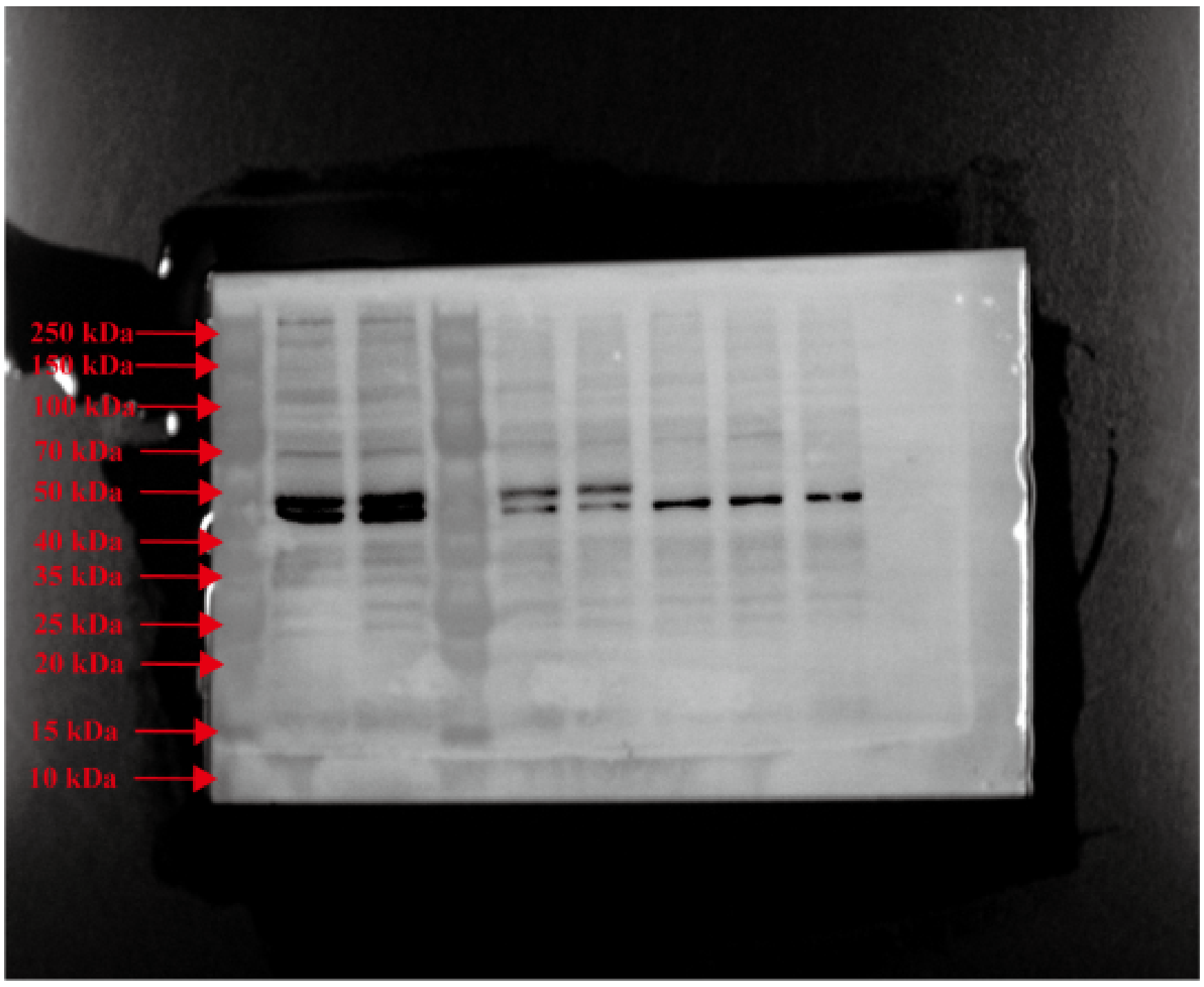

Fig 4M

Drebrin

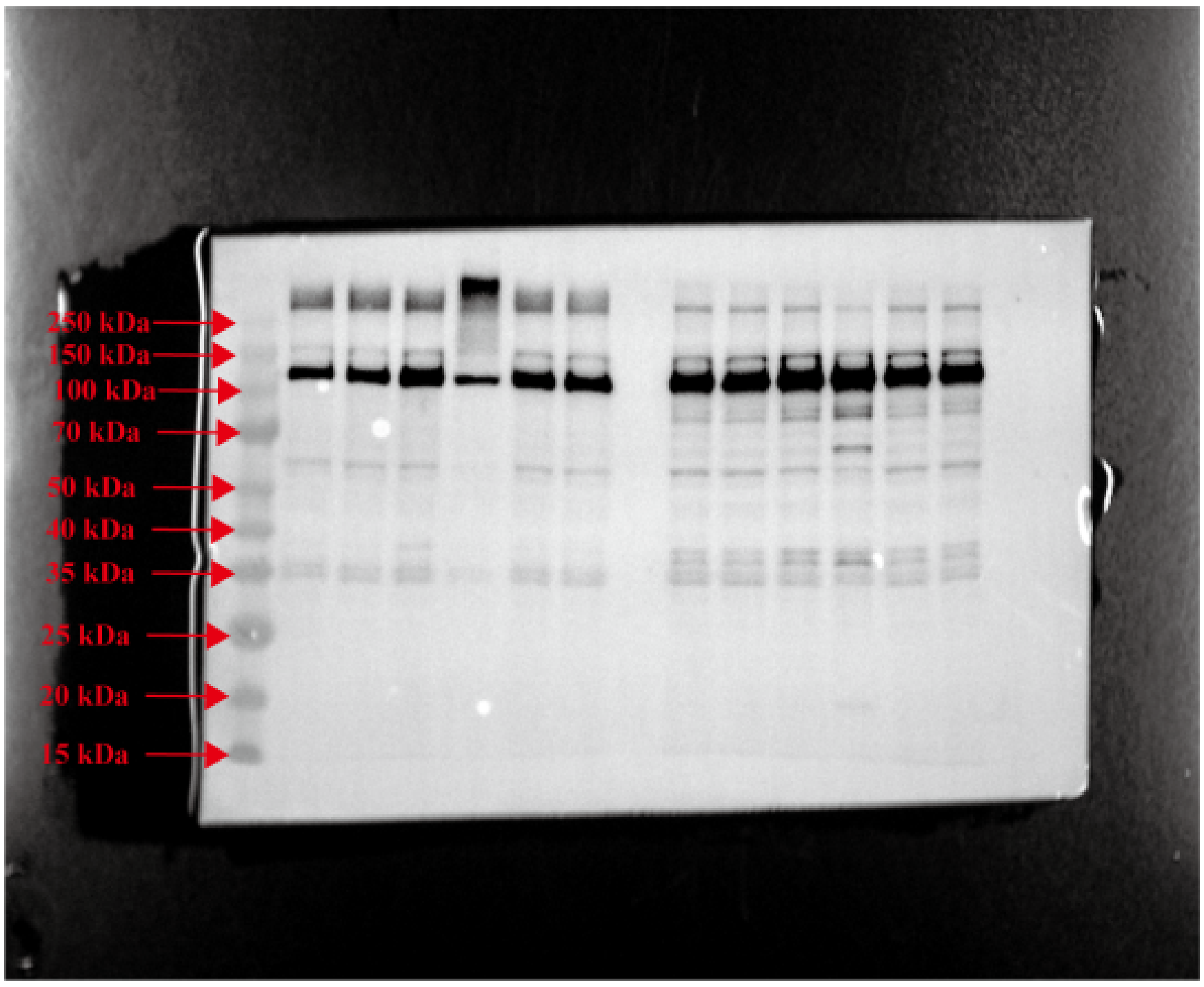
250 kDa
150 kDa
100 kDa
70 kDa
50 kDa
40 kDa
35 kDa
25 kDa
20 kDa
15 kDa

FLNA

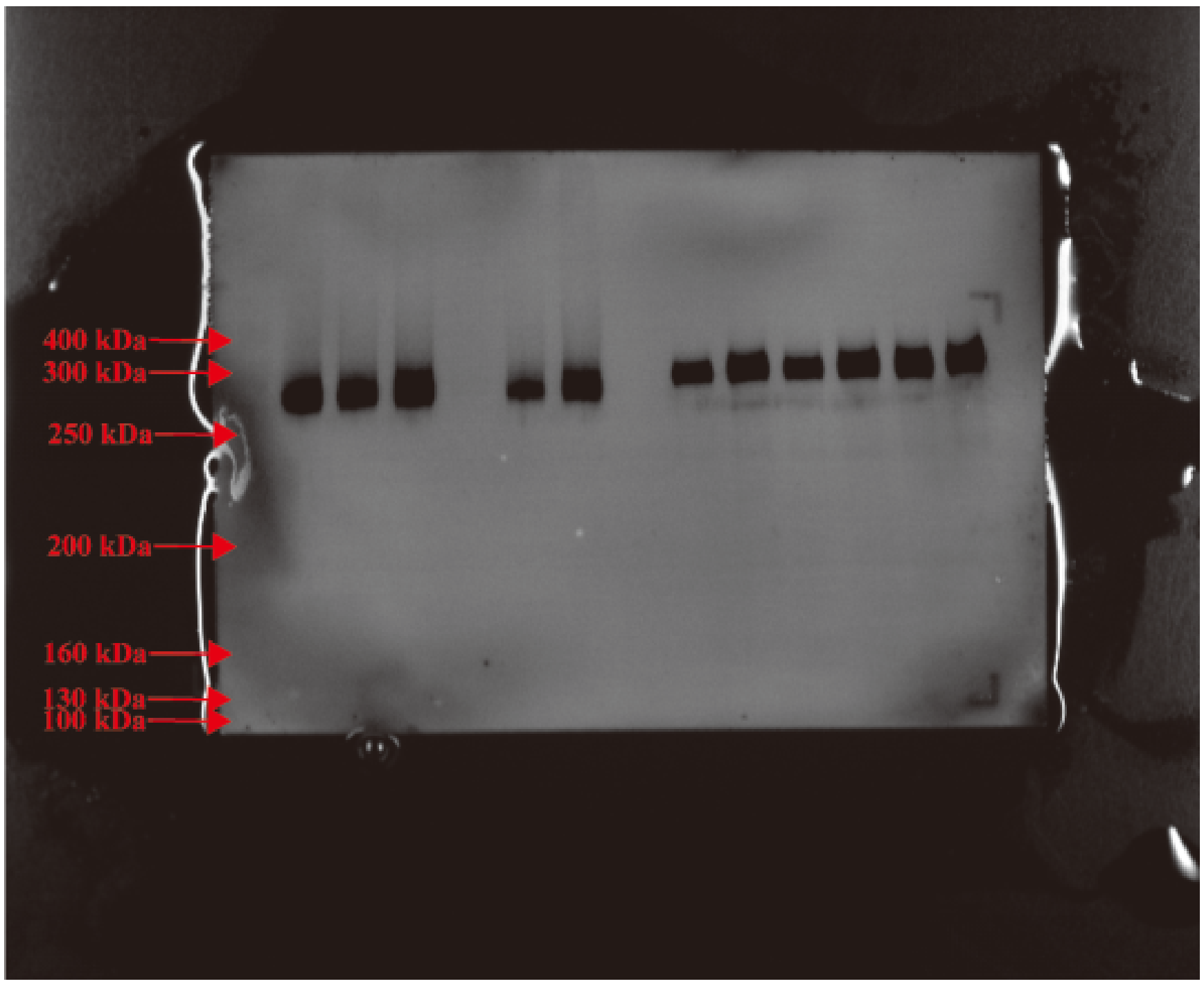
400 kDa
300 kDa
250 kDa
200 kDa
160 kDa
130 kDa
100 kDa

FLNB

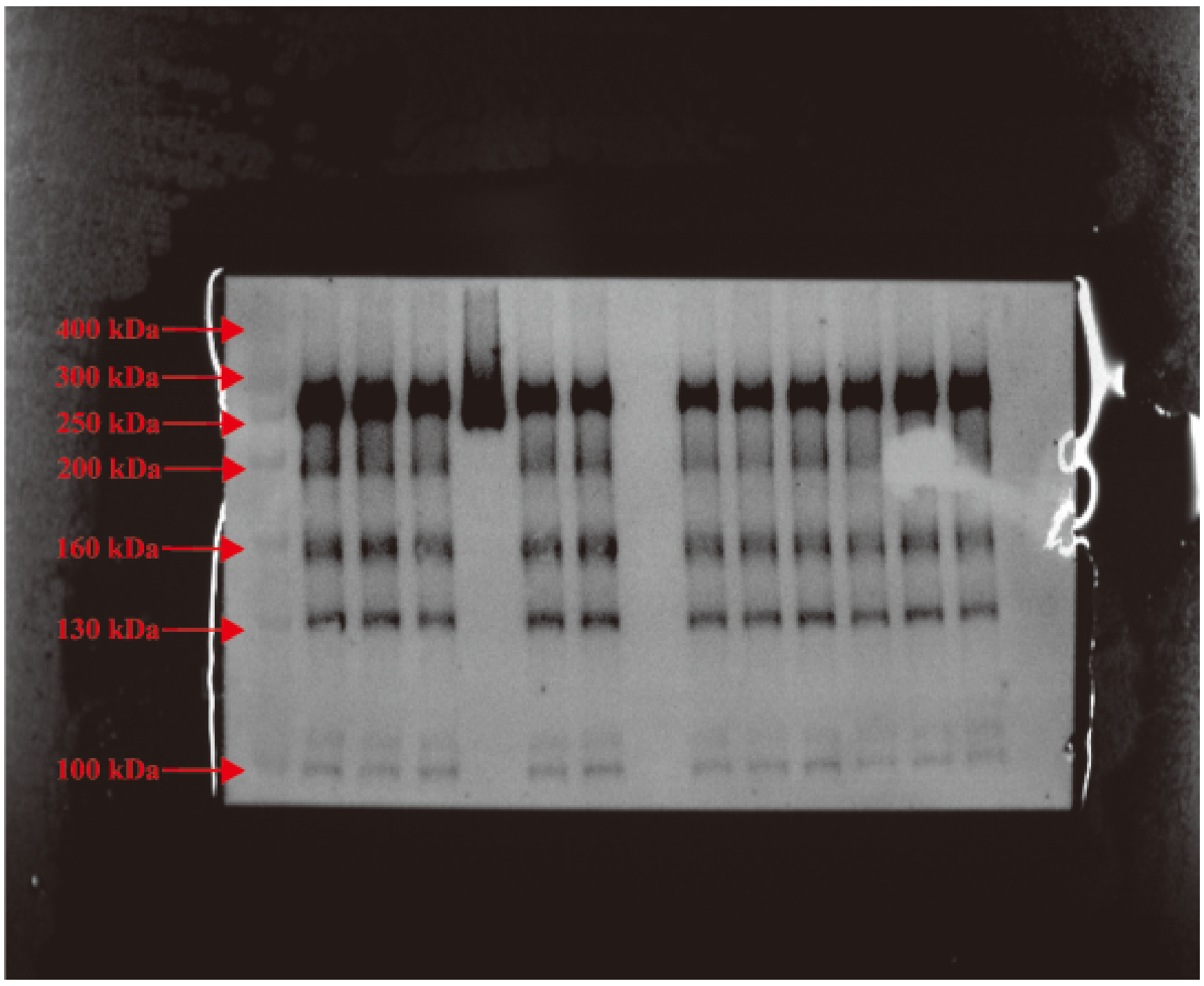

MYH9

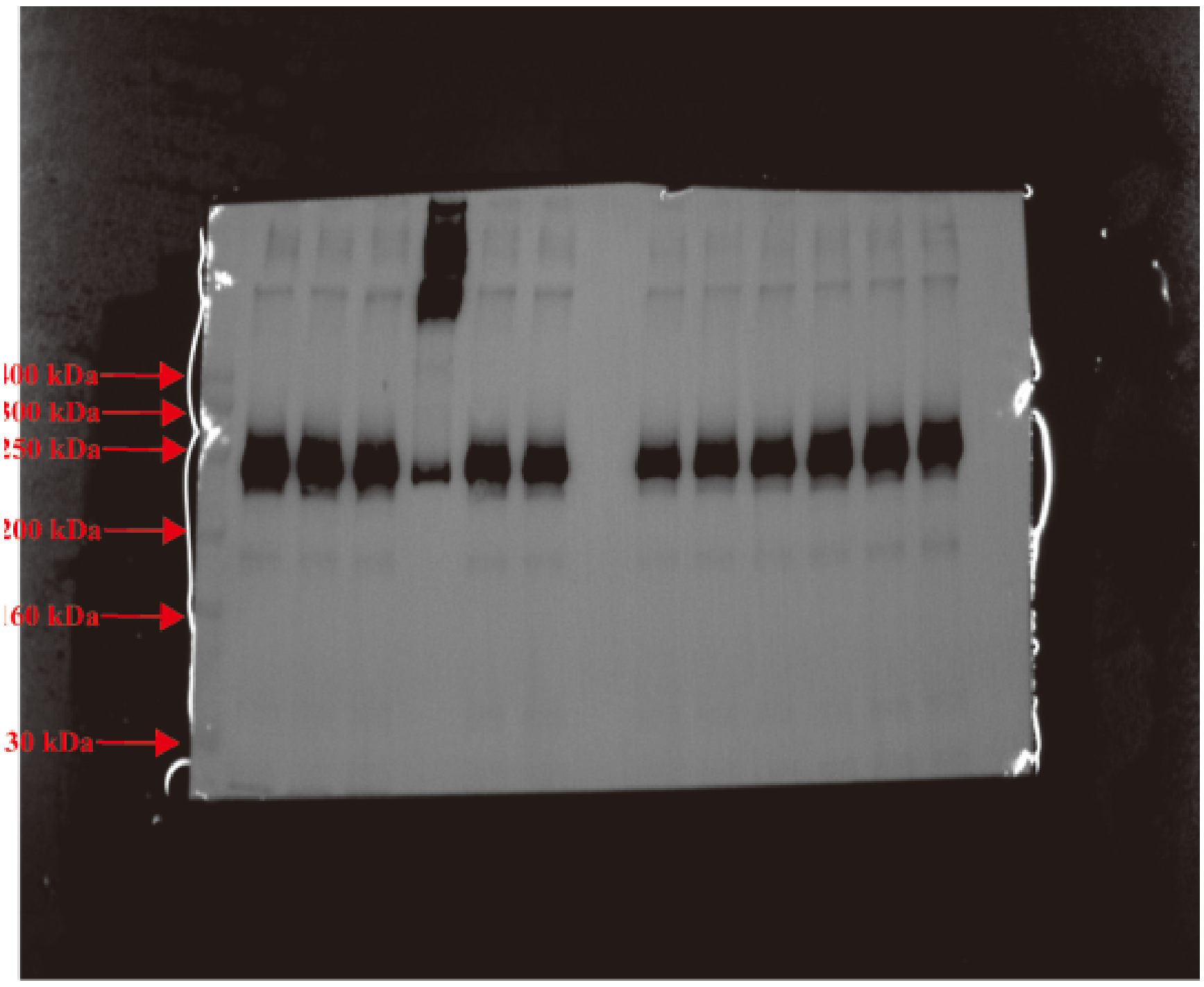

MYH10

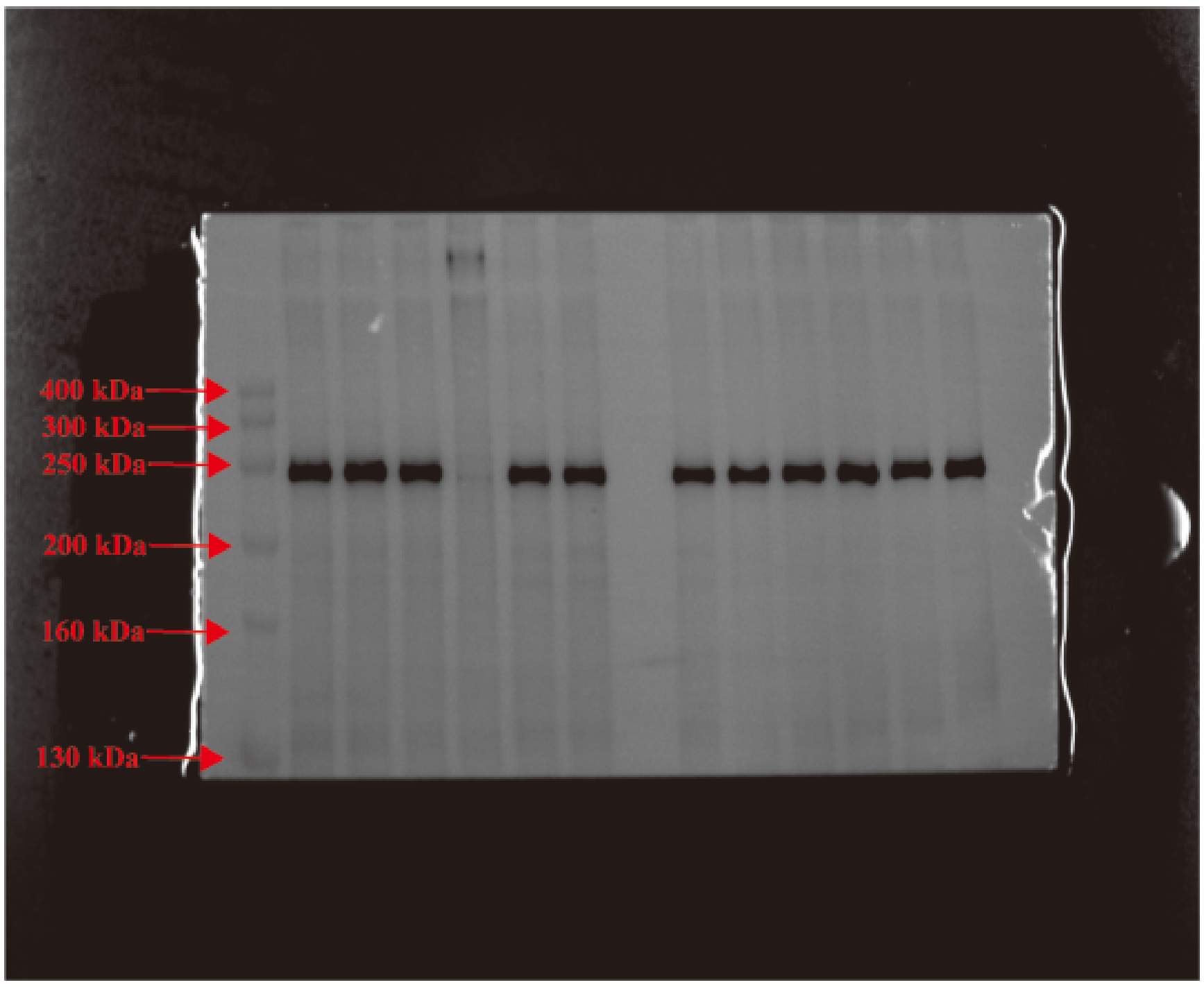

Fig 5E

GAPDH

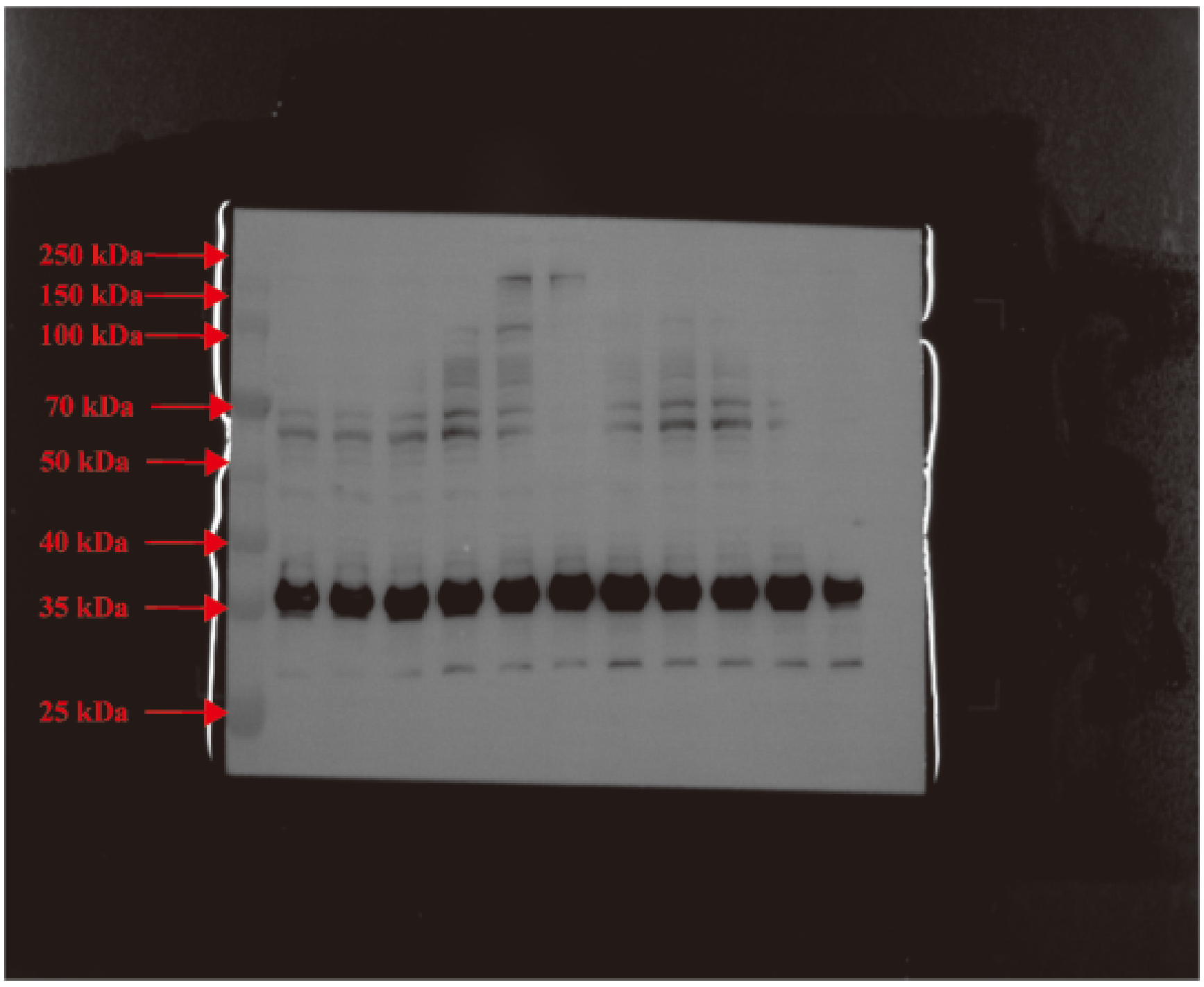

RBM41

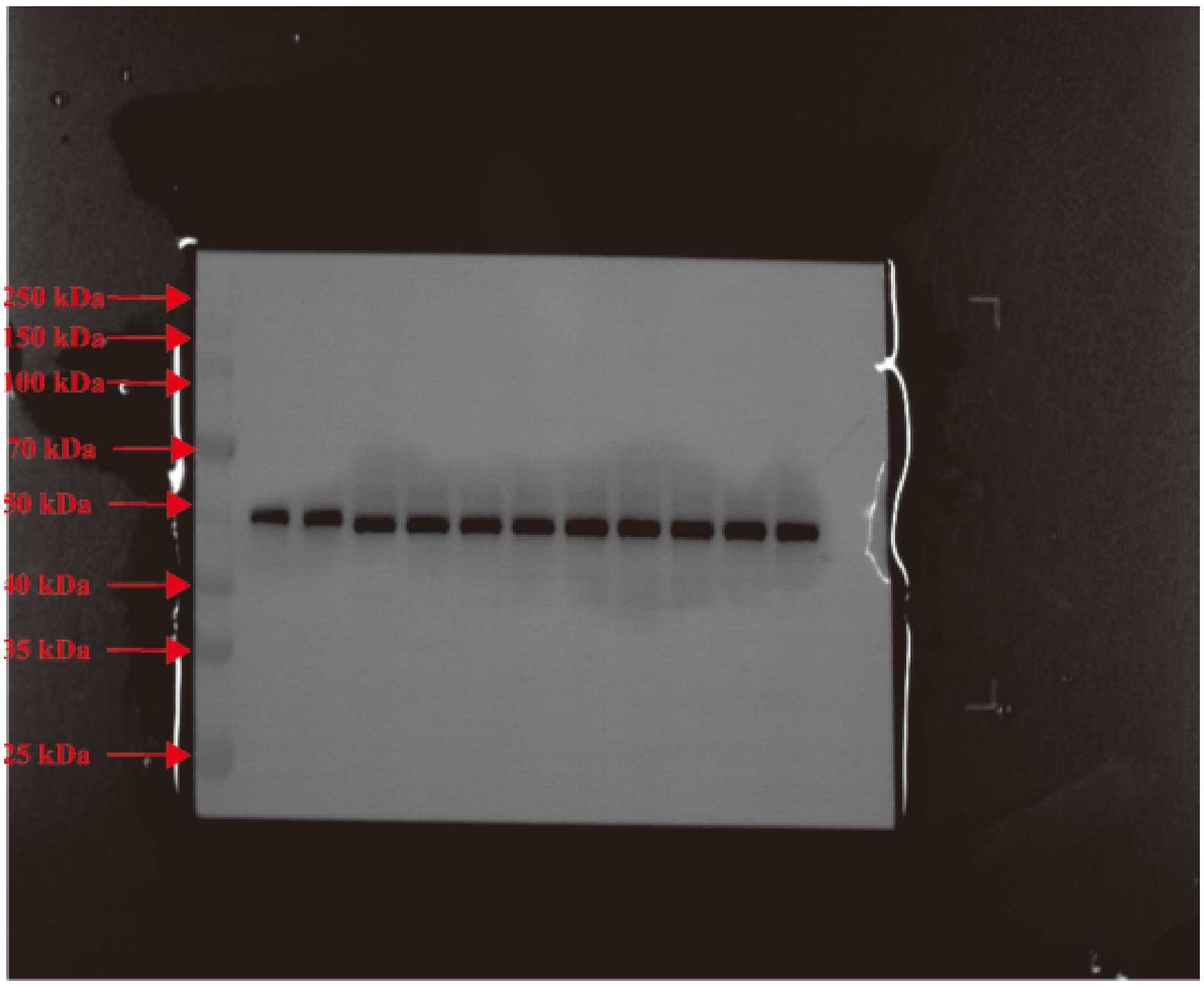

Fig 5N

Drebrin

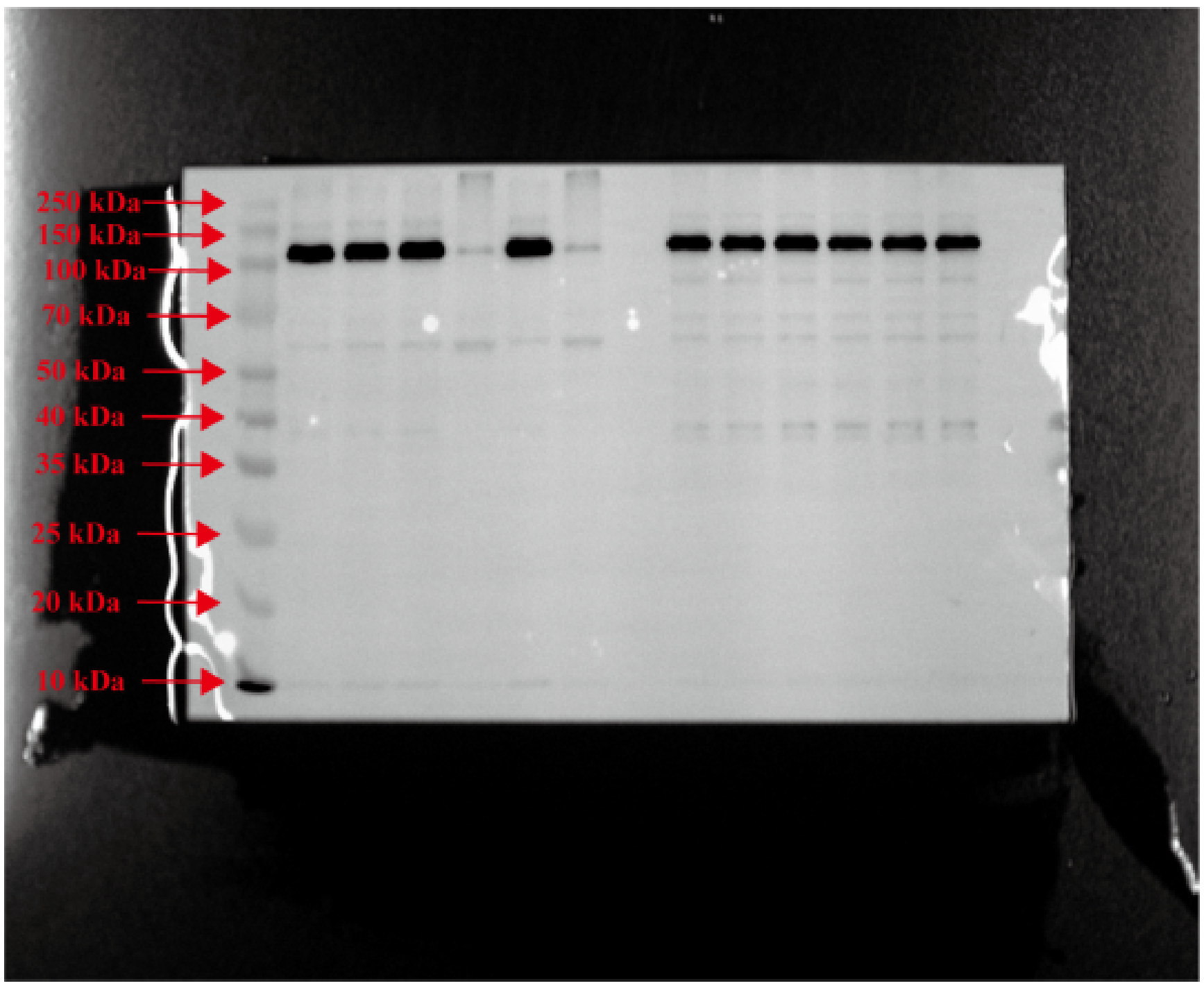

FLNA

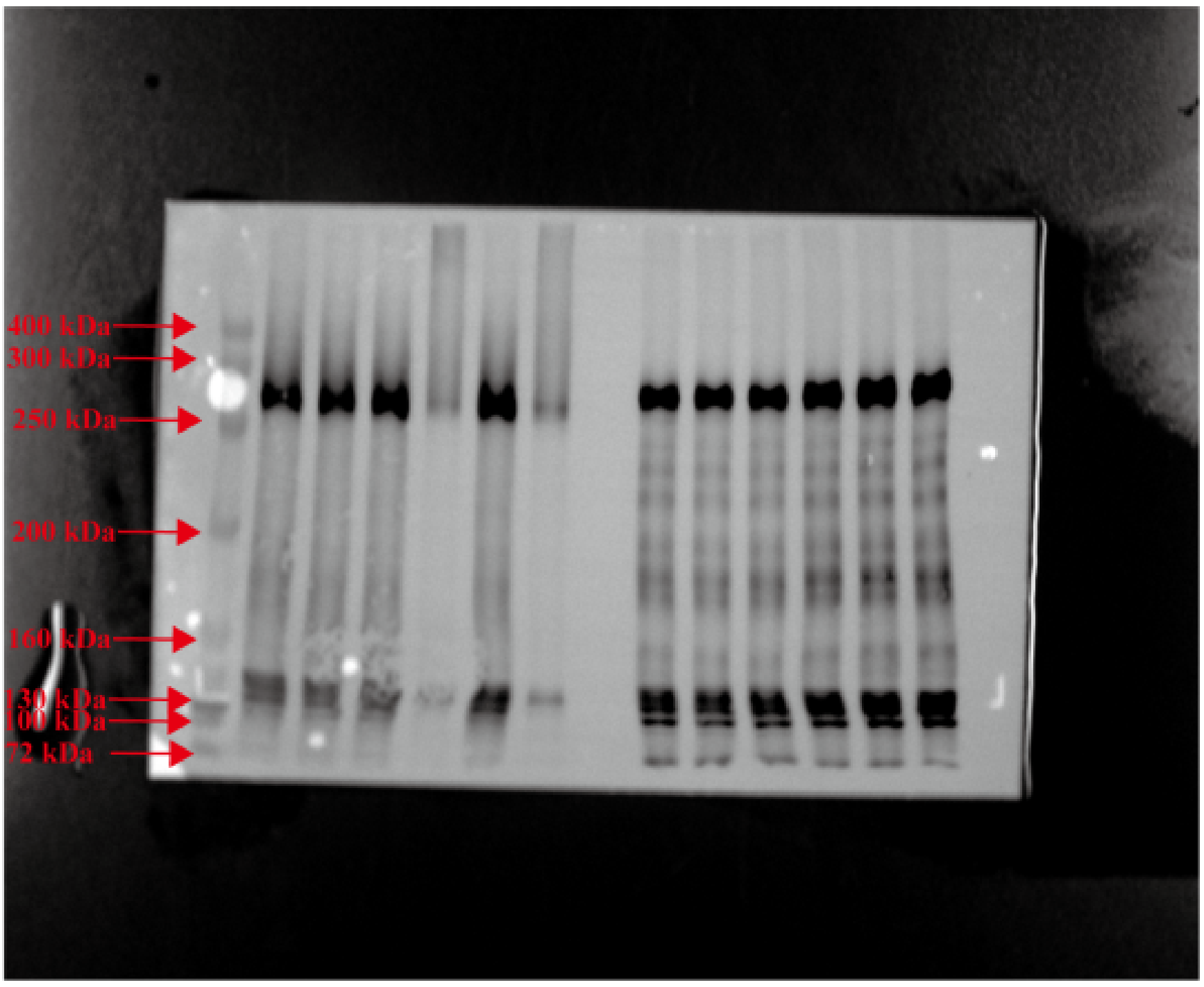
400 kDa
300 kDa
250 kDa
200 kDa
160 kDa
130 kDa
100 kDa
72 kDa

FLNB

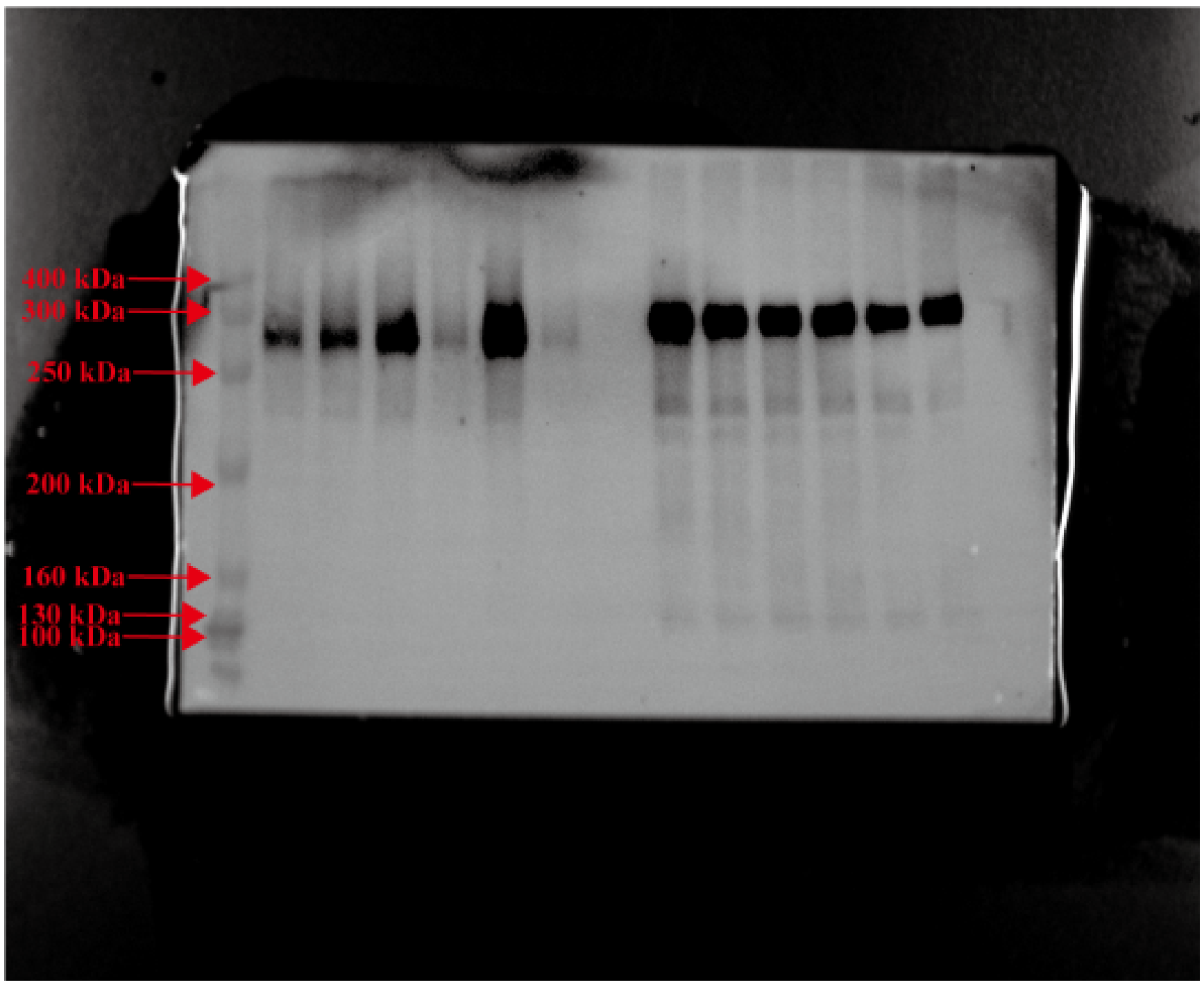
400 kDa
300 kDa
250 kDa
200 kDa
160 kDa
130 kDa
100 kDa

MYH9

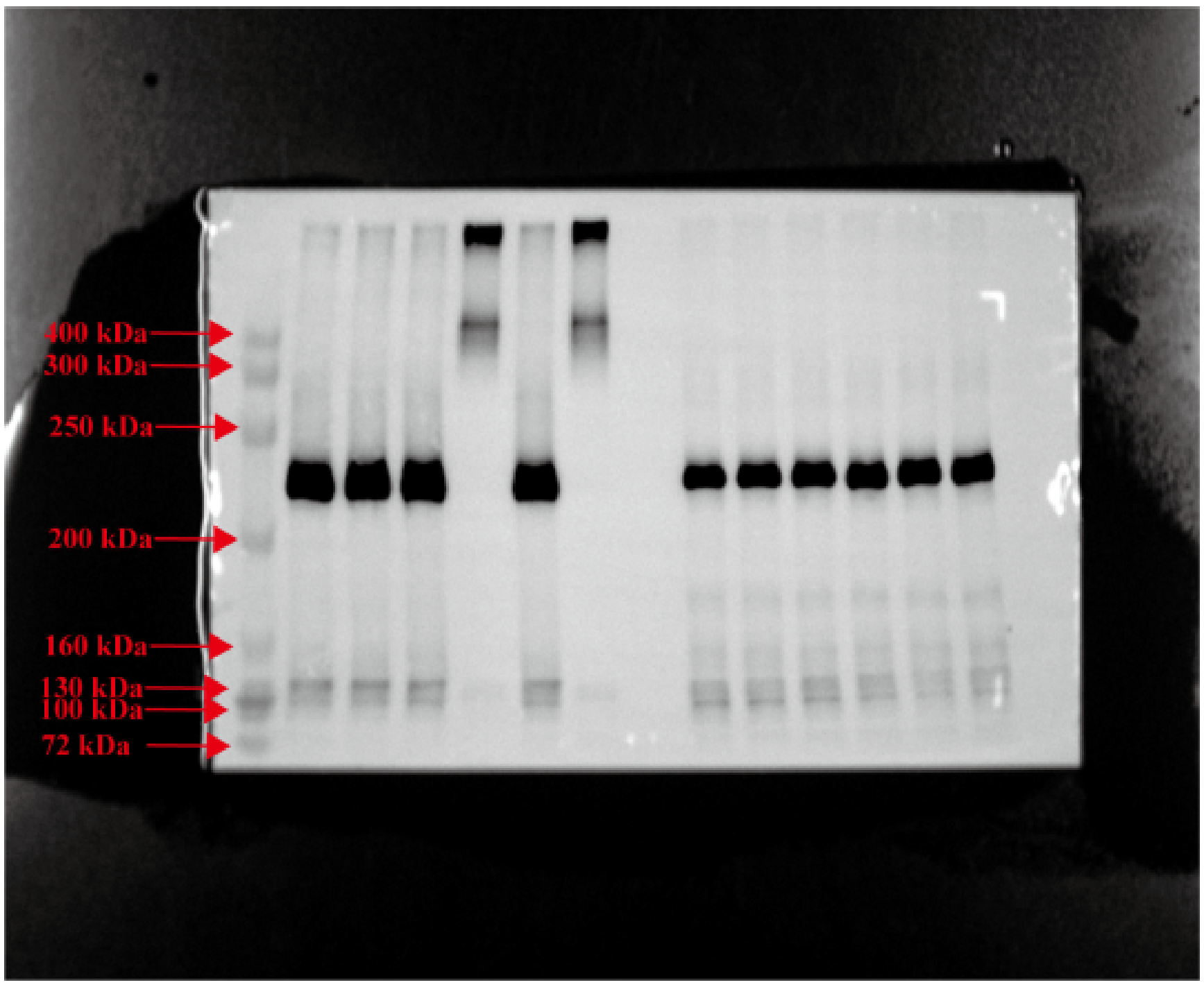

MYH10

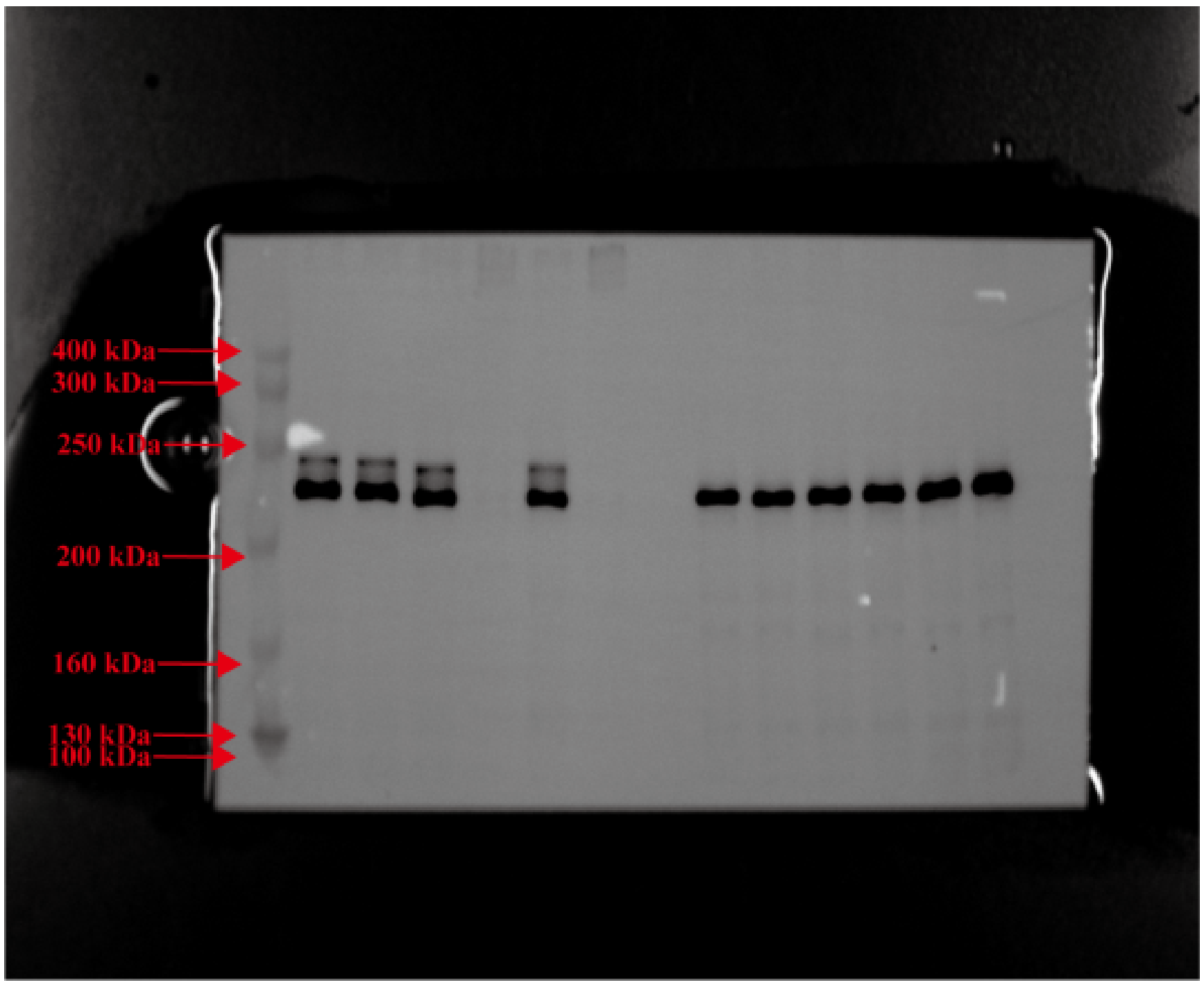

Fig 6I

CKMT1

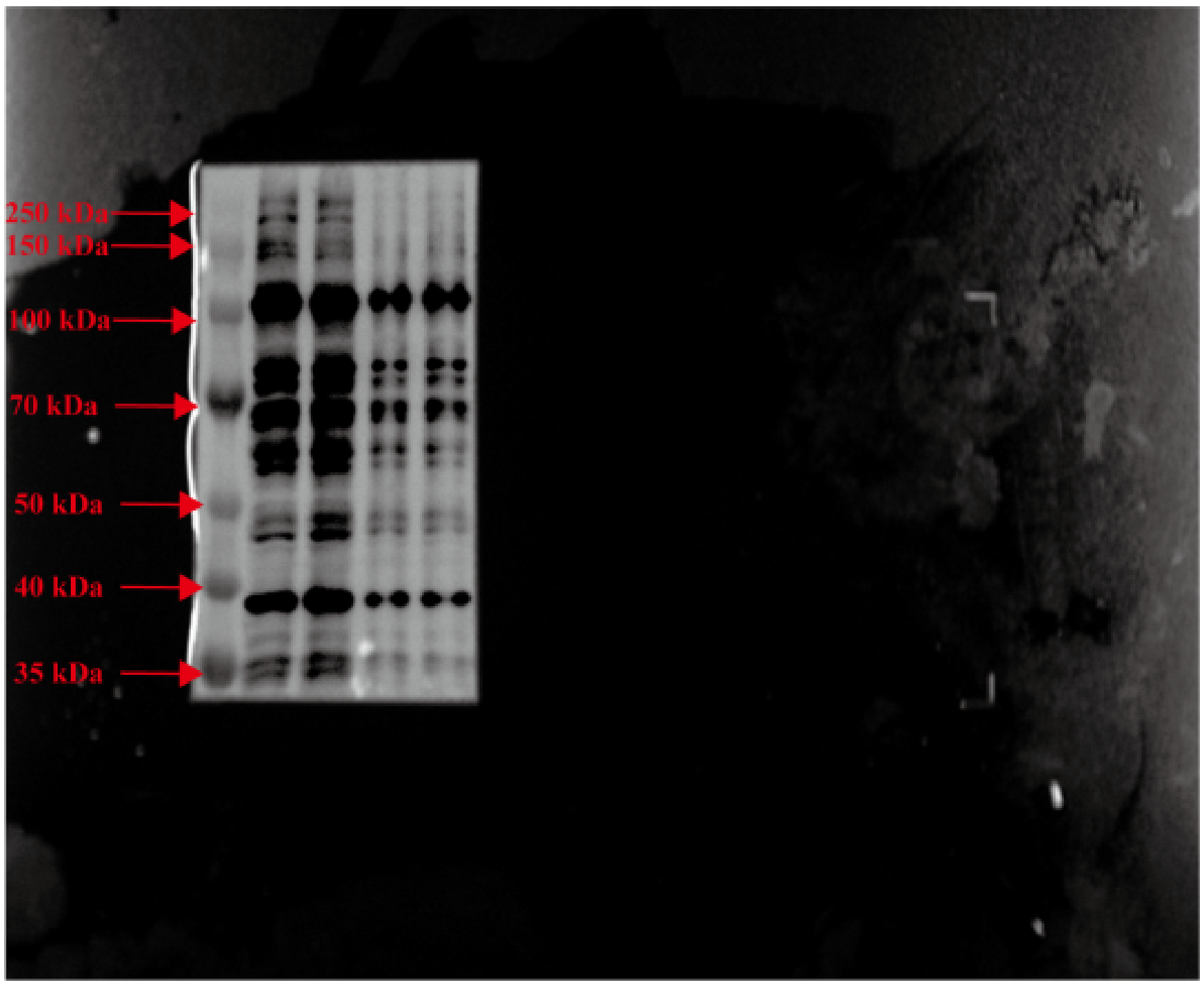

Tubulin

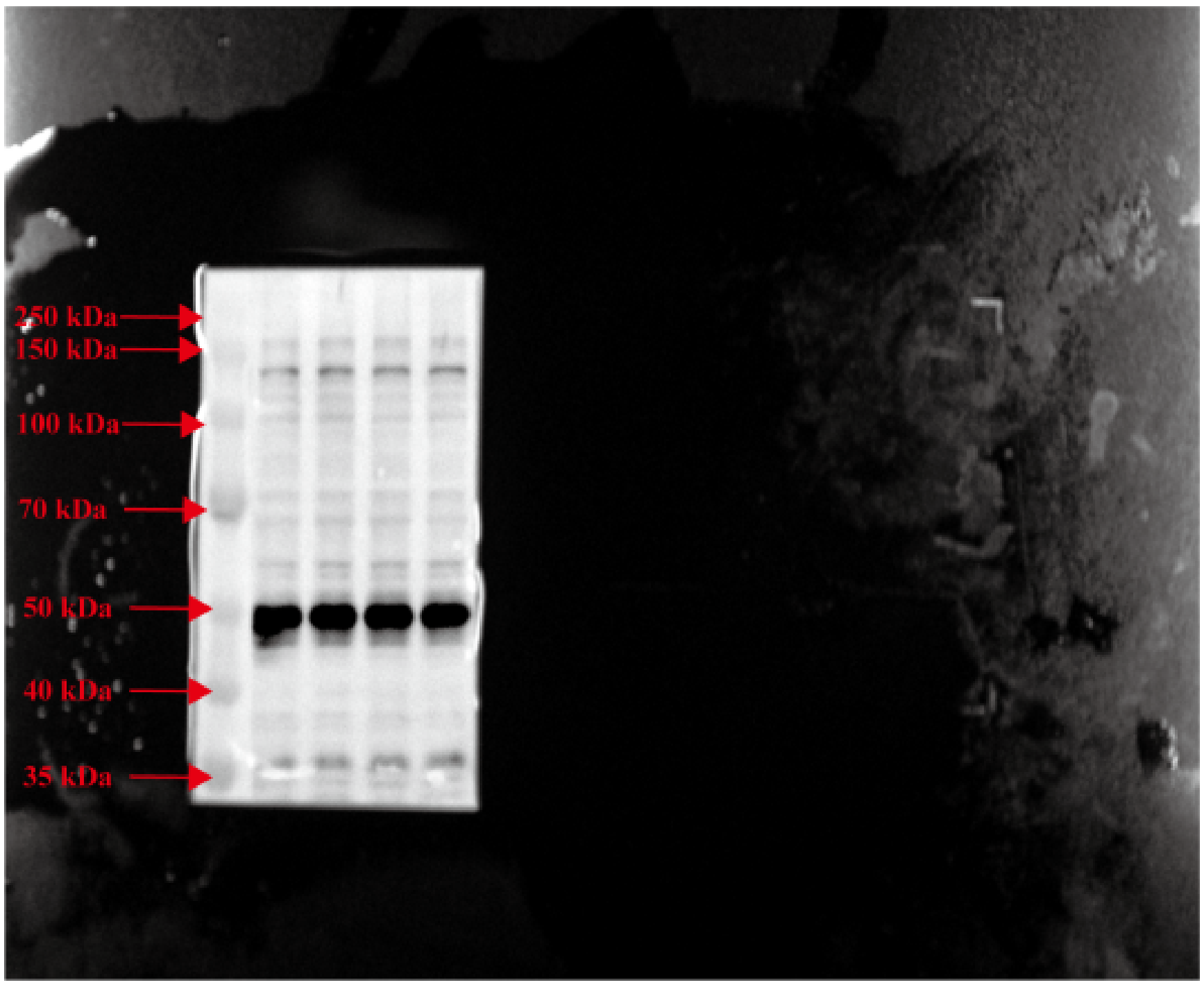

Fig 7H

CKMT1

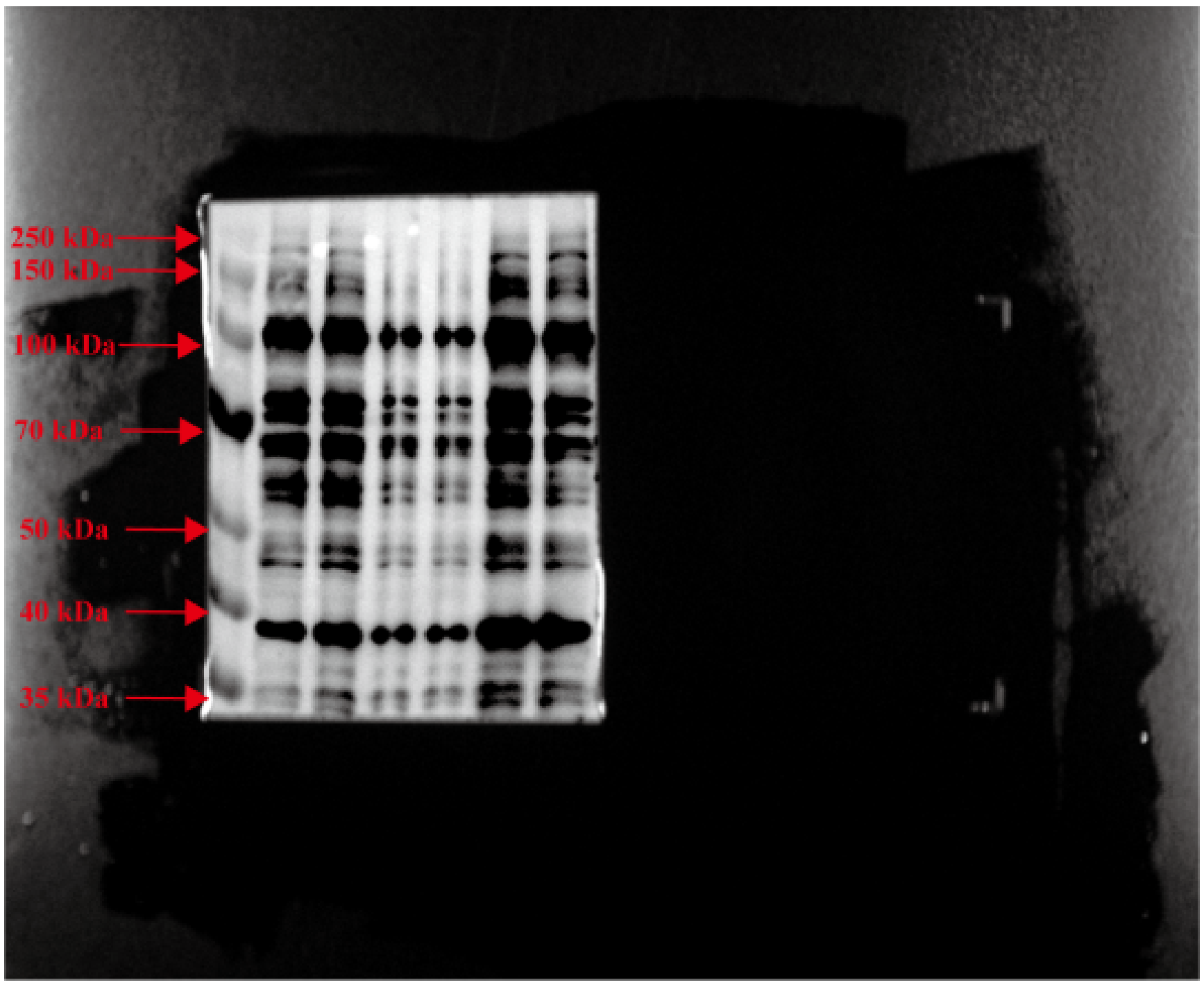

RBM41

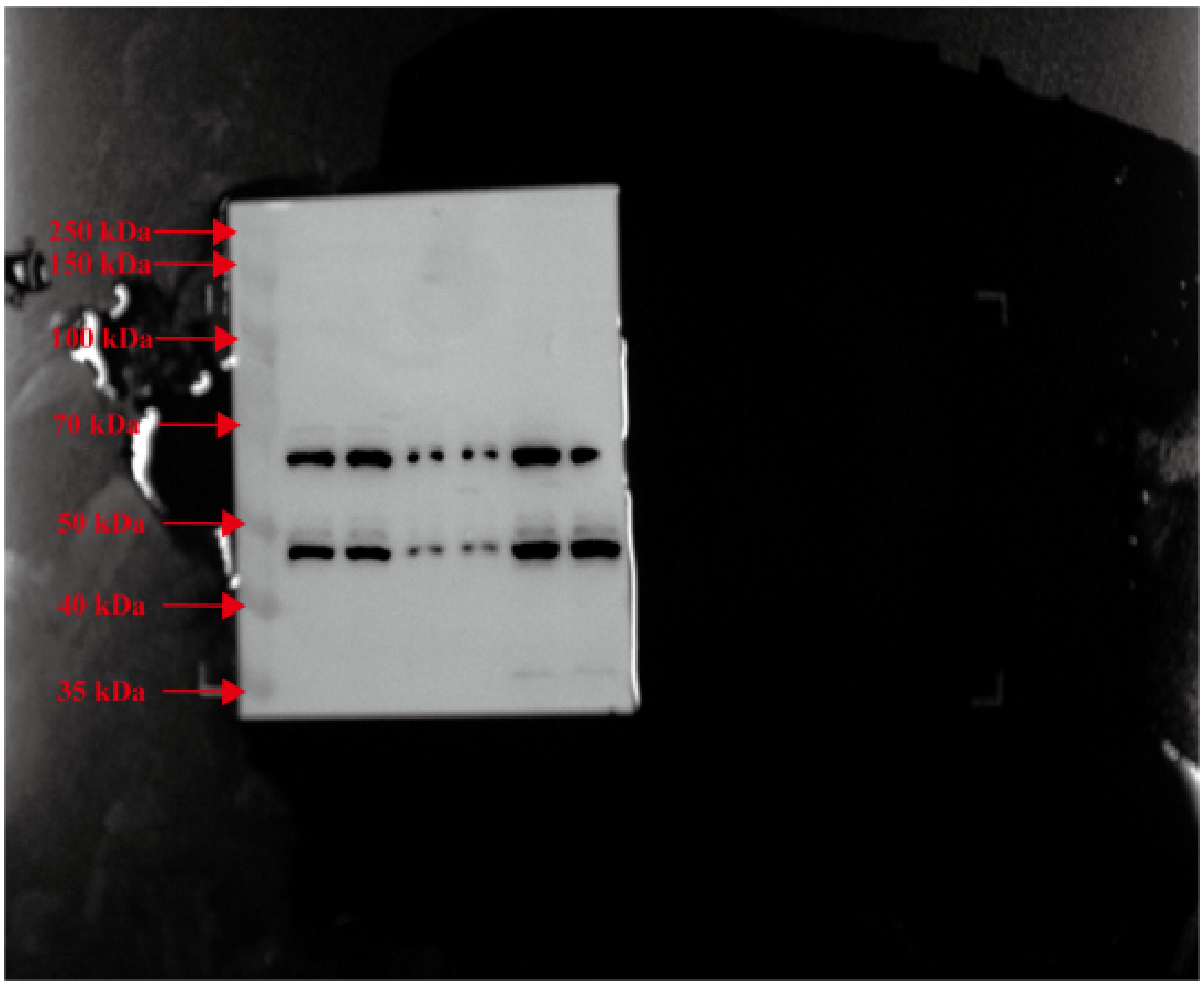

Tubulin

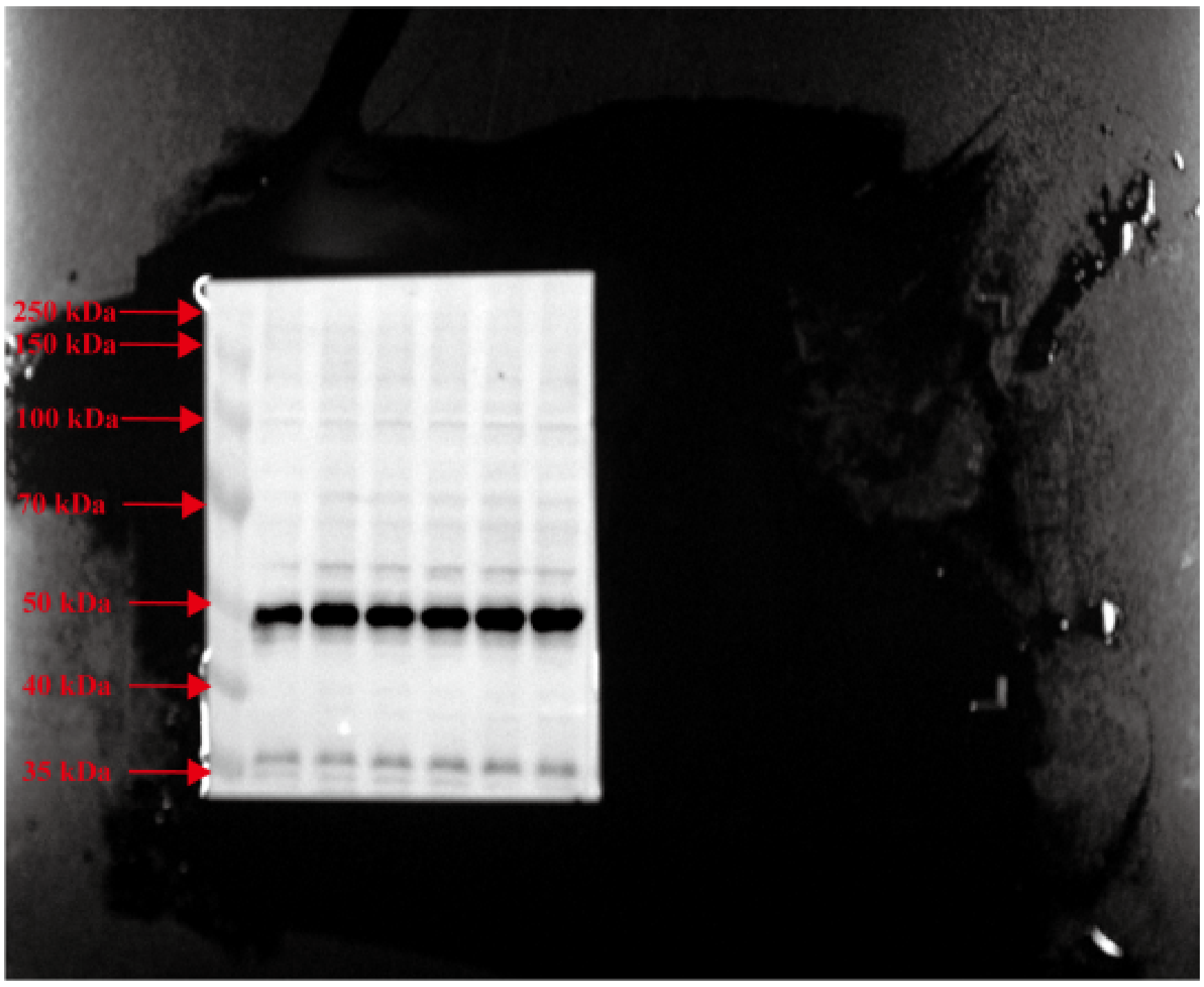

Fig 7I
Drebrin

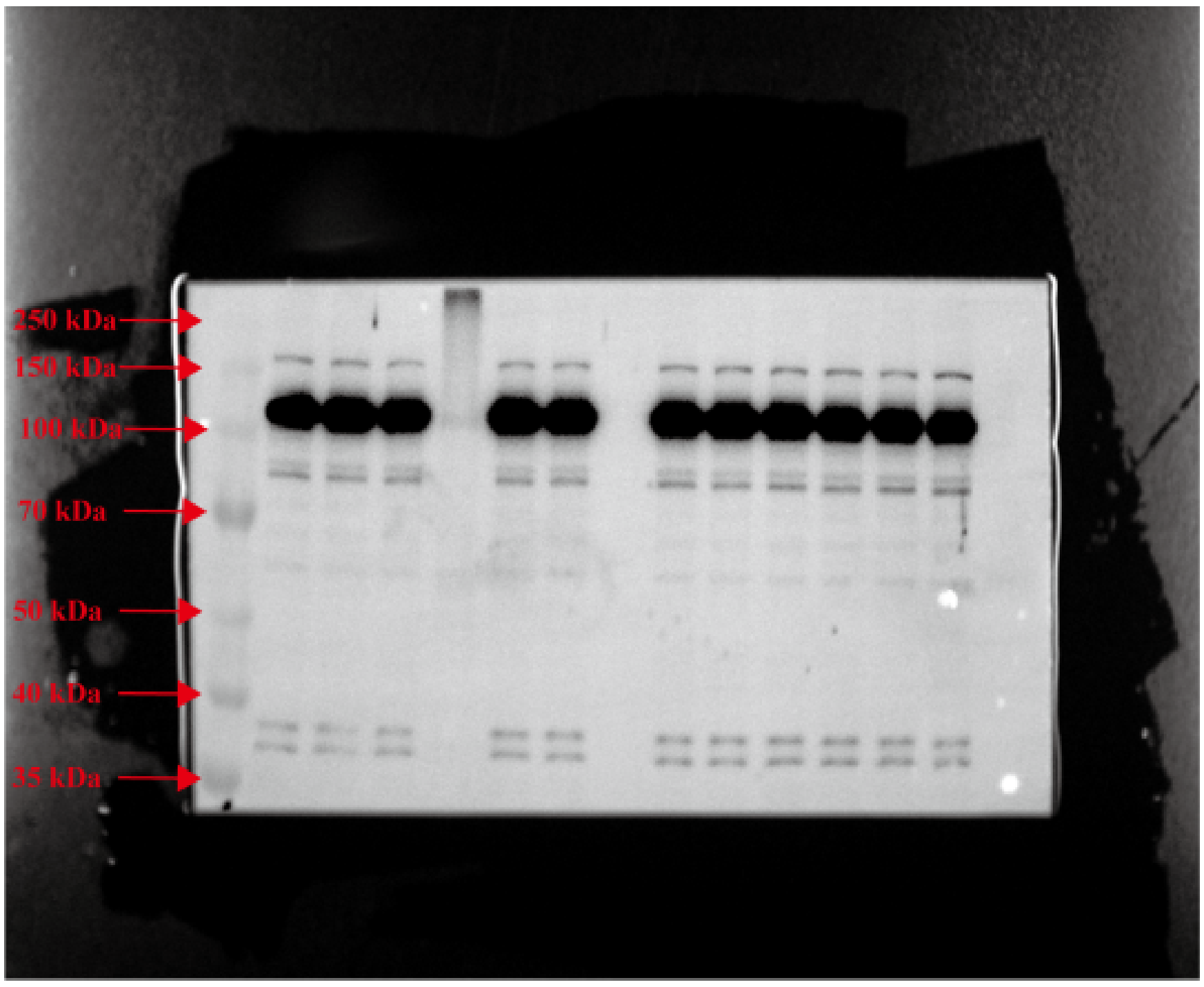

FLNA

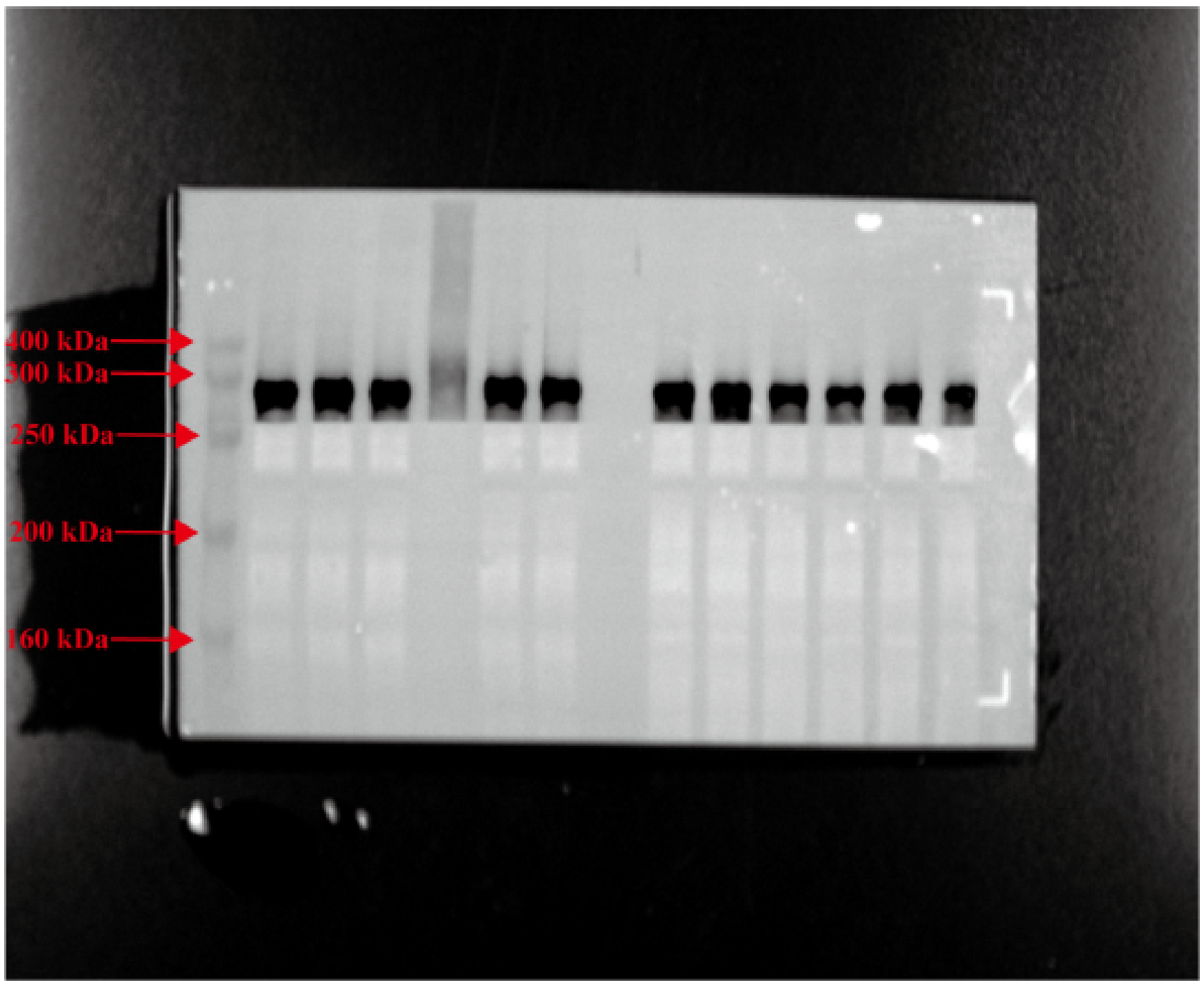
400 kDa
300 kDa
250 kDa
200 kDa
160 kDa
FLNB

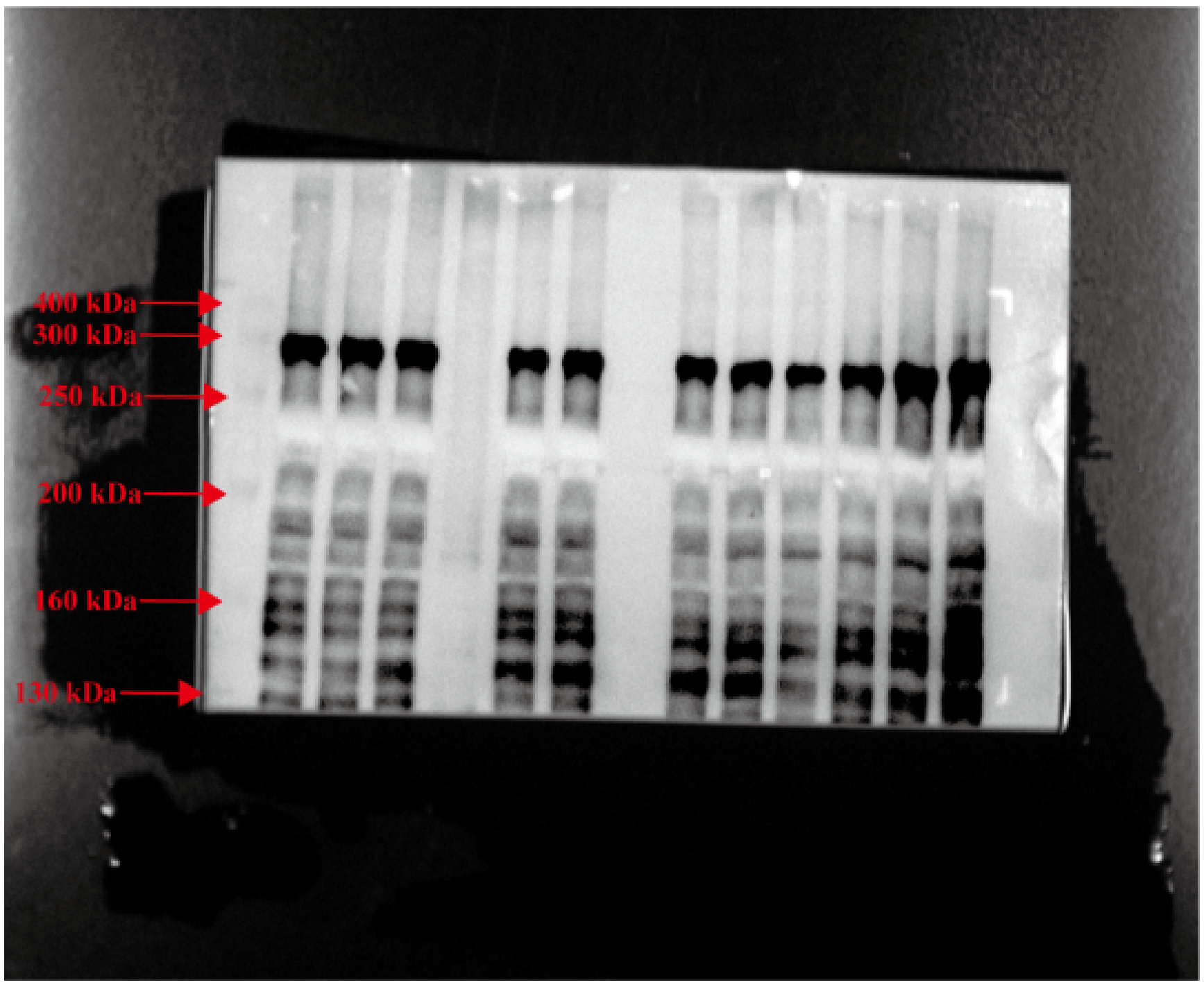
400 kDa
300 kDa
250 kDa
200 kDa
160 kDa
130 kDa
MYH9

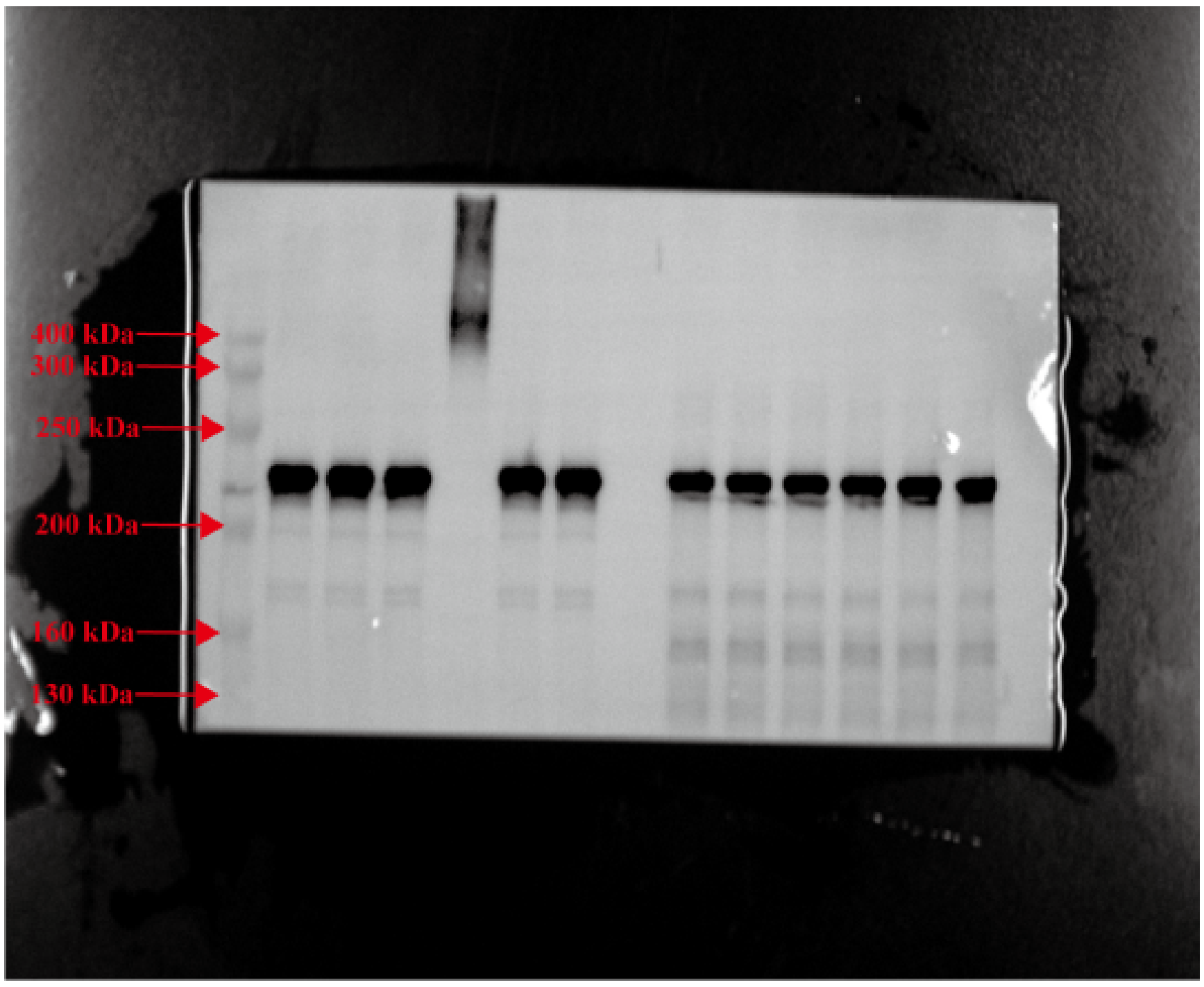

MYH10

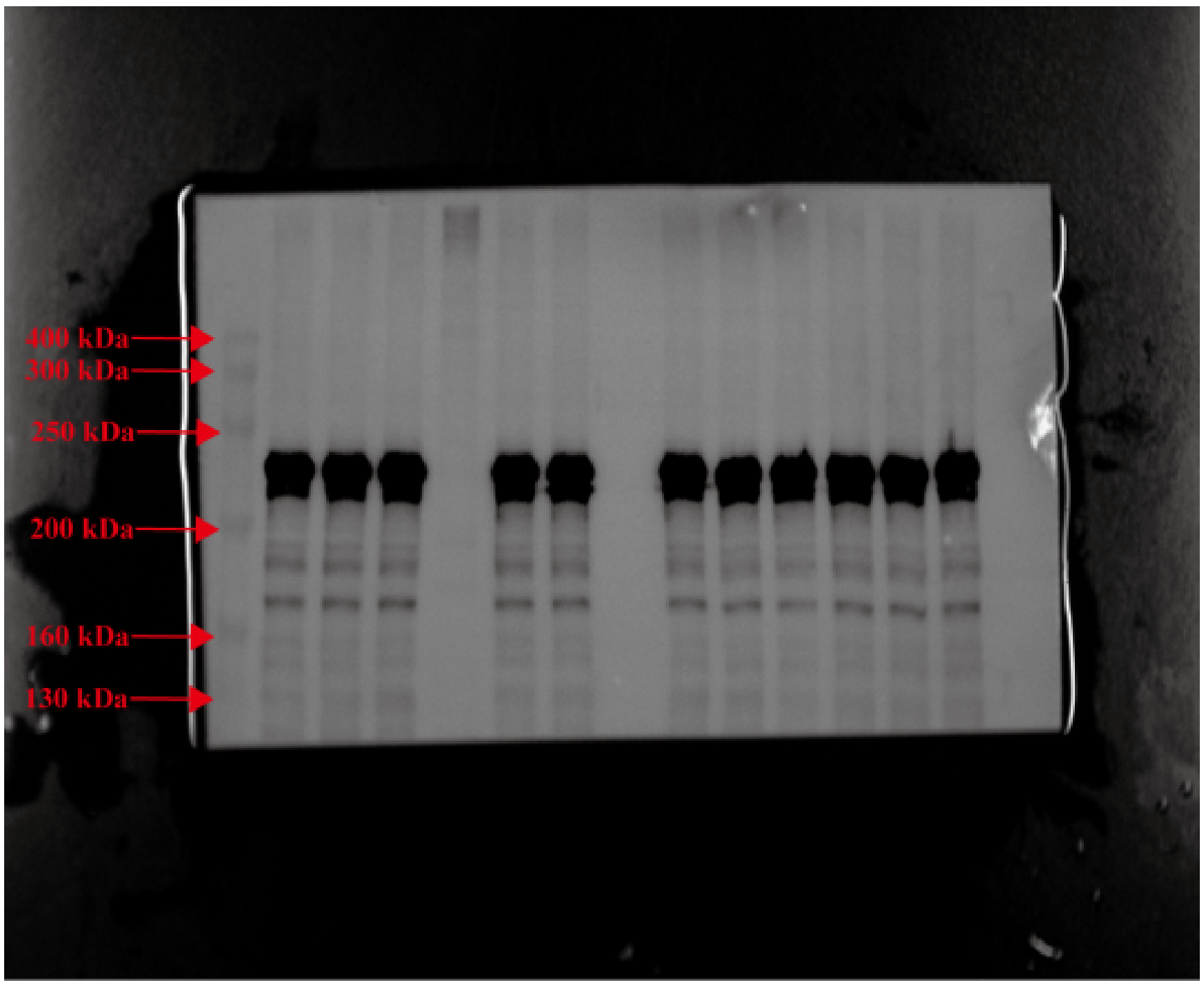

Fig 8J

CKMT1

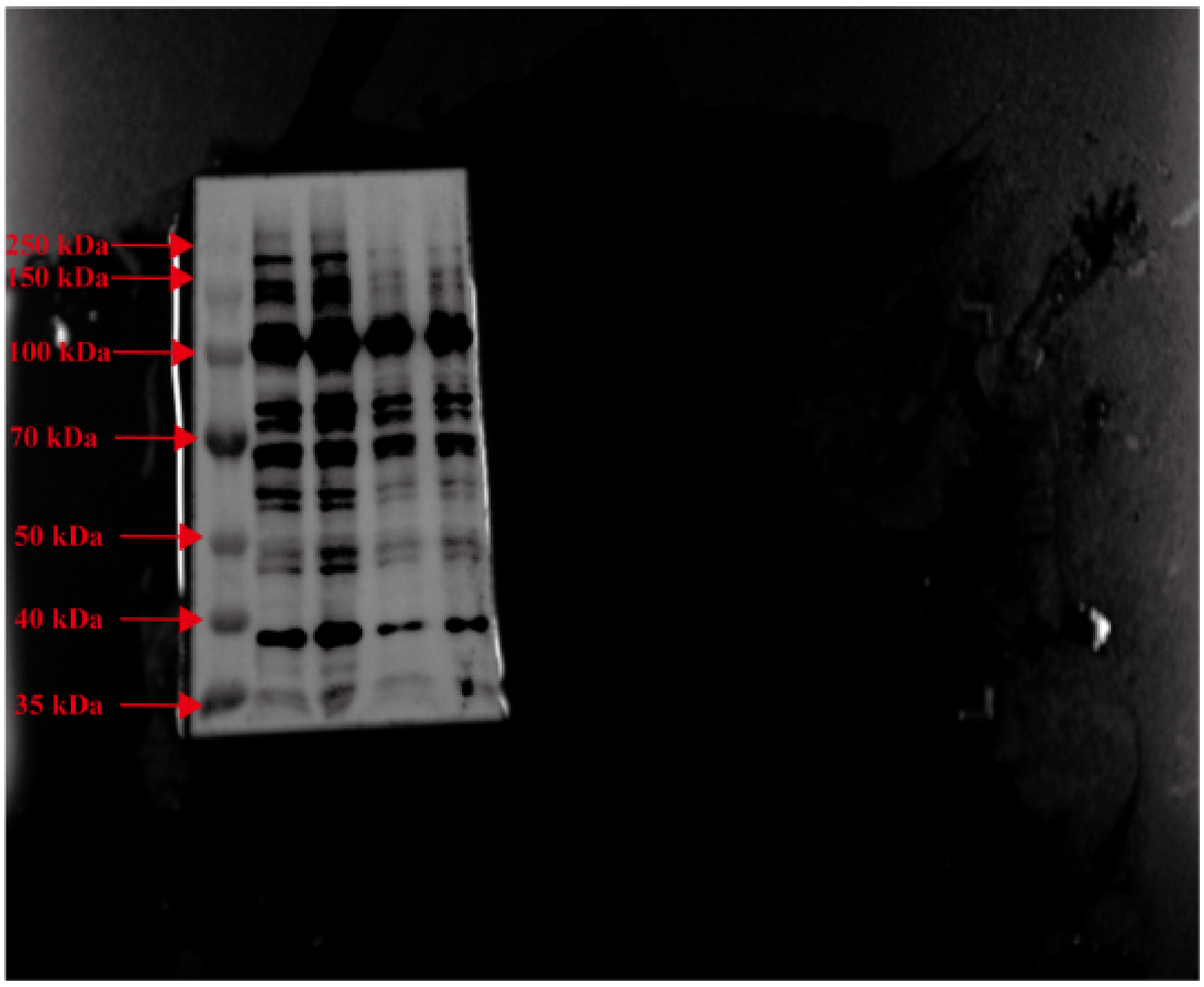

RBM41

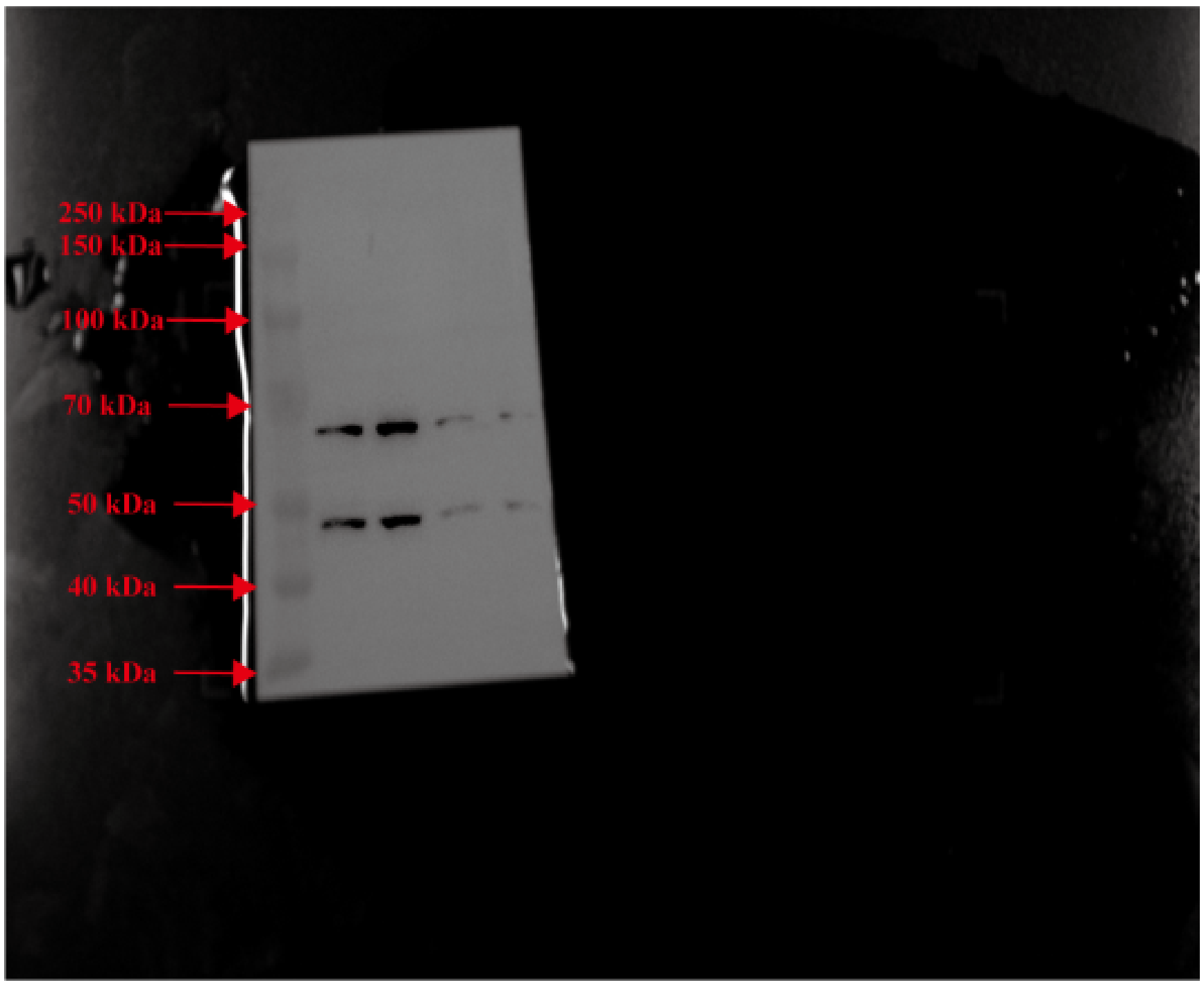

Tubulin

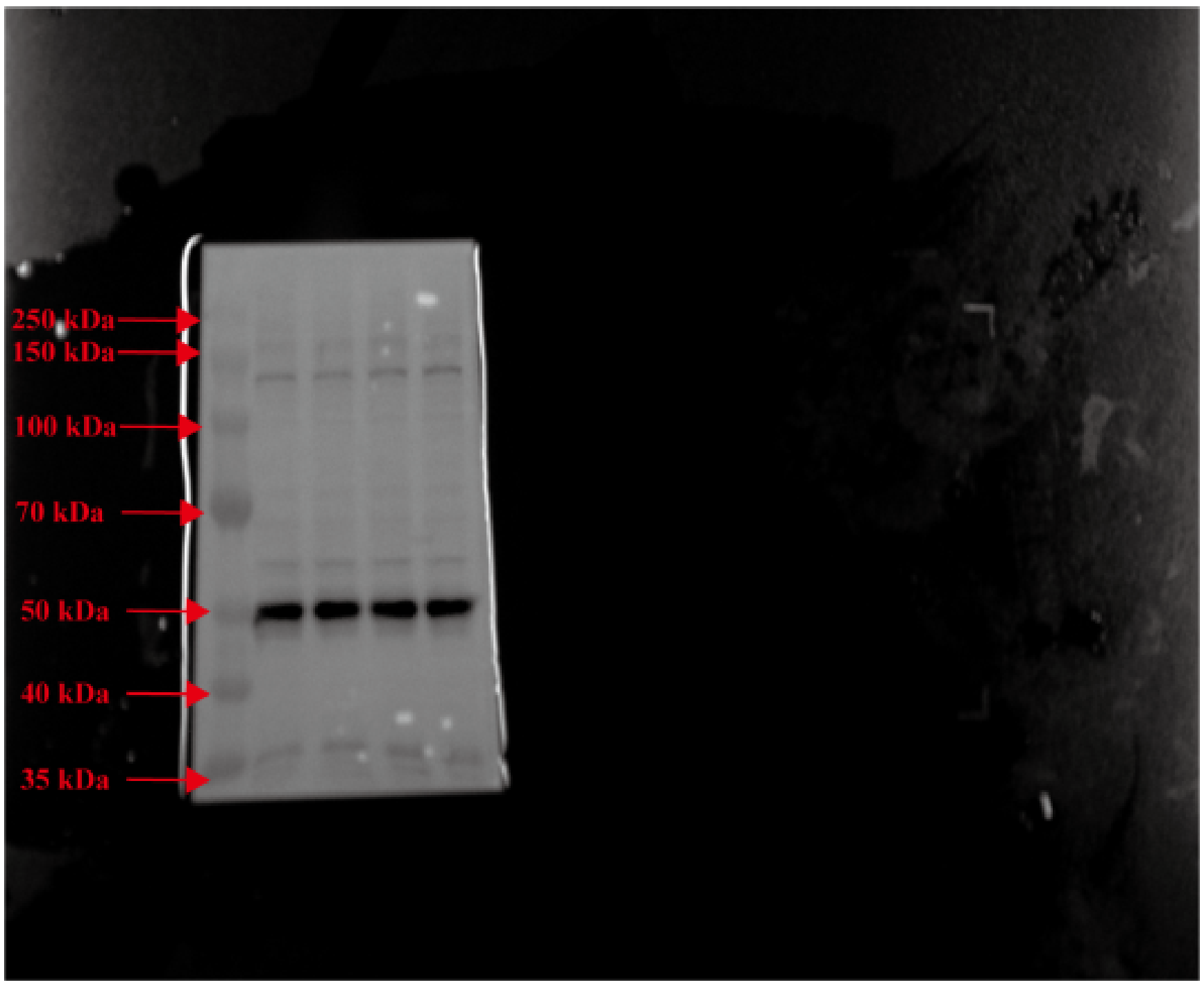

Fig 8K

Drebrin

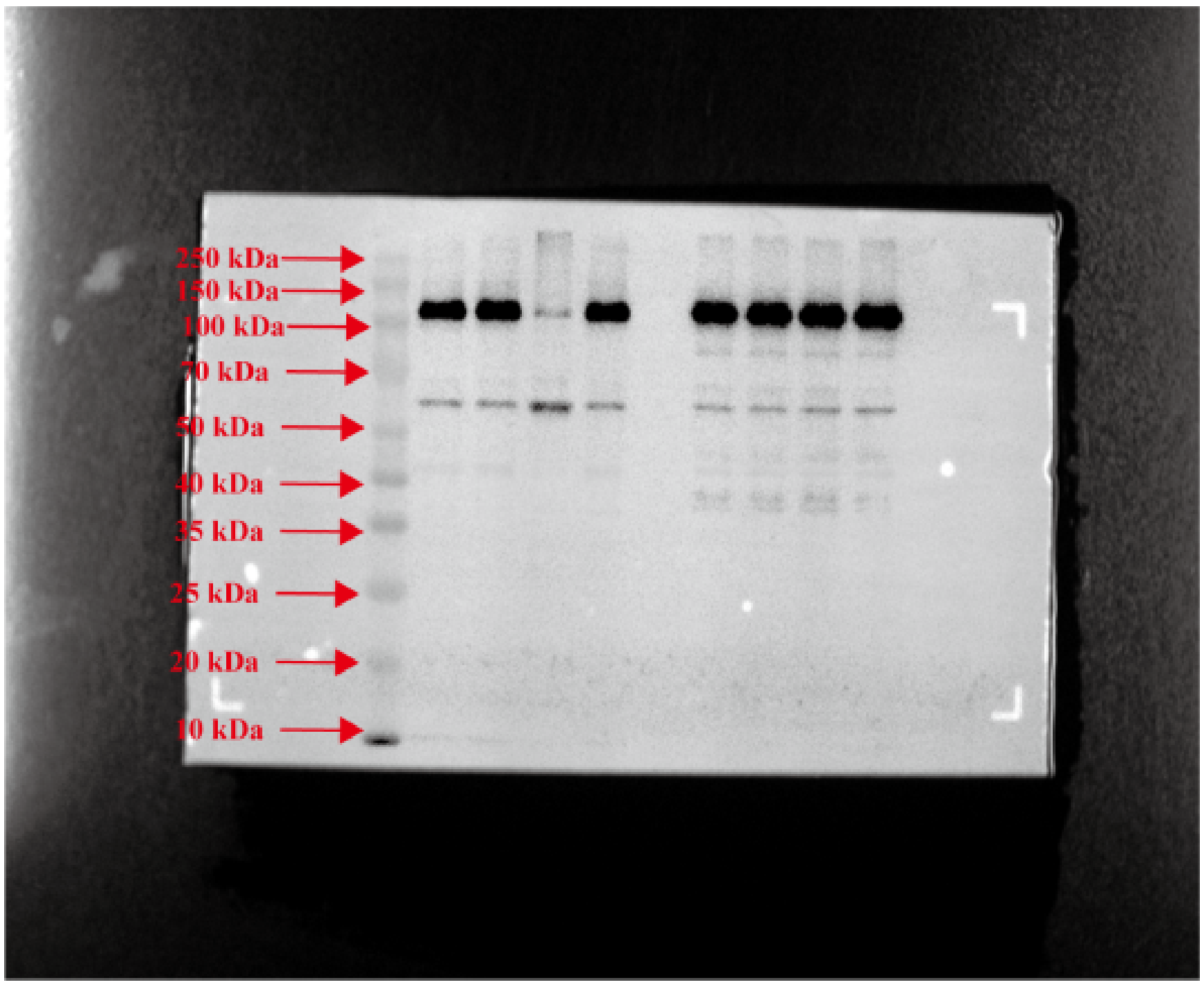

FLNA

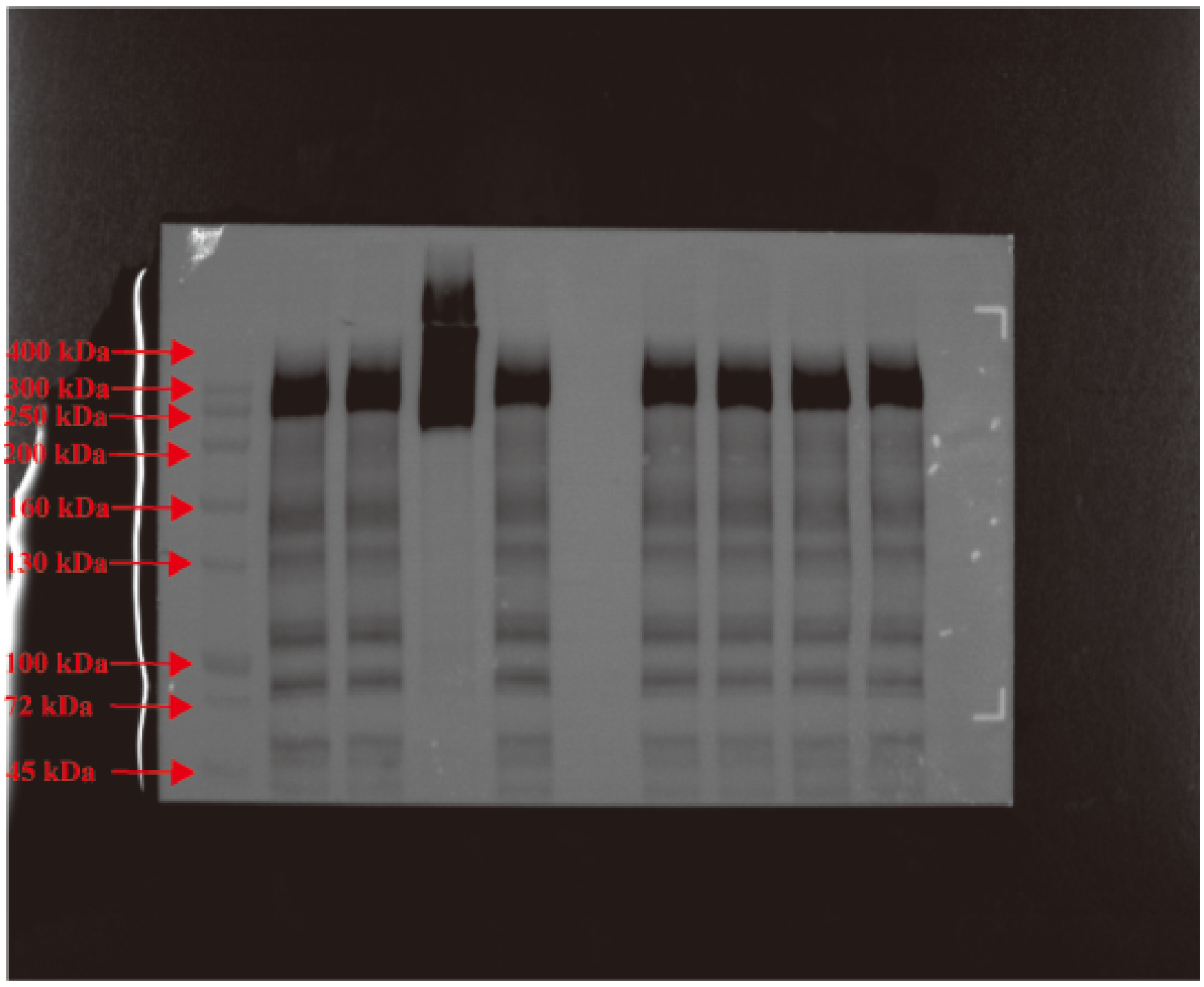
400 kDa
300 kDa
250 kDa
200 kDa
160 kDa
130 kDa
100 kDa
72 kDa
45 kDa

FLNB

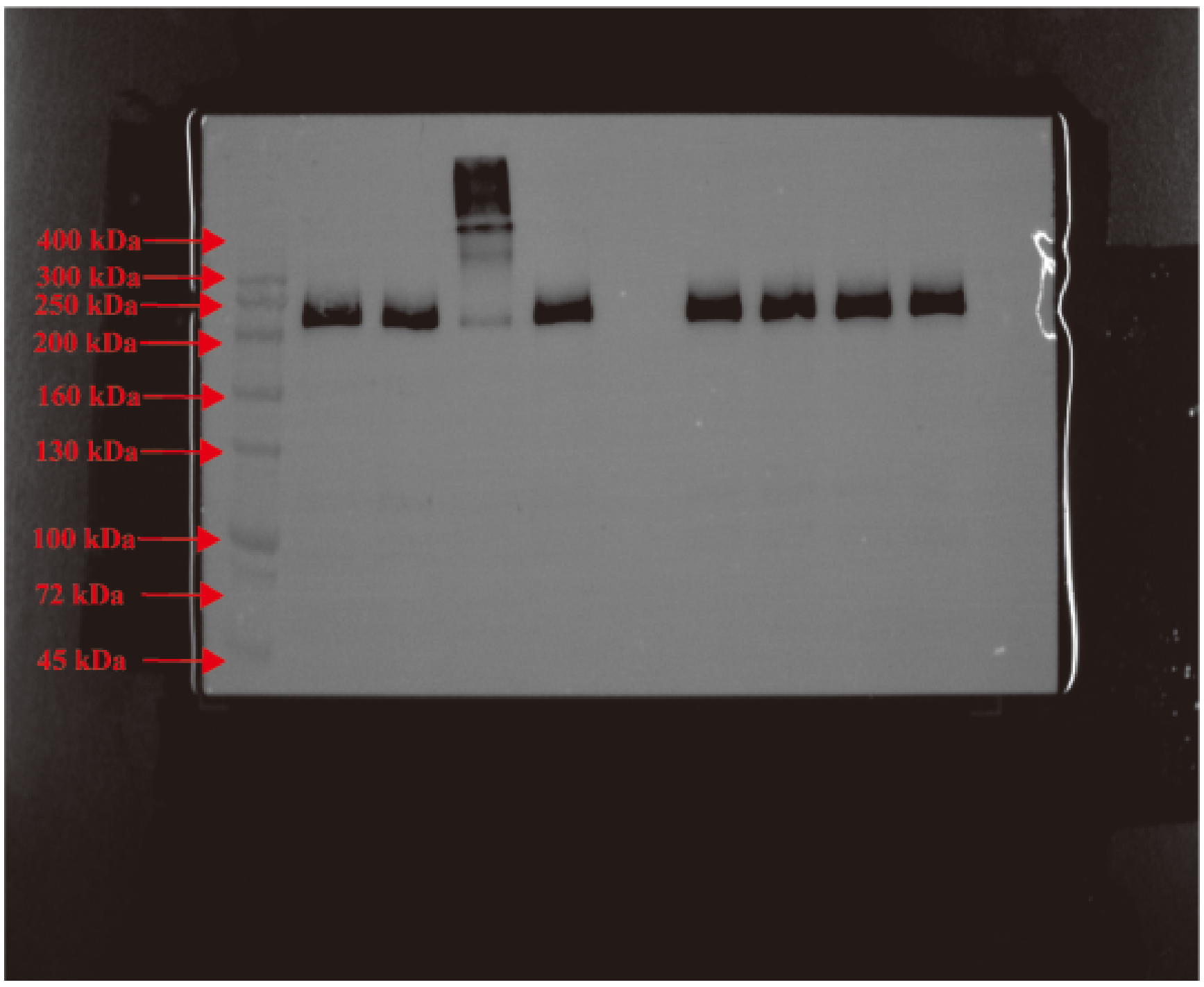
400 kDa
300 kDa
250 kDa
200 kDa
160 kDa
130 kDa
100 kDa
72 kDa
45 kDa

MYH9

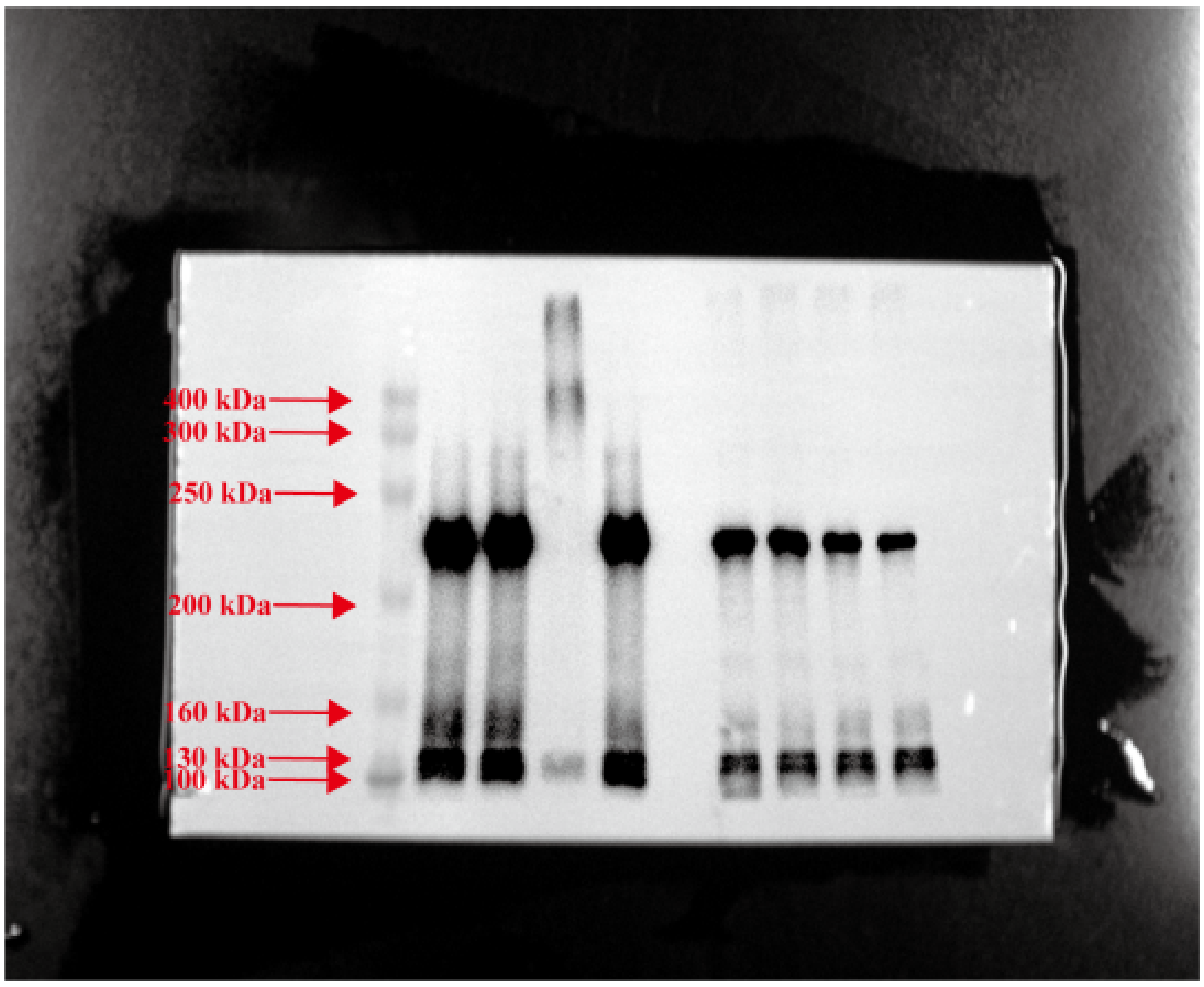

MYH10

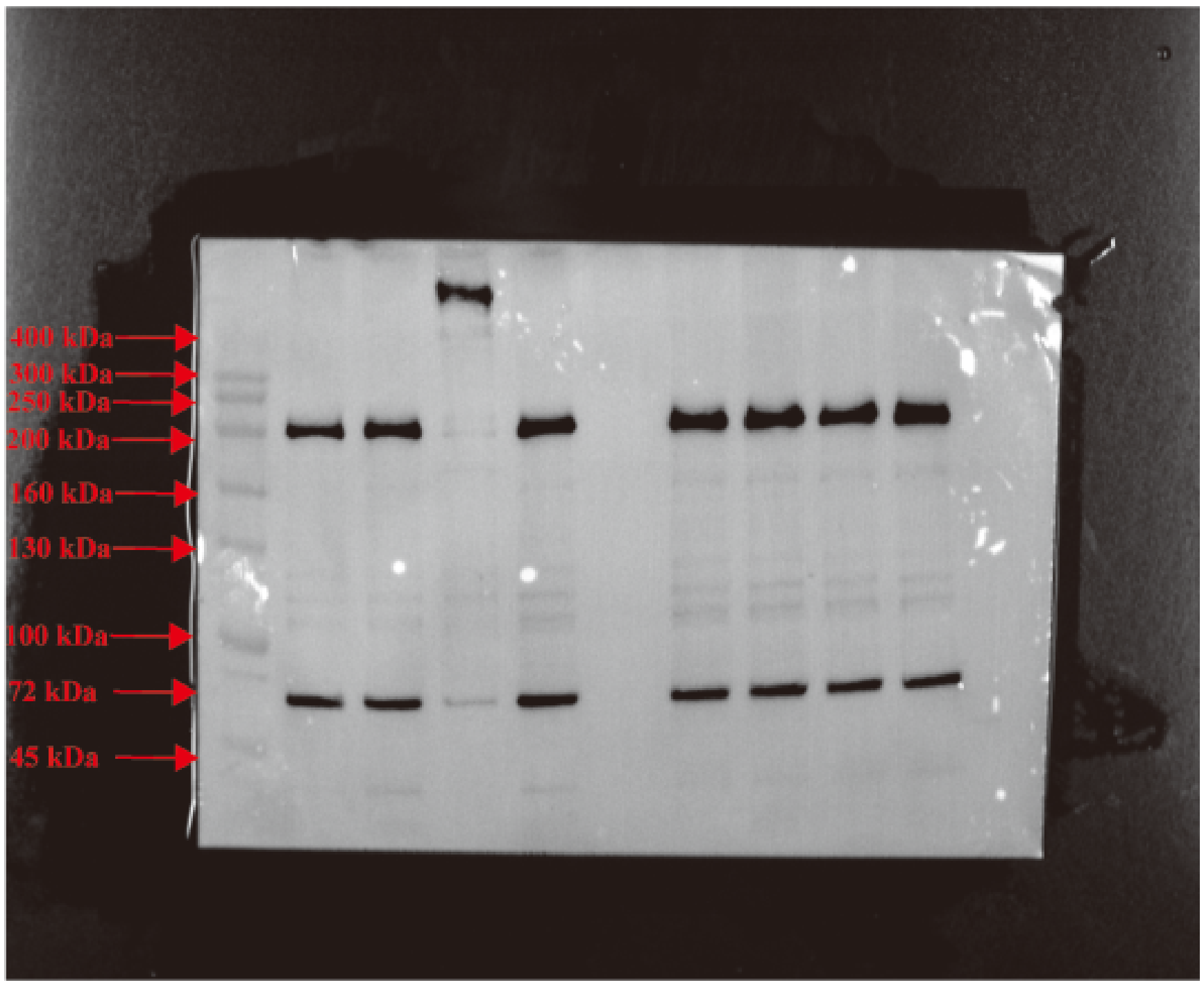

Fig S2 A

GAPDH

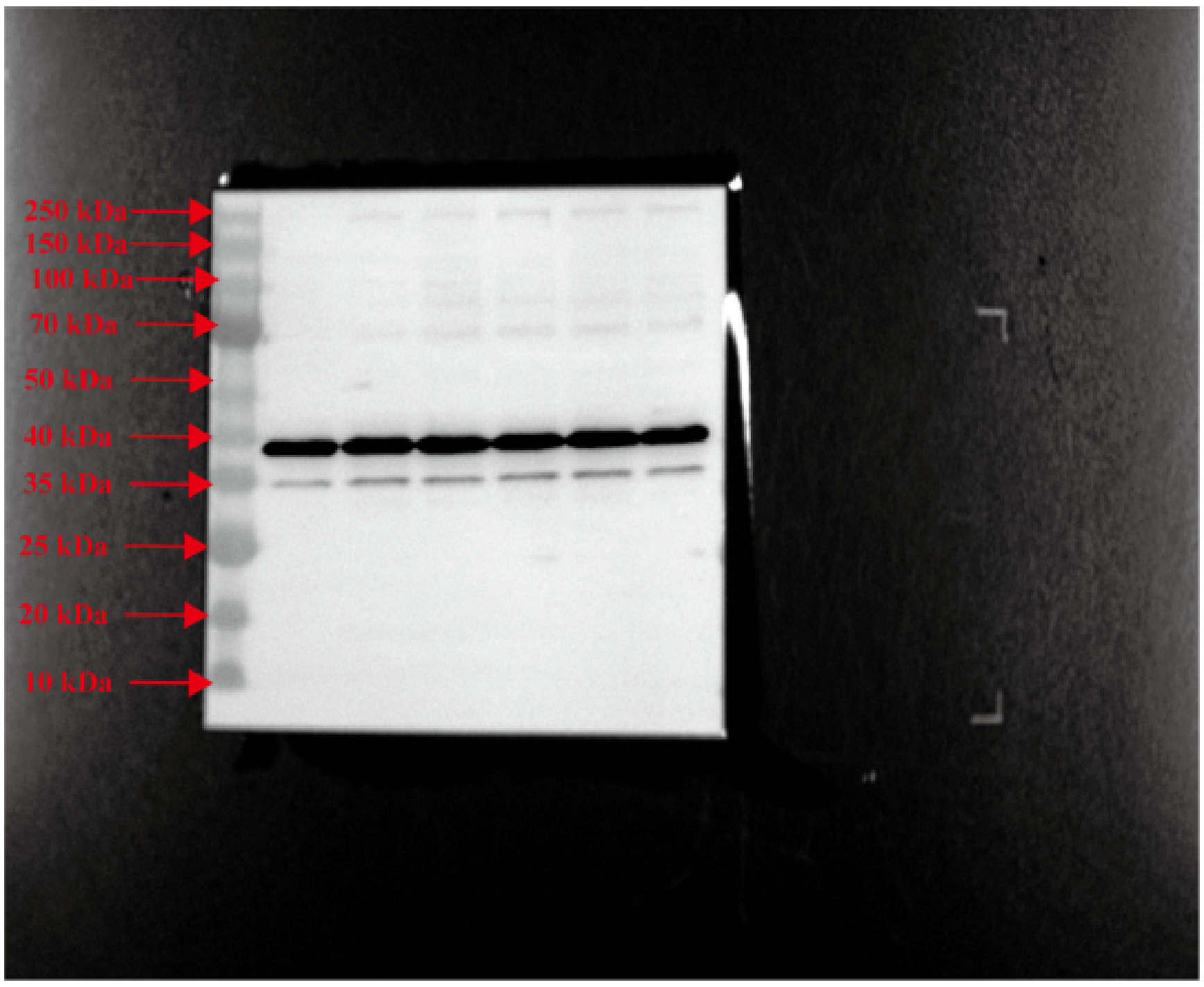

RBM41

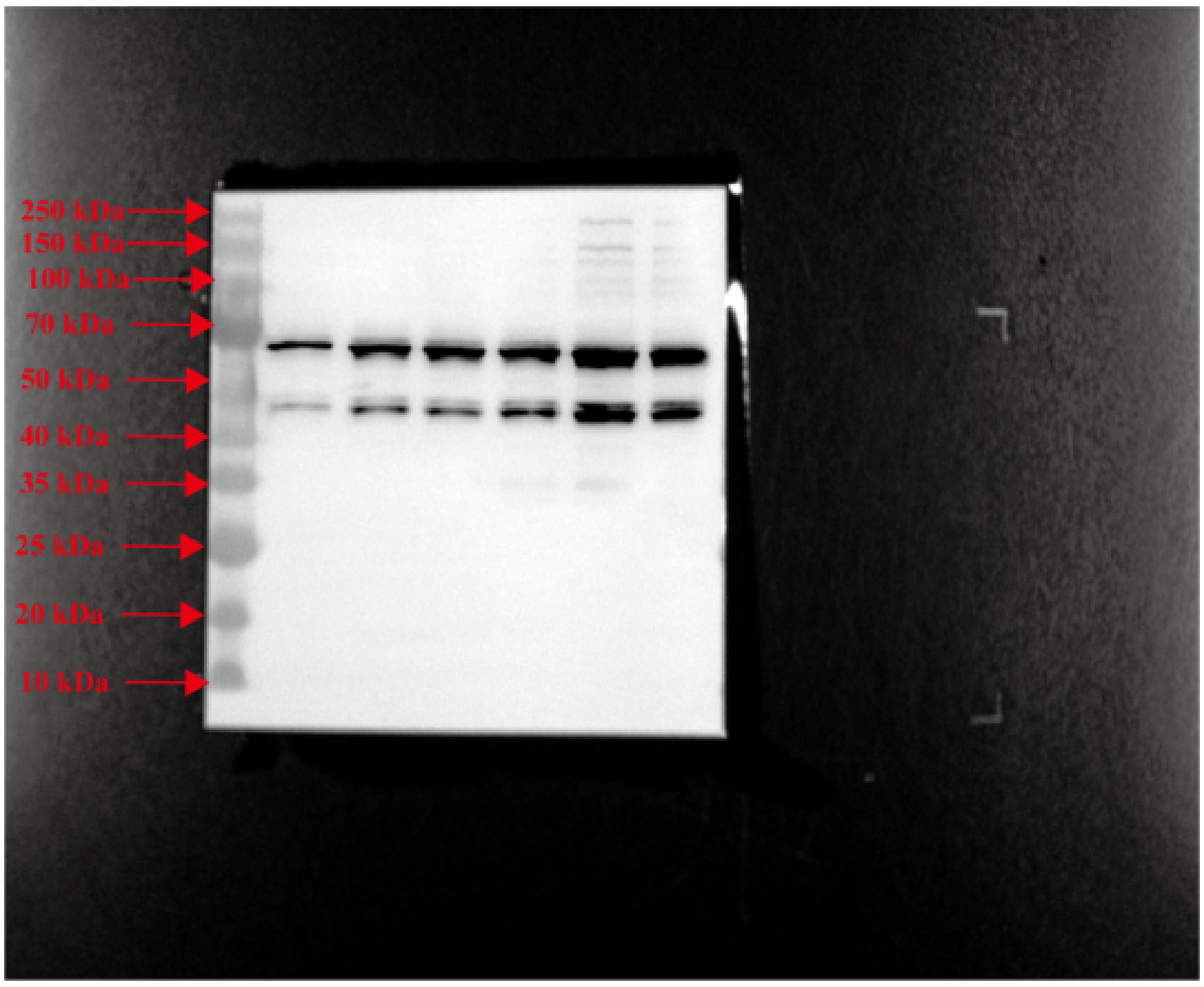

Fig S2 D

GAPDH

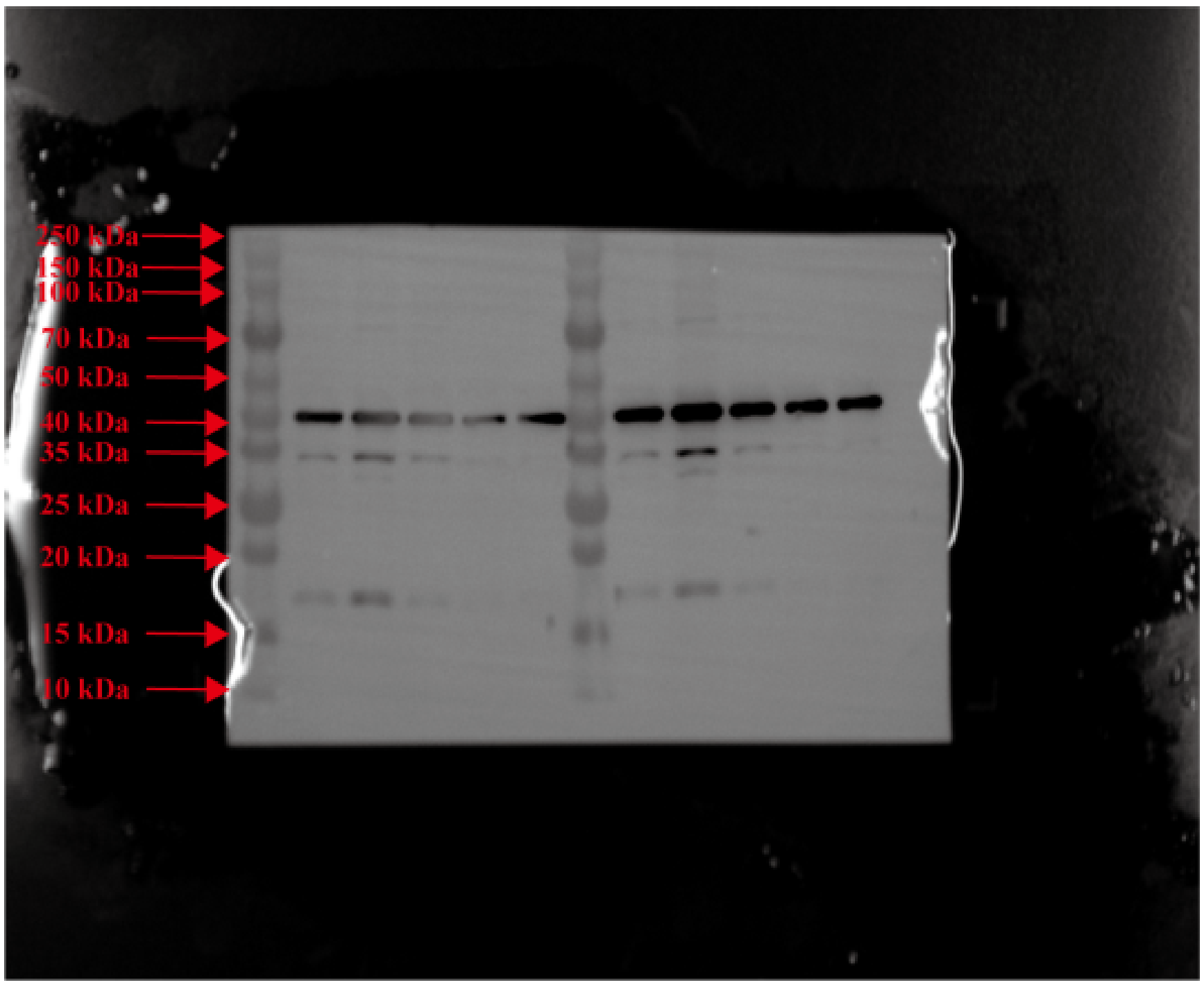

RBM41

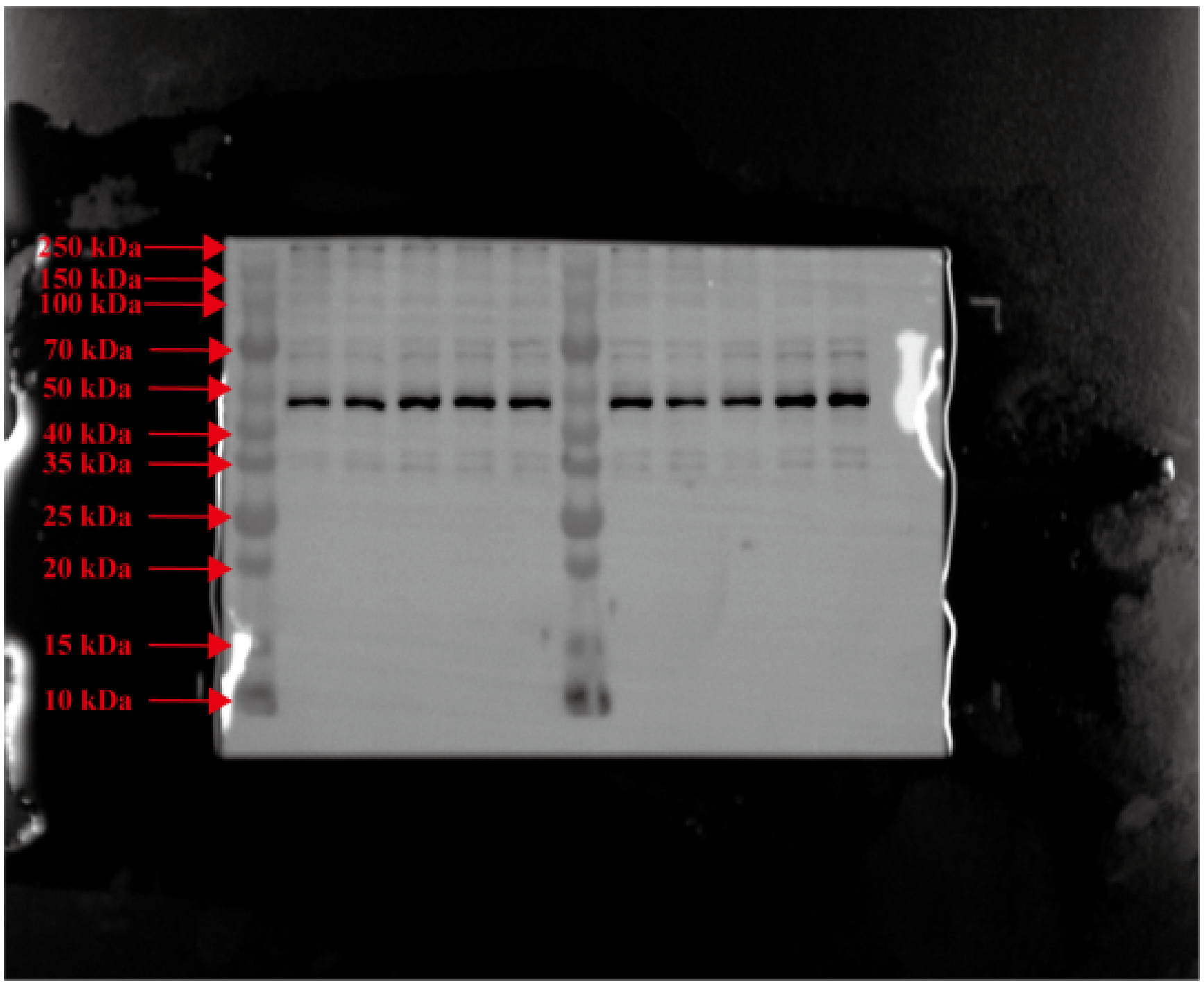

Fig S10A

GAPDH

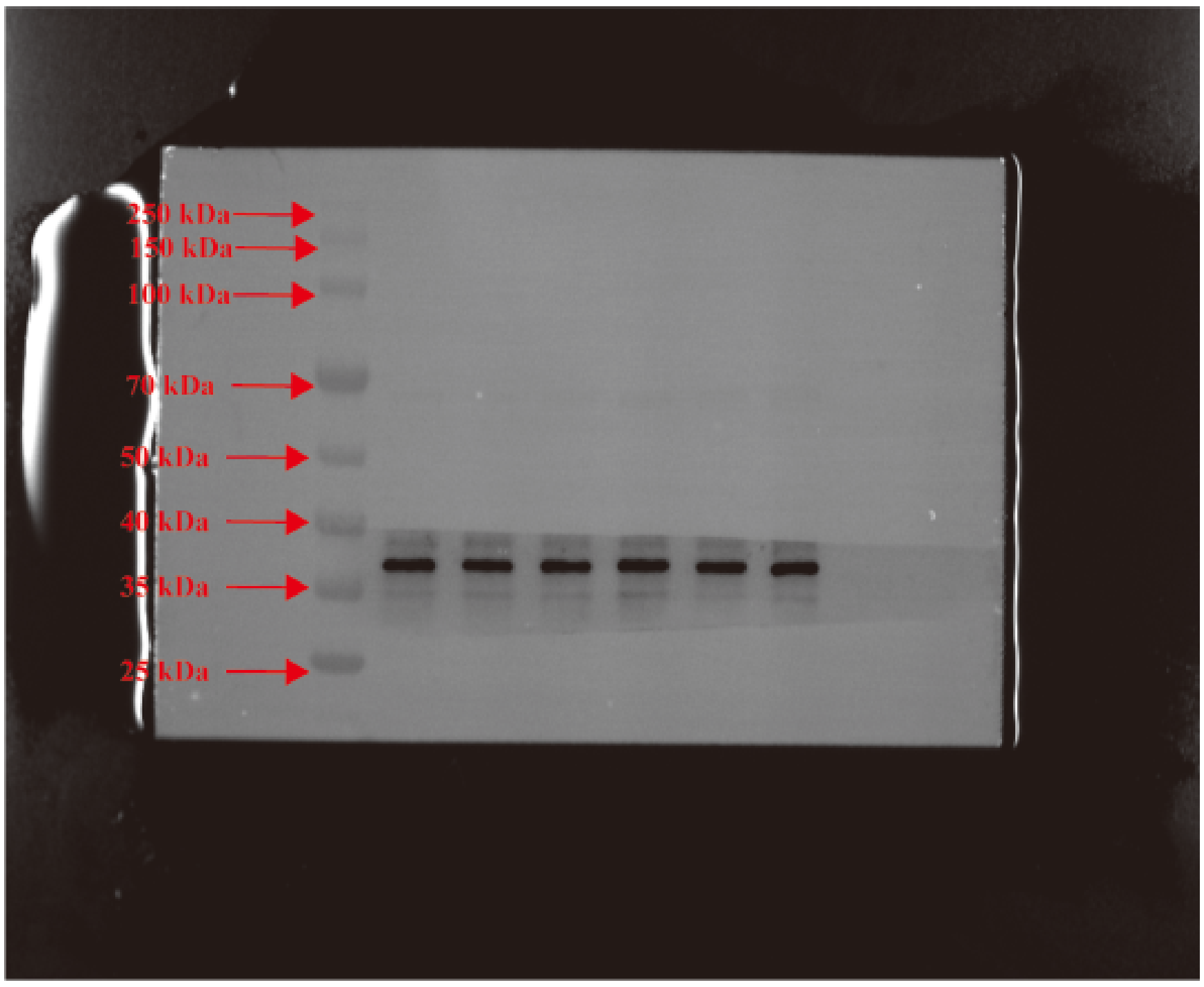

SLC7A11

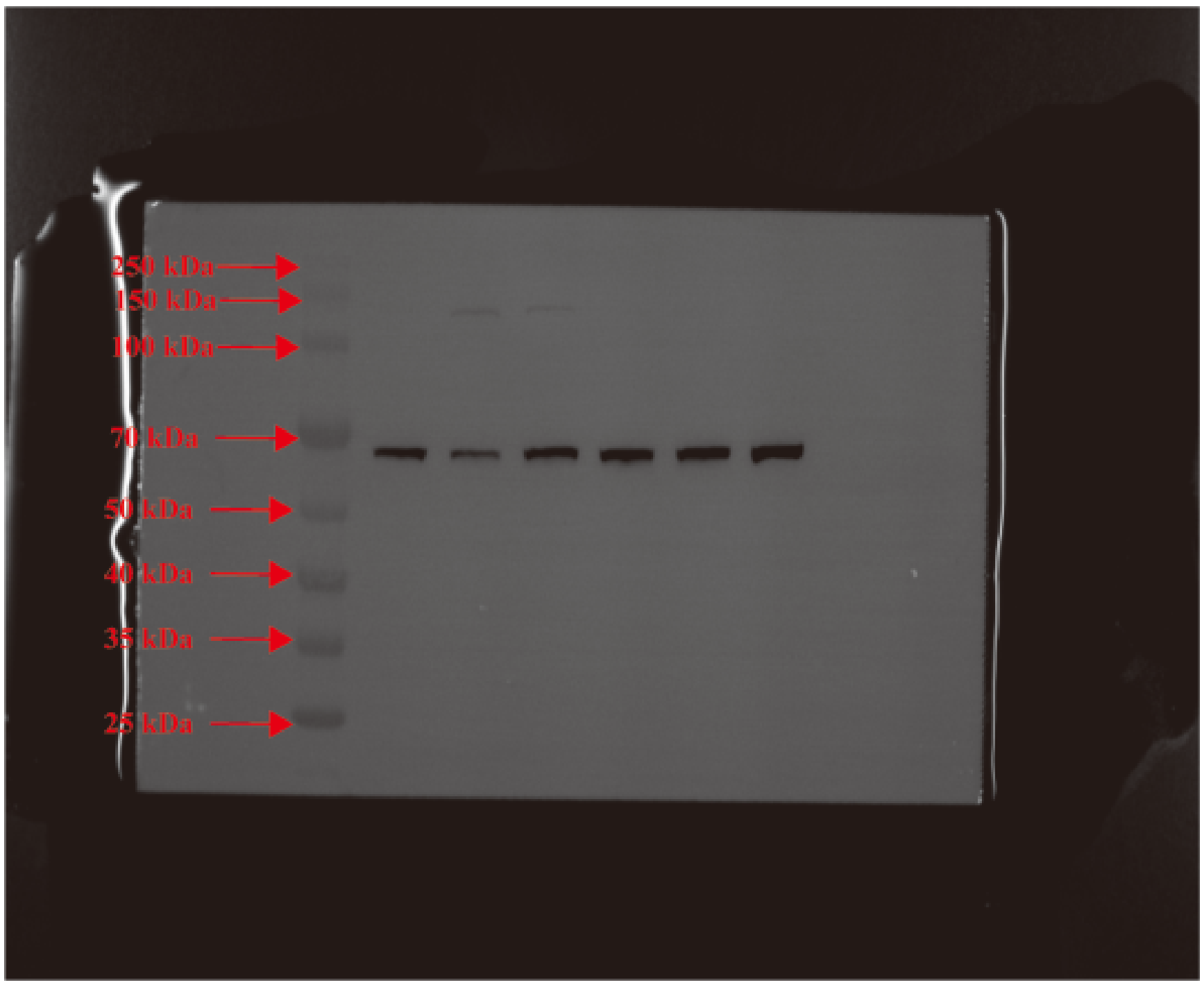

Fig S10F

GAPDH

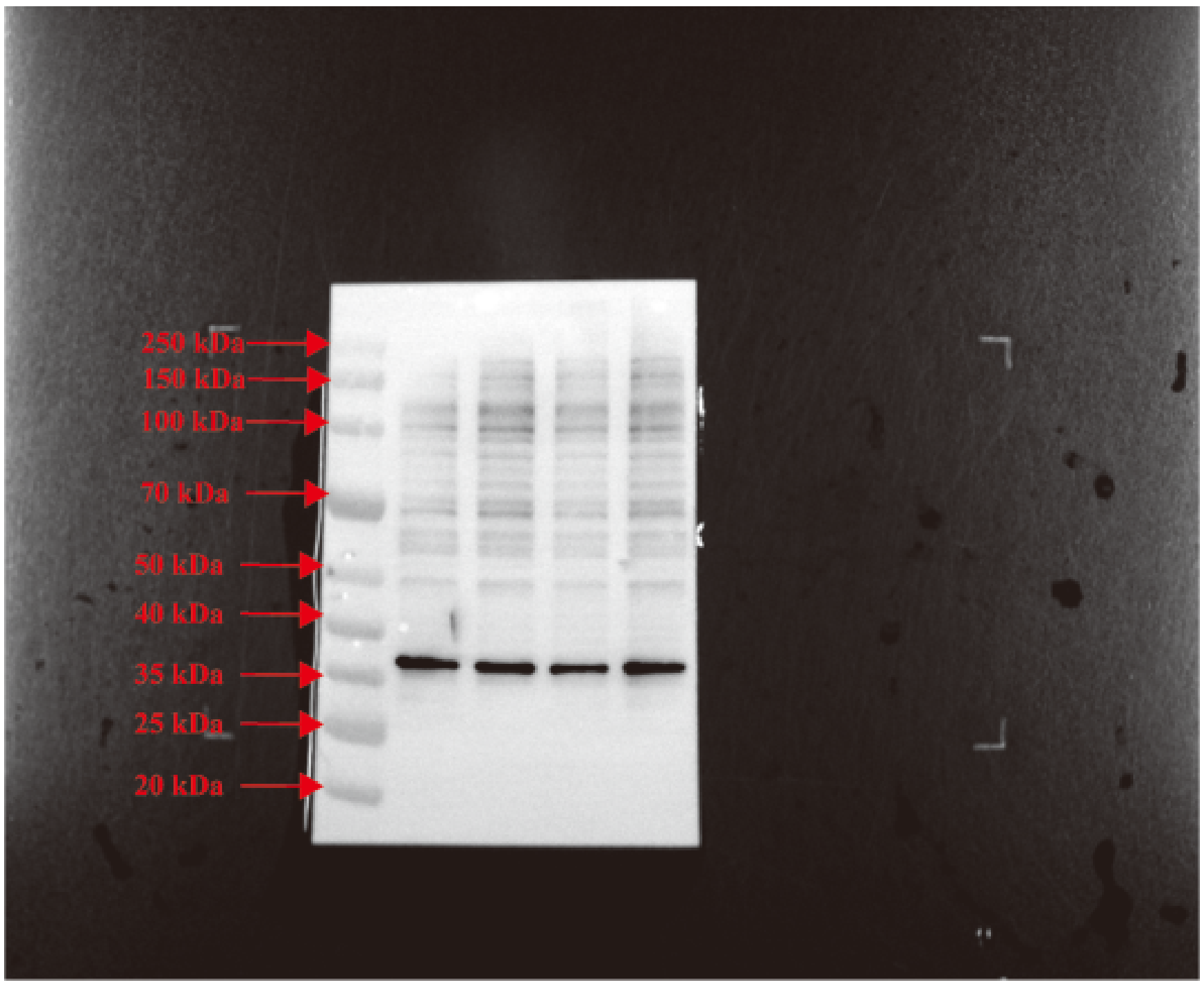

RBM41

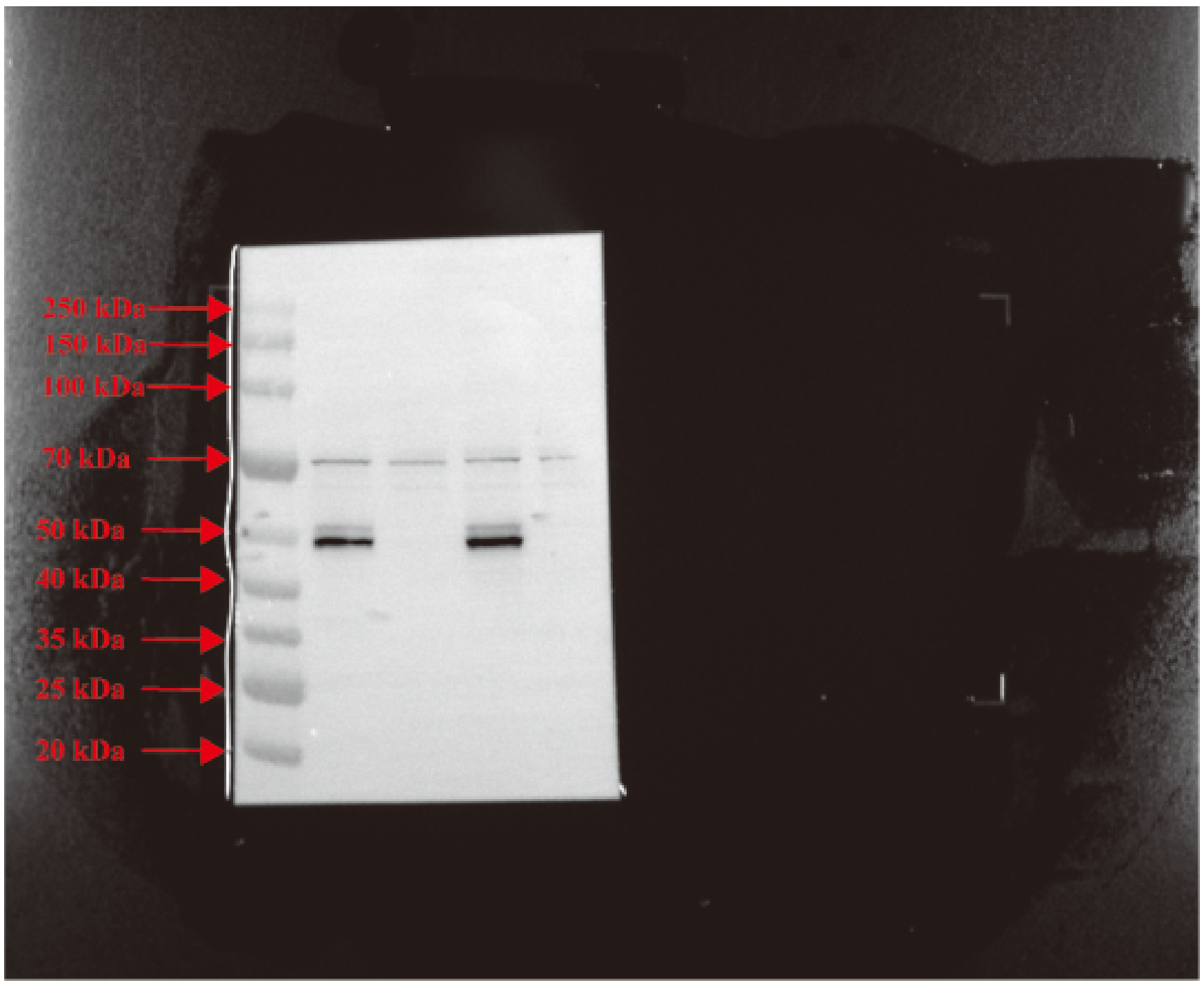

SLC7A11

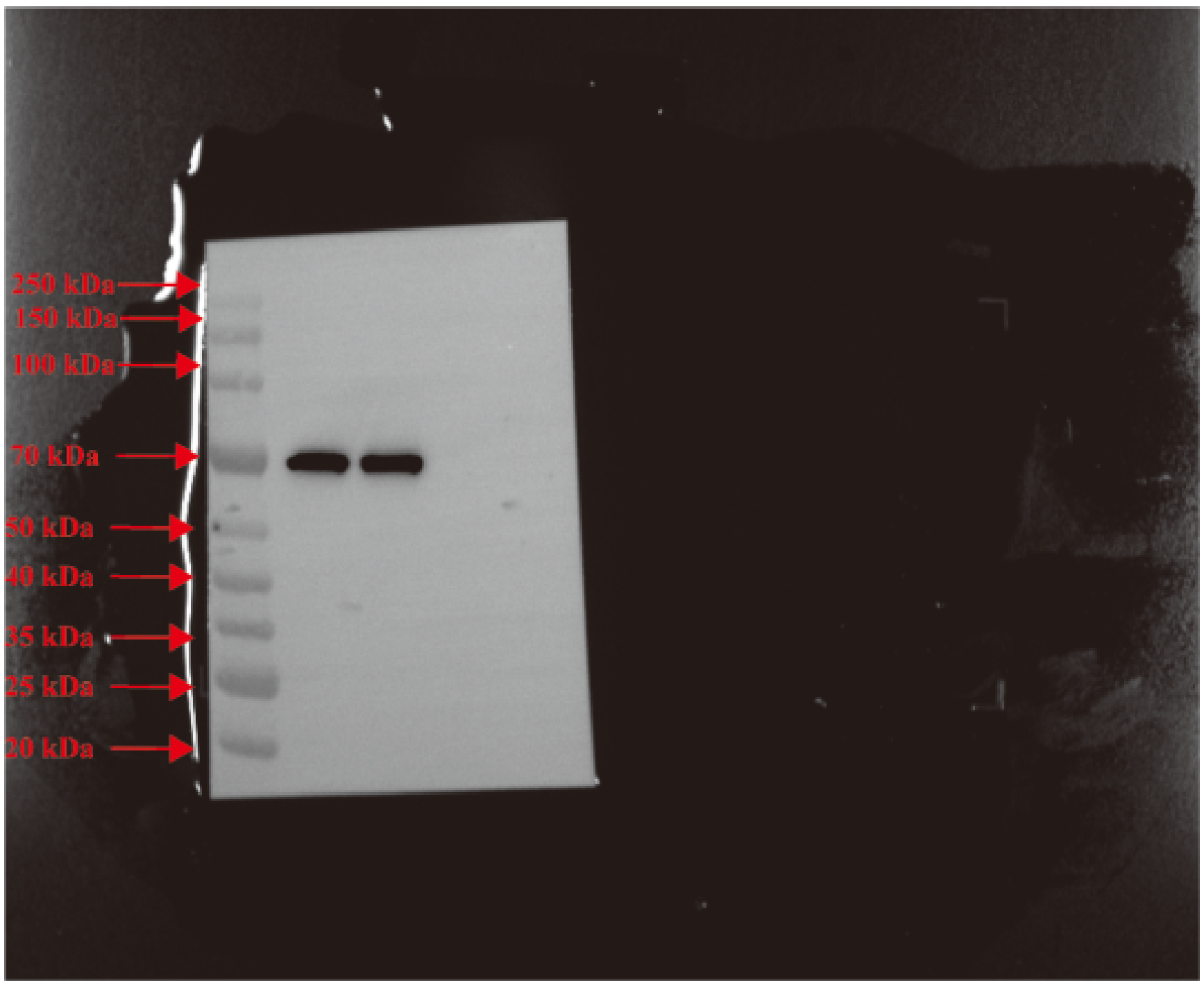

Fig S16

Tubulin

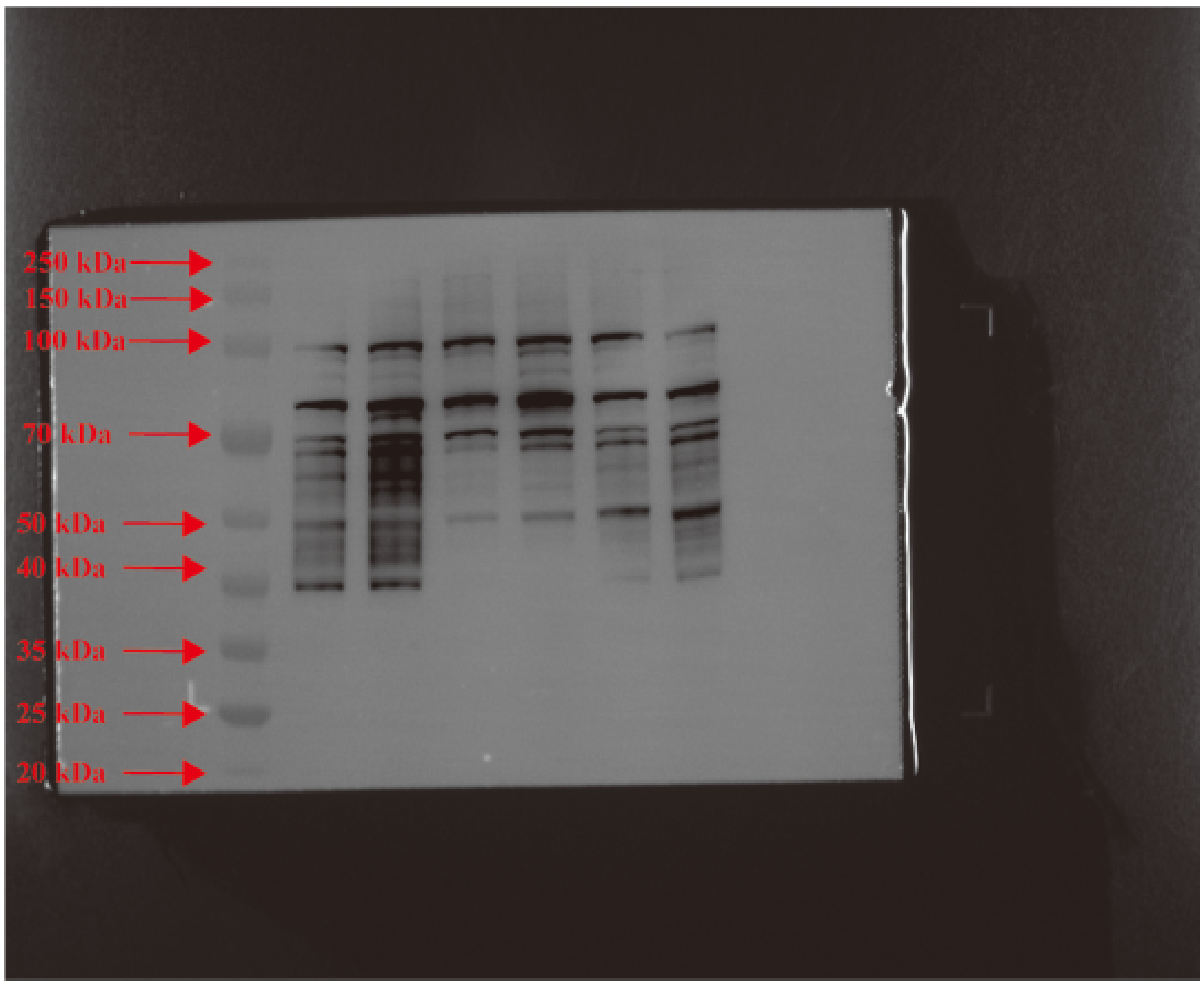

Tubulin

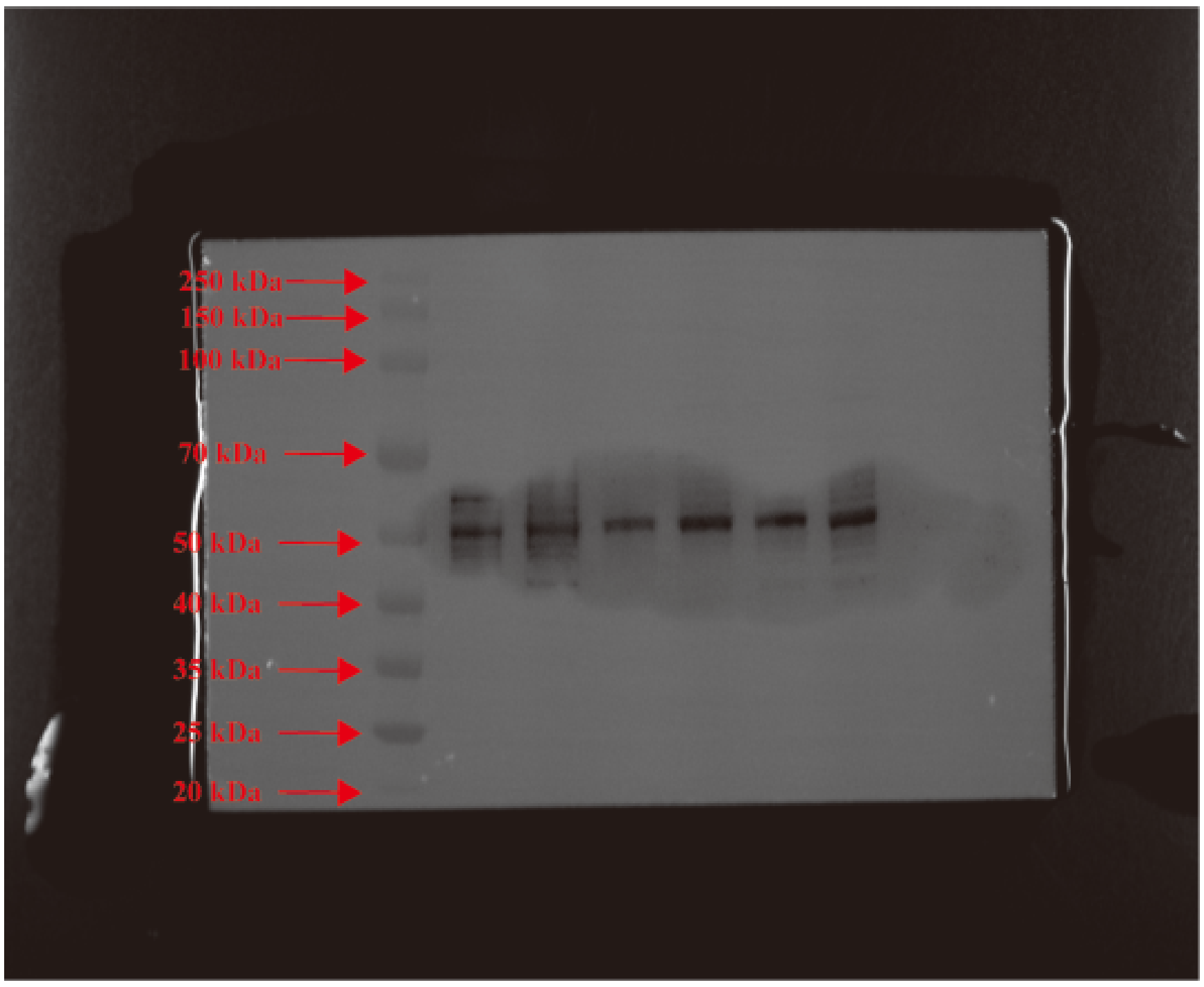

Fig S17D

CKMT1

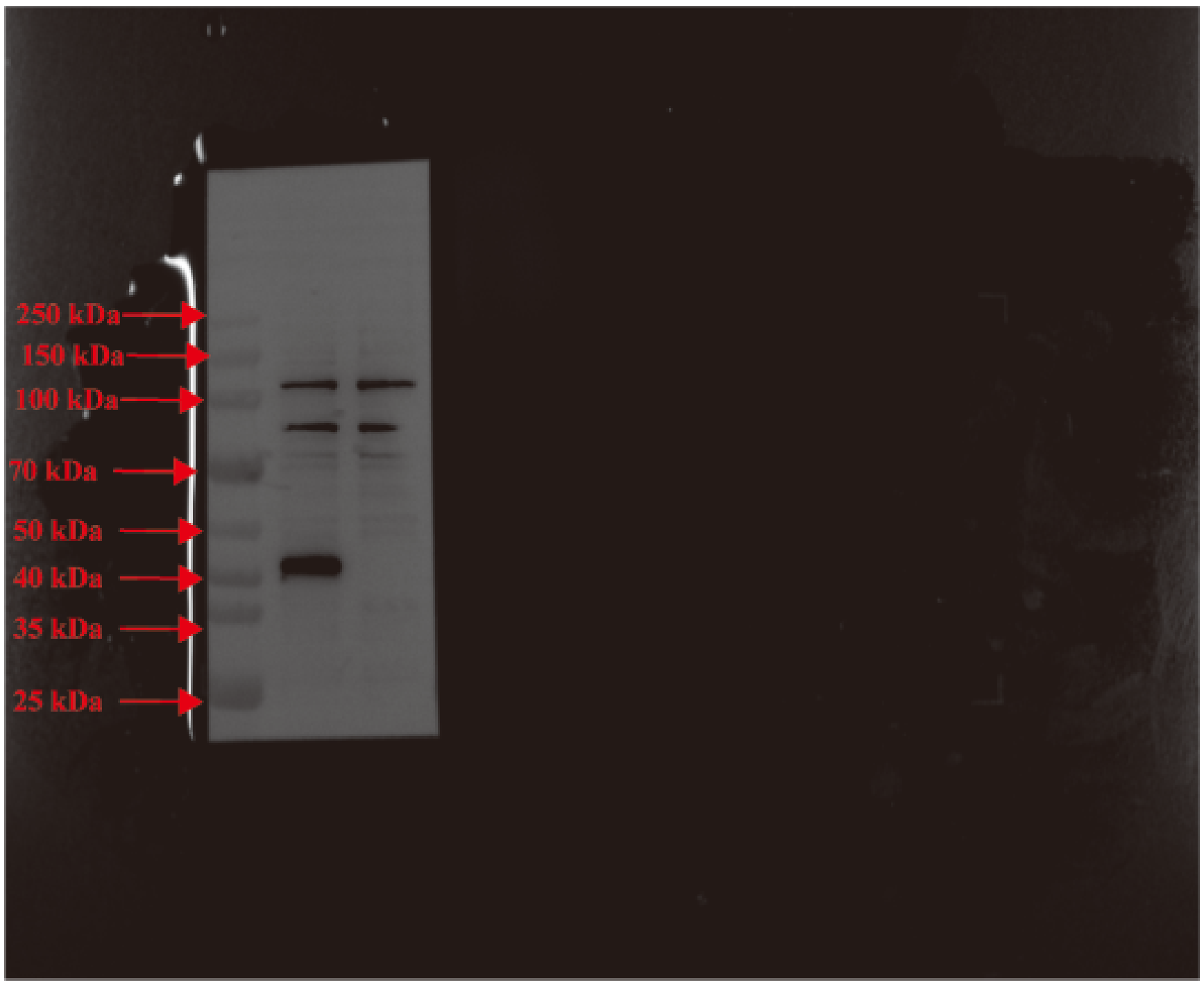

Tubulin

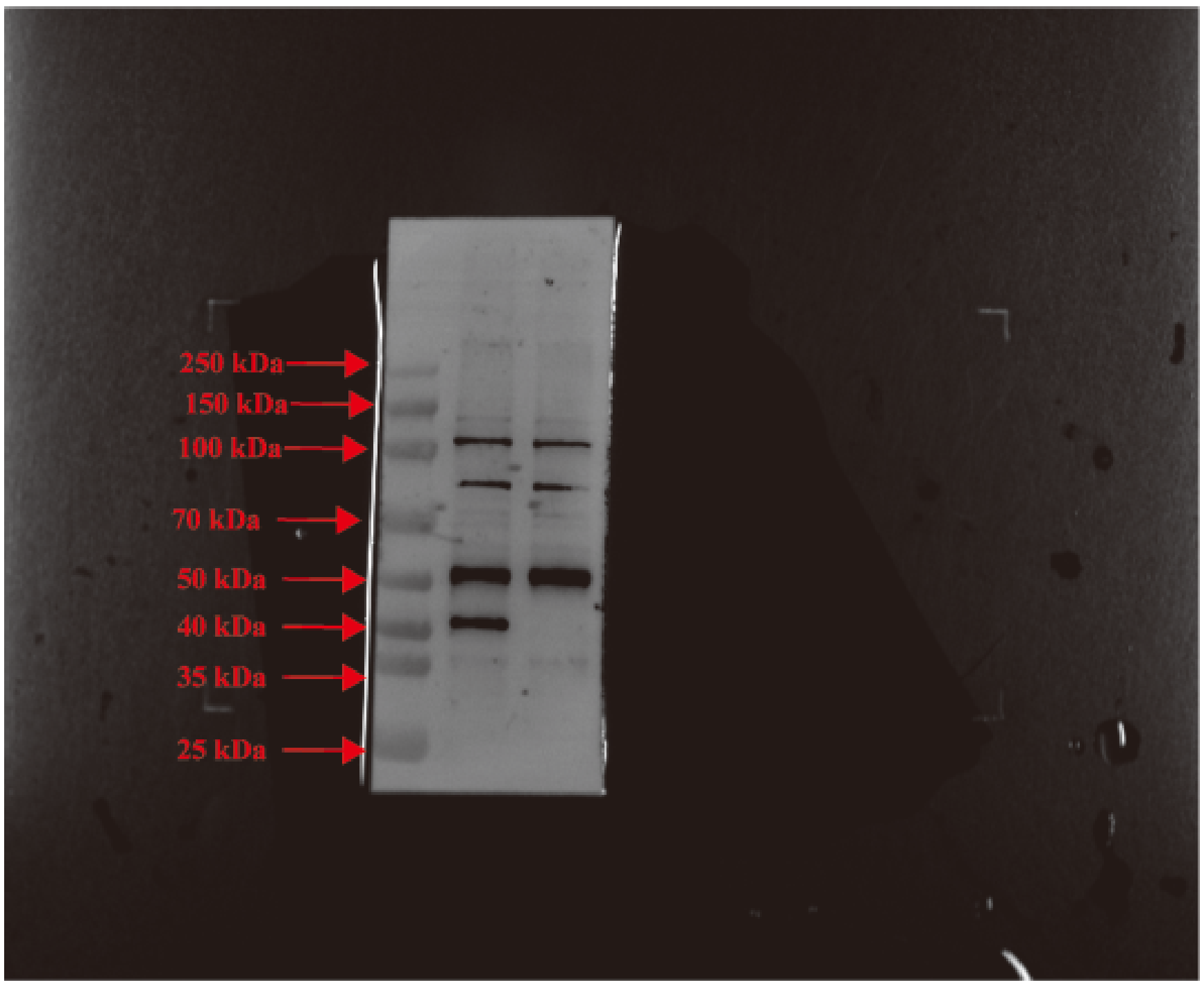

FigS17J
Drebrin

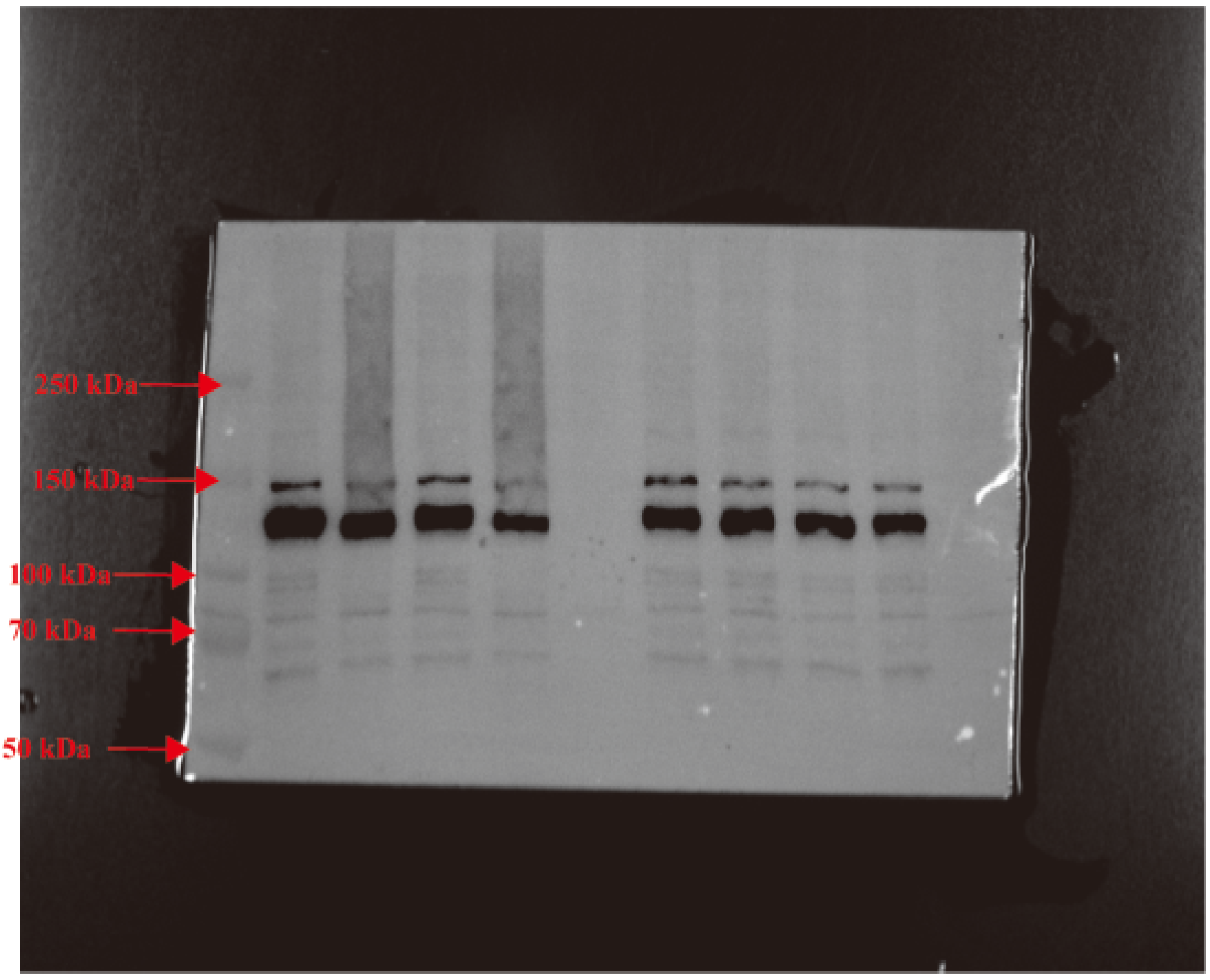

FLNA

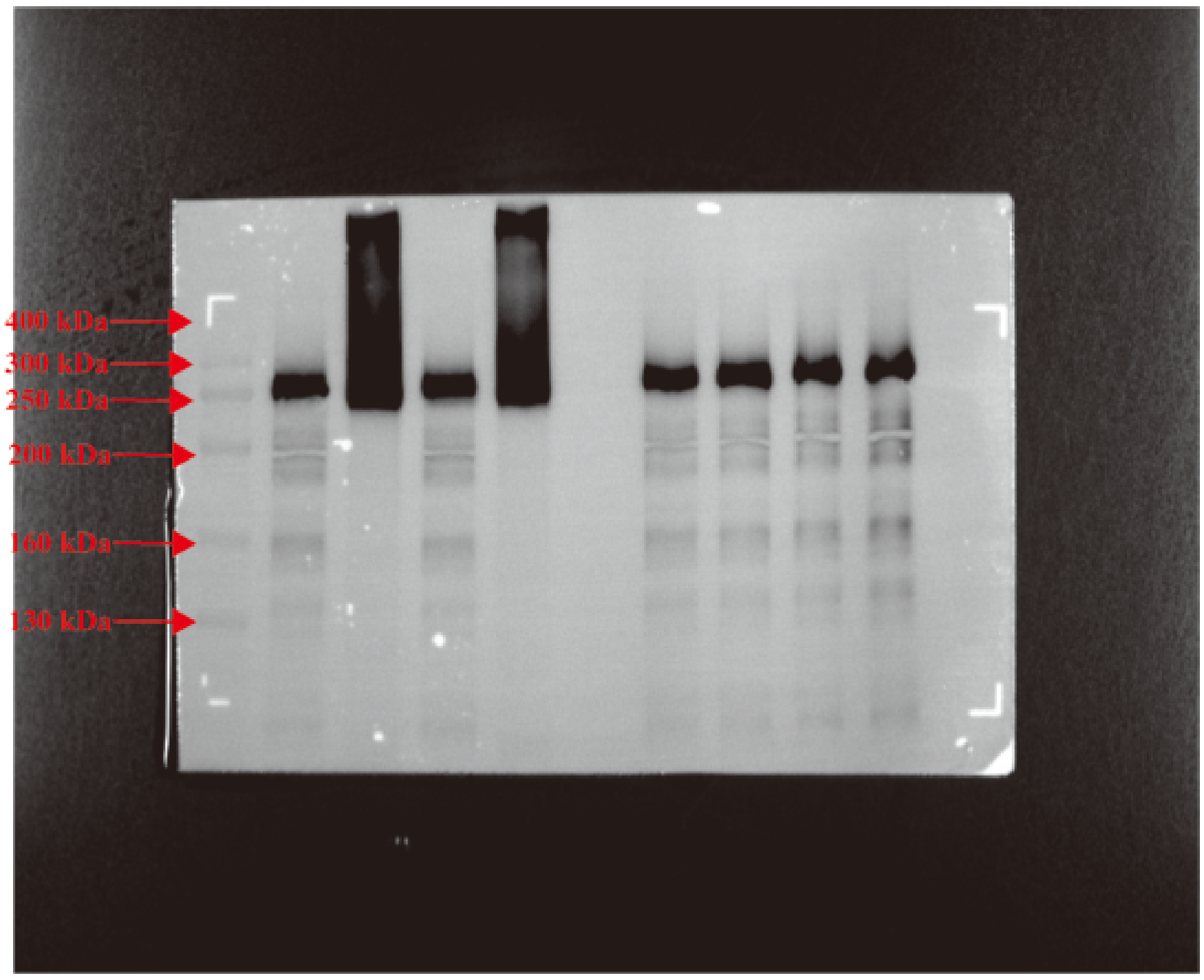
400 kDa
300 kDa
250 kDa
200 kDa
160 kDa
130 kDa

FLNB

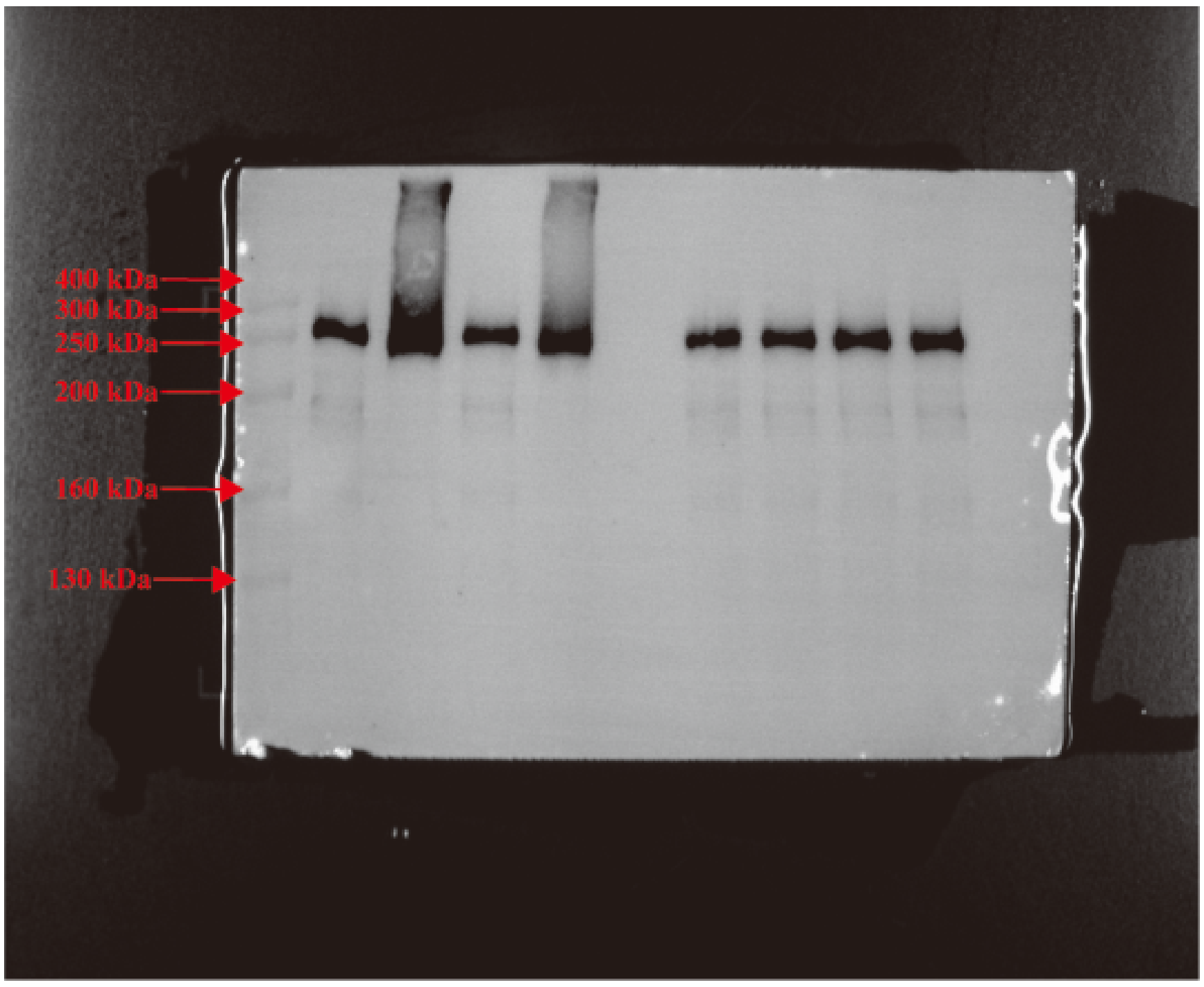
400 kDa
300 kDa
250 kDa
200 kDa
160 kDa
130 kDa

MYH9

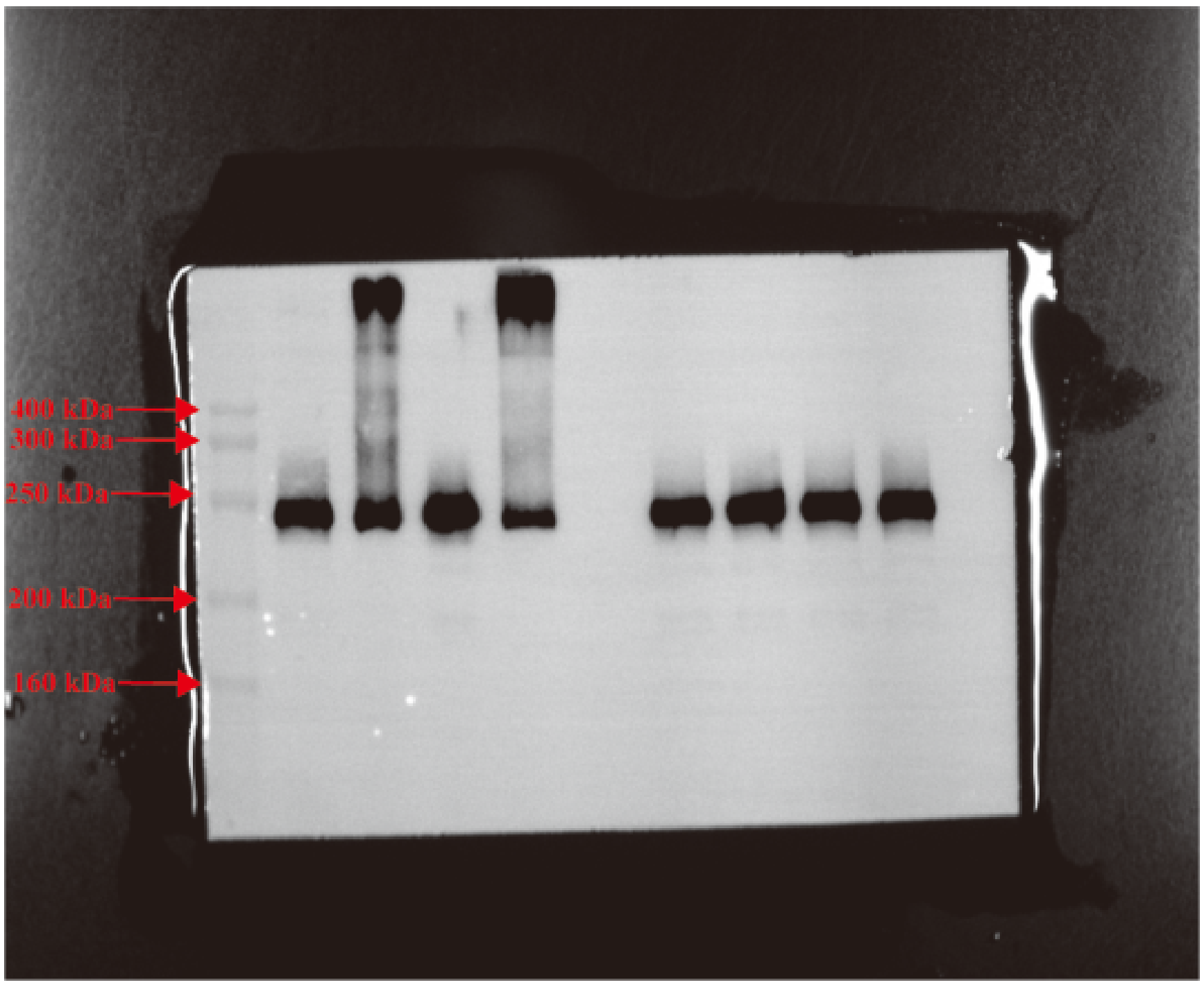
400 kDa
300 kDa
250 kDa
200 kDa
160 kDa

MYH10

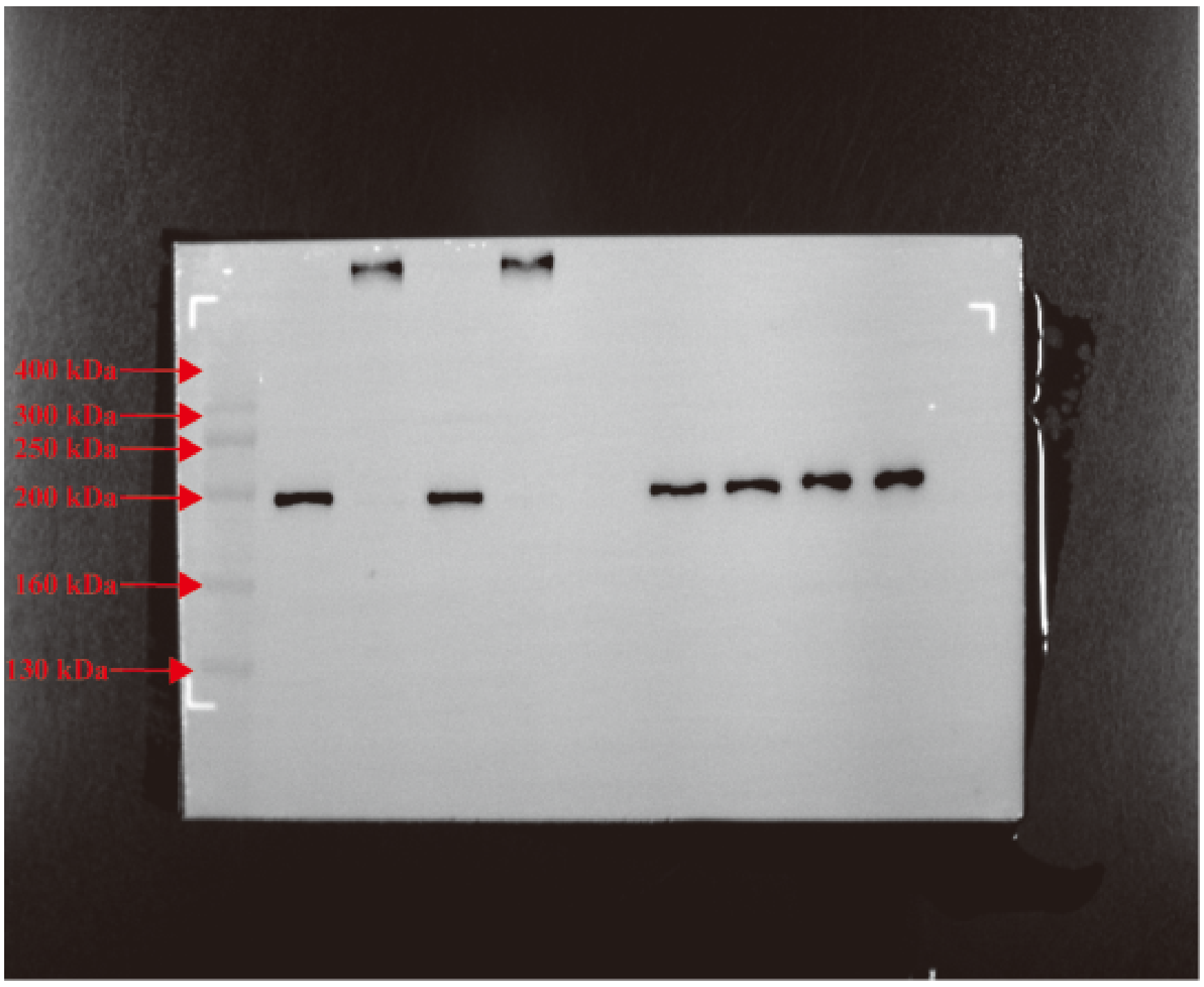
400 kDa
300 kDa
250 kDa
200 kDa
160 kDa
130 kDa
