# Supplementary material for: Targeting RBM41-mediated CKMT1B mRNA stability triggers disulfidptosis and suppresses tongue squamous cell carcinoma
Source: Cell Death Dis. 2026 Jul 31;17(1):914. doi: 10.1038/s41419-026-09166-2 (PMC13720023; doi:10.1038/s41419-026-09166-2)
Supplement: Supplementary file 2 [file 41419_2026_9166_MOESM1_ESM.docx]

**SUPPLEMENTARY INFORMATION**

**Targeting RBM41-mediated CKMT1B mRNA stability triggers disulfidptosis and suppresses tongue squamous cell carcinoma**

Boqiang Tao^1,3^, Ming Hao^1,3^, Yang Hao^2^, Shuhan Zhang^2^, Zhaorong Xiao^1,3^, He Shi^1, 3^, Qirong Li^2^, Yiwen Qin^1,3^, Dongxu Wang^2,^*, Weiwei Liu^1,3,^*

^1^Department of Oral and Maxillofacial Surgery, Hospital of Stomatology, Jilin University, Changchun, Jilin, China

^2^ Laboratory Animal Center, College of Animal Science, Jilin University, Changchun, Jilin, China

^3^ Jilin Provincial Key Laboratory of Oral Biomedical Engineering, Hospital of Stomatology, Jilin University, Changchun, Jilin, China

* Correspondence

*These authors contributed equally to this work

**Supplementary Figure 1**

**Supplementary Figure 1. Kaplan-Meier overall survival analysis of RBM41 and CKMT1B expression.**

Kaplan-Meier curves show overall survival probability (y-axis) as a function of time in days (x-axis), separated into low and high expression subgroups for RBM41 (A) and CKMT1B (B). Log-rank test-derived hazard ratios (HR), 95% confidence intervals, and P values are annotated on each plot. RBM41: HR = 1.26 (0.92–1.75), P = 0.187; CKMT1B: HR = 1.26 (0.89–1.79), P = 0.154.

**Supplementary Figure 2**

**Supplementary Figure 2. The expression of RBM41 in various cell lines.**

(A, B) Western blot analysis of RBM41 protein levels in normal cell lines (293T, HOK, HOEC) and TSCC cell lines (Cal-27, SCC-9, SCC-15). (C - E) qRT-PCR (C) and Western blot (D, E) analysis confirming the efficiency of RBM41 knockdown (siRBM41) and overexpression (OE) in SCC-15 cells. n=3 biological replicates. Error bars represent mean ± SD. Significance was determined using a one-way ANOVA with Tukey's multiple comparisons test (B, C, E).

**Supplementary Figure 3**

**Supplementary Figure 3. RBM41 modulates the cell proliferation and apoptosis of SCC-9 cells** **under - Glc conditions.**

(A) CCK-8 assay measuring cell viability of WT, RBM41 KO, and RBM41 OE cells under glucose deprivation (- Glc) over time, n = 6 biological replicates. (B, C) Flow cytometry analysis (B) and quantification (C) of cell death after 9 hours of - Glc treatment, n = 3 biological replicates.

Error bars represent mean ± SD. Significance was determined using a two-way ANOVA with Tukey's multiple comparisons test (A, C). P values are as indicated.

**Supplementary Figure 4**

**Supplementary Figure 4. Cell viability and apoptosis analysis of WT and RBM41 KO SCC-15 cells under serum (-FBS) or glutamine (-Gln) starvation.**

(A) CCK-8 assay measuring cell viability of WT and RBM41 KO SCC-15 cells under -Gln (left) and-FBS (right) over time, n = 6 biological replicates. (B, C) Flow cytometry analysis (B) and quantification (C) of cell death after 24 hours of -Gln (left) and -FBS (right) in WT, RBM41 KO cell lines, n = 3 biological replicates.

Error bars represent mean ± SD. Significance was determined using a two-way ANOVA with Šídák's multiple comparisons test (A, C). P values are as indicated.

**Supplementary Figure 5**

**Supplementary Figure 5. RBM41 modulates the glucose uptake capacity of SCC-9 cells and the ATP level under - Glc conditions.**

(A) Glucose uptake assay in WT, RBM41 KO, and RBM41 OE cells, n = 3 biological replicates. (B) Intracellular ATP levels in WT, RBM41 KO, and RBM41 OE cell lines under normal and - Glc conditions, n = 3 biological replicates.

Error bars represent mean ± SD. Significance was determined using a one-way ANOVA with Dunnett's multiple comparisons test (A, B). P values are as indicated.

**Supplementary Figure 6**

**Supplementary Figure 6. RBM41 can not modulates the cystine uptake ability under – Glc in TSCC cell.**

(A) Cystine uptake activity in SCC-15 WT, RBM41 KO, and RBM41 OE cells under + Glc and - Glc conditions, n = 3 biological replicates. (B) Cystine uptake activity in SCC-9 WT, RBM41 KO, and RBM41 OE cells under + Glc and - Glc conditions, n = 3 biological replicates.

Error bars represent mean ± SD. Significance was determined using a two-way ANOVA with Šídák's multiple comparisons test (A, B). P values are as indicated.

**Supplementary Figure 7**

**Supplementary Figure 7. The effects of various cell death inhibitors on cell death in RBM41 KO cells under - Glc conditions.**

(A, B) Flow cytometry dot plots showing Annexin V/PI staining in cells treated with various inhibitors (CQ, Z-VAD, Nec-1, Fer-1, 2DG, DTT,β-ME) under - Glc condition. (C) Flow cytometry analysis quantification of (H), flow cytometry analysis n = 3 biological replicates. (D) Cell viability and mortality of RBM41 KO cells treated with – Glc and various cell death inhibitors for 9 hours were measured using the CCK-8 assay. CCK-8 assay n = 6 biological replicates.

Error bars represent mean ± SD. Significance was determined using a one-way ANOVA with Dunnett's multiple comparisons test (C, D). P values are as indicated.

**Supplementary Figure 8**

**Supplementary Figure 8. RBM41 modulates reactive oxygen species production, and glutathione redox balance under – Glc in TSCC cell.**

(A) NADP+/NADPH ratio in WT, RBM41 KO, and RBM41 OE cell lines under -Glc 4 hours, n = 3 biological replicates. (B) Flow cytometry histograms depicting FITC fluorescence intensity in WT, RBM41 KO, and RBM41 OE cells under + Glc and - Glc conditions, n = 3 biological replicates. (C) Mean fluorescence intensity of ROS probe in WT, RBM41 KO, and RBM41 OE cells under + Glc and - Glc conditions, n = 3 biological replicates.

Error bars represent mean ± SD. Significance was determined using a two-way ANOVA with Šídák's multiple comparisons test (A, B). P values are as indicated.

**Supplementary Figure 9**

**Supplementary Figure 9. RBM41 modulates the GSH/GSSG ratio under – Glc in TSCC cell.**

Levels of GSH, GSSG, and the GSH/GSSG ratio in WT, RBM41 KO, and RBM41 OE cells under + Glc and - Glc conditions, n = 3 biological replicates. Error bars represent mean ± SD. Significance was determined using a two-way ANOVA with Šídák's multiple comparisons test. P values are as indicated.

**Supplementary Figure 10**

**Supplementary Figure 10. Analysis of RBM41 KO, SLC7A11 KO, RBM41 and SLC7A11 double knockout (R&S DKO) SCC-15 cells.**

(A, B) Western blot analysis of SLC7A11 protein levels in normal cell lines (293T, HOK, HOEC) and TSCC cell lines (Cal-27, SCC-9, SCC-15). (C) Sanger sequencing alignment confirming a 62 bp deletion in the SLC7A11 KO cells, with reference wild-type sequence shown for comparison. (D) Schematic of the CRISPR/Cas9 strategy for targeted deletion of the SLC7A11 genomic region and the associated Sanger sequencing chromatograms. (E - G) qRT-PCR (E) and Western blot (F, G) analysis of RBM41 and SLC7A11 transcript and protein expression in SCC-15 WT, RBM41 KO, SLC7A11 KO and R&S DKO cells, n = 3 biological replicates. (H) CCK-8 assay measuring cell viability of WT, RBM41 KO, SLC7A11 KO and R&S DKO cells under - Glc 24h, n = 6 biological replicates. (I, J) Flow cytometry analysis (I) and quantification (J) cell death in WT, RBM41 KO, SLC7A11 KO and R&S DKO cells after 12 hours of - Glc, n = 3 biological replicates.

Error bars represent mean ± SD. Significance was determined using a one-way ANOVA with Tukey's multiple comparisons test (B, E, G, H, J). P values are as indicated.

**Supplementary Figure 11**

**Supplementary Figure 11. Analysis of RNA - binding features and conservation of RBM41.**

(A) Distribution of the number of annotated RNA - binding domains in proteins. The y - axis represents the number of proteins, and the x - axis represents the count of annotated RNA - binding domains. (B) The most frequently detected RNA - binding domain, identified by Pfam ID (PF00076), with the y - axis indicating the number of proteins and the x - axis showing the specific Pfam ID. (C) Frequency of detection of the most prominent RNA - binding motif, with RBM41 highlighted; the y - axis denotes the RNA motif - containing protein (RBM41) and the x - axis shows the number of detection times. (D) Conservation of interactions across species, comparing rat and zebrafish; the y - axis represents the number of interactions, and the x - axis indicates the organism (rat or zebrafish).

**Supplementary Figure 12**

**Supplementary Figure 12. Sanger sequencing chromatograms identifying the knockout of the RRM1 domain in SCC-15 cells.**

The Sanger sequencing chromatograms for monoclonal cells #1 to #9 are shown, with red arrows indicating the positions where the base deletions begin.

**Supplementary Figure 13**

**Supplementary Figure 13. The situation of amino acid truncation after the base deletions in monoclonal cells #1 to #9.**

The figure illustrates the predicted amino acid changes following gene editing in cells from CL 1 to CL 9. The red amino acids indicate regions with identical amino acids..

**Supplementary Figure 14**

**Supplementary Figure 14. RRM 1 domain modulates the cell proliferation and apoptosis of SCC-9 cells under - Glc conditions.**

(A) CCK-8 assay measuring cell viability of WT, RBM41 KO, and RRM 1 KO cells under - Glc over time, n = 6 biological replicates. (B, C) Flow cytometry analysis (B) and quantification (C) cell death in WT, RBM41 KO, and RRM 1 KO cells after 9 hours of - Glc, n = 3 biological replicates.

Error bars represent mean ± SD. Significance was determined using a one-way ANOVA with Tukey's multiple comparisons test (C) or two-way ANOVA with Dunnett's multiple comparisons test (A). P values are as indicated.

**Supplementary Figure 15**

**Supplementary Figure 15. RRM 1 domain modulates the glucose uptake capacity of SCC-9 cells and the ATP level under - Glc conditions.**

(A) Glucose uptake assay in WT, RBM41 KO, and RRM 1 KO cell lines, n = 3 biological replicates. (B) Intracellular ATP levels in WT, RBM41 KO, and RRM 1 KO cell lines, n = 3 biological replicates. (C) NADP+/NADPH ratio in WT, RBM41 KO, and RRM 1 KO cell lines, n = 3 biological replicates.

Error bars represent mean ± SD. Significance was determined using a one-way ANOVA with Tukey's multiple comparisons test (A) or two-way ANOVA with Tukey's multiple comparisons test (B, C). P values are as indicated.

**Supplementary Figure 16**

**Supplementary Figure 16. The expression of CKMT1 in various cell lines.**

(A) Western blot analysis of CKMT1 protein levels in normal cell lines (293T, HOK, HOEC) and TSCC cell lines (Cal-27, SCC-9, SCC-15). (B) Statistical analysis of the western blot for CKMT1, n = 3 biological replicates.

Error bars represent mean ± SD. Significance was determined using a one-way ANOVA with Tukey's multiple comparisons test (A). P values are as indicated.

**Supplementary Figure 17**

**Supplementary Figure 17. CKMT1 is a downstream effector of RBM41 to suppress disulfidptosis in TSCC.**

(A) Sanger sequencing alignment confirming a 8 bp deletion in the CKMT1 KO cells, with reference wild-type sequence shown for comparison. (B) Schematic of the CRISPR/Cas9 strategy for targeted deletion of the CKMT1 genomic region and the associated Sanger sequencing chromatograms. (C - E) qRT-PCR (C) and Western blot (D, E) analysis of CKMT1 transcript and protein expression in SCC-15 WT and CKMT1 KO cells, n = 3 biological replicates. (F, G) Flow cytometry analysis (F) and quantification (G) cell death in WT and CKMT1 KO cells after 12 hours of - Glc, n = 3 biological replicates. (H) Intracellular ATP levels in SCC-15 cells under normal and - Glc conditions, n = 3 biological replicates. (I) NADP+/NADPH ratio in SCC-15 cells under - Glc 4 hours.

Error bars represent mean ± SD. Significance was determined using a Student's t-test (C, E) or one-way ANOVA with Dunnett's multiple comparisons test (G, H, I). P values are as indicated.

**Supplementary Figure 18**

**Supplementary Figure 18. Overview of experimental groups and detected tissues.**

H&E staining of submandibular lymph nodes, lung, liver, and brain.

**Supplementary Figure 19**

**Supplementary Figure 19. CCK - 8 Analysis of** **L937-0176 and L977-1488 in SCC-15 cell.**

(A) CCK-8 assay assessing cell proliferation in SCC-15 cells after L937-0176 treatment in + Glc. (B) CCK-8 assay assessing cell proliferation in SCC-15 cells after L937-0176 treatment in - Glc. (C) CCK-8 assay assessing cell proliferation in SCC-15 cells after L977-1488 treatment in + Glc. (D) CCK-8 assay assessing cell proliferation in SCC-15 cells after L977-1488 treatment in - Glc. Error bars represent mean ± SD, n = 3 biological replicates. Significance was determined using a one-way ANOVA with Dunnett's multiple comparisons test. P values are as indicated.

**Supplementary Figure 20**

**Supplementary Figure 20. Statistical analysis of the grayscale Western Blot for Figure 8J.**

Error bars represent mean ± SD. Significance was determined using a one-way ANOVA with Dunnett's multiple comparisons test. P values are as indicated.

**Supplementary Table 1.** List of the sgRNA sequences for CRISPR/Cas9.

| Name | Column heading (unit) | Column heading (unit) | PAM |
| --- | --- | --- | --- |
| RBM41  RBM41 | SgRNA 1 | AAGAAAGAGAGCTTTGCTCC | TGG |
|  | SgRNA 2 | CCCAGCTGCTTCCTTCCCAA | AGG |
| RBM41 | SgRNA 3 | ATGAAACAGAAATCTTGATC | TGG |
| RRM 1 | SgRNA 1 | ATCATTCGGAATTGAATTGG | AGG |
| RRM 1 | SgRNA 2 | ATGATATGAGAGAAGTCGCT | TGG |
| SLC7A11 | SgRNA 1 | TGGGCAACAAGGAGCCACCT | GGG |
| SLC7A11 | SgRNA 2 | AGGGGAGTCTCCATTATCAT | TGG |
| CKMT1 | SgRNA 1 | TGACCCCAGCAGTCTATGCA | CGG |
| CKMT1 | SgRNA 2 | GAGCCGTGCATAGACTGCTG | GGG |

**Supplementary Table 2.** List of the siRNA sequences.

| Name | Sense Strand (5’-3’) | Antisense Strand (5’-3’) |
| --- | --- | --- |
| RBM41-Homo-200 | GCUUUGCUCCUGGUACUAUTT | AUAGUACCAGGAGCAAAGCTT |
| RBM41-Homo-807 | CCAUGUGGCAAAUGUUAUUTT | AAUAACAUUUGCCACAUGGTT |

**Supplementary Table 3.** List of the primer sequences for RT-qPCR or PCR.

| Name | Forward primer (5’-3’) | Reverse primer (5’-3’) |
| --- | --- | --- |
| CKMT1  GAPDH | AGGCAAATCAGAGGTGGAGC | GGATGACAGGTGTGGGGATG |
|  | TGGTATCGTGGAAGGACTCA | GGGCCATCGACAGTCTTC |
| METTL8 | TGAAGAGAAGGCGAGAGAATCA | GGCACAGTAGGACAGTGCAT |
| MYO18A | CTCCAAGCGTGAATCCAAGAC | CCGGTTACTATCGGAGTCAATG |
| NBAS | CTGTTCAAGATCAGTGTGTGGAA | ACTCCATGCTACCCGTCTCC |
| RBM12 | TGAACTGAGTCGTAGGCGTTT | GGGCAAGTTTACCCTGCTACT |
| RBM12B | CTTCACGGGATTGACTATTCCTG | TTATGGCACGTCTTGCATCTT |
| RBM25 | AAGTTGATGCAAAGACAAAGGCA | TCGTCATTAGTGACAGTTTCTGG |
| RBM41 | AGAGCCCATCATTACTCCAGG | CCAGTCCAACACTGCTCAGG |
| RBMFOX2 | TACAGTGACGGTTATGGCAGG | CCTCGGTATAAACTCGCCACA |
| RBMS1 | ATGGGCAAAGTGTGGAAACAG | CTTGGCTTGCAGATACTGGGG |
| RBMS2 | TCTCAAACCTCCCACTGTCAA | ATACGGGTGGAGATAACCTGG |
| SCAF8 | CCACTGCTATGAGCAATACTCC | GGATCAGGTAAGCCTTGGACC |
| SLC7A11 | TCTCCAAAGGAGGTTACCTGC | AGACTCCCCTCAGTAAAGTGAC |
| SPEN | AACTCGGTCAACAAAATGGGT | AAACCTCTCTACTACGAGATGCT |
| β-actin | GTGGCCGAGGACTTTGATTG | AGTGGGGTGGCTTTTAGGATG |
| RBM41-PCR | CCTGGATGACGGCACTCTC | AATGAAGATATTTTGCTCTG |
| RRM 1-PCR | GAGTGCCTTACCTCCTTTAT | CCCCTCCCTCAGTTAGTTTT |
| SLC7A11-PCR | CAAATAGTGACAGGACCCCACA | GTGTCCACCATCTCCAAAGG |
| CKMT1-PCR | CCAACTGGACAGCTCCCAGG | CTCCTCATCTCCAGCCACCA |

**Supplementary Table 4.** List of the antibodies.

| Name | CHANG JIA | Cat. # |
| --- | --- | --- |
| CKMT1  Drebrin | Zenbio | 200627 |
|  | Zenbio | R381842 |
| Anti-Flag Tag (Mouse mAb) | Servicebio | GB15938-100 |
| Anti-Flag Tag (Rabbit mAb) | Servicebio | GB15939-100 |
| FLNA | Zenbio | R380781 |
| FLNB | CST | 12979T |
| GAPDH | Servicebio | ZB15004-HRP-100 |
| Ki67 | Zenbio | R381101 |
| MYH9 | Zenbio | R27092 |
| MYH10 | Zenbio | R27093 |
| RBM41 | Proteintech | 15784-1-AP |
| SLC7A11 | ABclonal | A13685SP |
| TOM20 | Proteintech | 1D6F5 |
| Tubulin | Servicebio | ZB15140-HRP-100 |
